# Why Bax detection in >1400 publications might be flawed

**DOI:** 10.1038/s41419-024-07273-6

**Published:** 2024-12-05

**Authors:** Kristin Entrop, Senait Wieske, Markus Rehm

**Affiliations:** 1 University of Stuttgart, Institute of Cell Biology and Immunology, Stuttgart, Germany; 2 University of Stuttgart, Stuttgart Research Center Systems Biology, Stuttgart, Germany

**Keywords:** Cell biology, Biochemistry

During apoptosis signaling, Bcl-2-associated X protein (Bax) translocates from the cytosol to the mitochondria to form pores in their outer membrane, allowing for the release of intermembrane proteins into the cytosol and thereby triggering apoptosis execution. Since its discovery in 1993 [1], Bax is, therefore, one of the most widely studied regulators of programmed cell death (>45,000 publications are returned on the search string “(Bax) AND (apoptosis)” on pubmed at the time of writing), with numerous studies investigating (changes in) expression amounts and localization of Bax in various scenarios of cellular stress conditions and in models for proliferative and degenerative diseases, using antibody-based assays. In contrast to comprehensively authenticated antibodies for clinical diagnostics, the sensitivity and specificity of antibody-based detections used in basic research are often far less thoroughly assessed by commercial suppliers for routine applications such as immunoblotting and immunofluorescence-based detection. One of the most widely used antibodies for Bax detection is a mouse monoclonal antibody raised against amino acids 1–171 of mouse Bax and with supposed reactivity to Bax of mouse, rat and human origin, available from Santa Cruz Biotechnology Inc. (Bax Antibody (B-9): sc-7480; https://www.scbt.com/p/bax-antibody-b-9). The use of this antibody, according to the manufacturer’s information, has been documented in >1400 scientific publications so far (see Supplementary Information 1 for a list valid as of May 2024).

Here, we show that this reagent appears to be unreliable due to providing false-positive signals at the expected molecular weight of Bax in immunoblotting experiments and likewise provides false-positive signals in immunofluorescence-based detection of Bax expression. This calls into question the validity of Bax detection in the above mentioned large body of literature. Firstly, we compared signals obtained by immunoblotting between wildtype and Bax/Bak-deficient HCT 116 cells, which are widely used model systems to compare conditions of apoptosis competency vs. resistance. To our surprise, Bax Antibody (B-9) provided strong signals at an expected apparent molecular weight between 20 and 25 kDa in both wild-type and Bax/Bak deficient HCT 116 cells (Fig. 1A). A signal at the expected molecular weight of Bax was also obtained in Bax/Bak-deficient mouse embryonic fibroblasts (Fig. 1A), suggesting that false positive detection applies to both human and mouse conditions. In contrast, an alternative antibody purchased from Cell Signalling Technology (Bax Antibody #2772), suited solely for immunoblotting applications according to the manufacturer’s information, clearly indicated loss of Bax expression in the respective Bax deficient cells (Fig. 1A). We also failed to detect a loss in the supposed Bax signal in HCT 116 cells transfected with two independent siRNAs targeting Bax expression when using Santa Cruz Inc. Bax Antibody (B-9), yet a clear depletion of Bax signal was observed when performing the detection with the antibody from Cell Signaling Technology (Fig. 1B). These results therefore also demonstrate that the Bax Antibody (B-9) does not provide specific Bax signals overlapping with unspecific signals. We furthermore conducted immunofluorescence stainings using the Bax Antibody (B-9) in wildtype and Bax/Bak-deficient HCT 116 cells. Staining intensities were comparable between wildtype and Bax/Bak-deficient cells (Fig. 1C) and overall appeared fairly weak in comparison to signals obtained with a specific Bcl-xL antibody using identical protocols (Supplementary Information 2). All experiments were conducted using standard protocols for immunoblotting and immunofluorescence-based detection (see Supplementary Information 3), orienting also along manufacturer information regarding suggested reagent concentrations. Overall, we therefore conclude that the widely used Bax Antibody (B-9) provides false positive signals, which unfortunately in immunoblotting experiments are detected at a kDa range expected for true positive Bax signals.

**Fig. 1:**
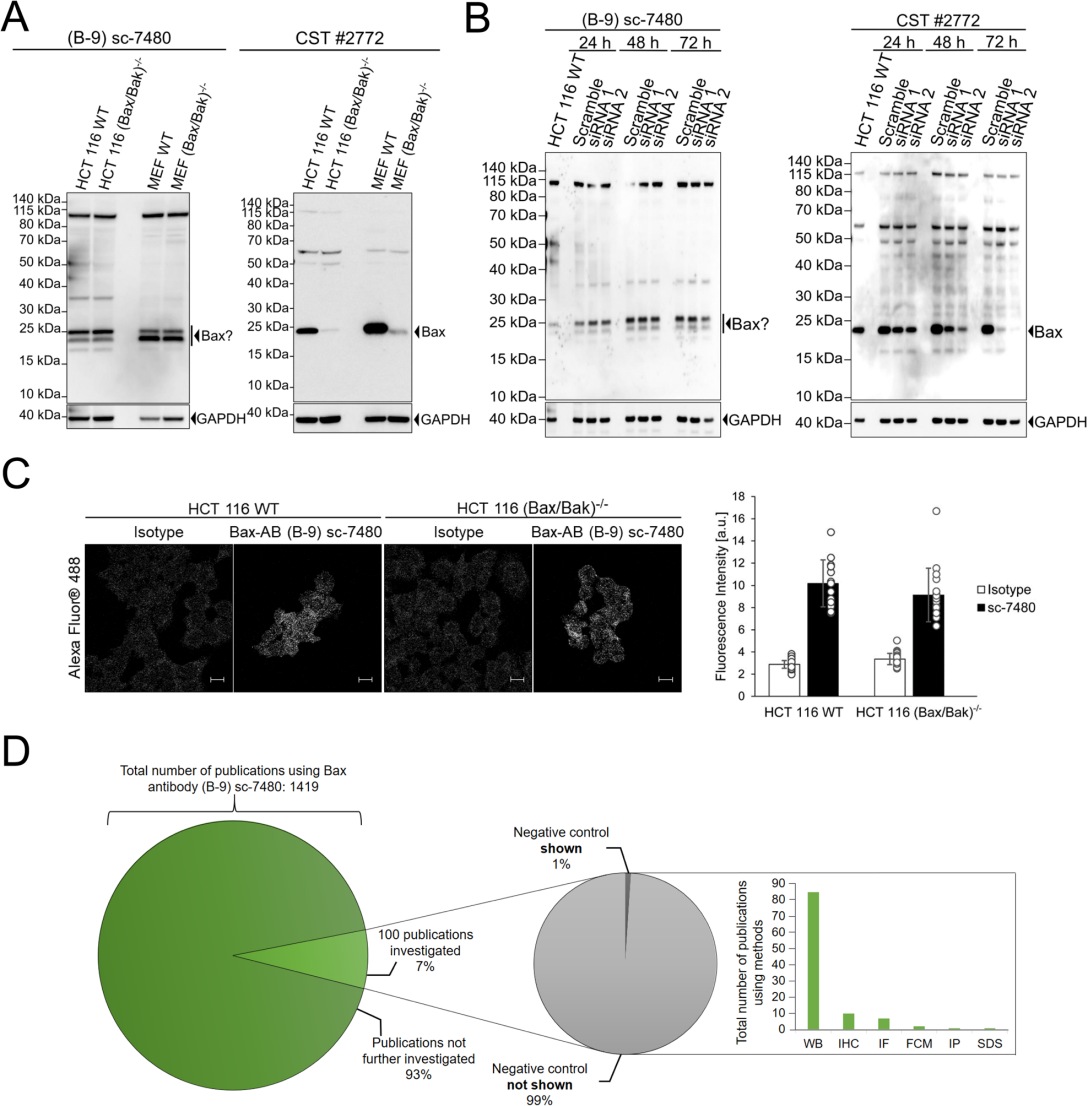
Validation of Bax antibody specificity. **A** Western blots showing signals obtained from whole cell extracts of parental and Bax-deficient cells, as indicated. GAPDH served as loading control. **B** Western blots showing signals obtained from whole cell extracts of HCT116 cells following transfection with siRNAs targeting human Bax. GAPDH served as loading control. **C** Immunofluorescence signals from wildtype and Bax-deficient HCT116 cells. Average intensities from single cells are shown in bar graphs as means ± s.d. **D** Literature analysis regarding the use of Bax antibody (B-9) sc-7480. Details are provided in the supplemental tables. Methods abbreviations: WB Western blotting, IHC immunohistochemistry, IF immunofluorescence, FCM flow cytometry, IP immunoprecipitation, SDS SDS-PAGE analysis. Full blots shown in this figure are provided as Supplemental Information 3.

From the pool of >1400 publications making use of Bax Antibody (B-9), we randomly selected 100 papers to screen these for the type of experimental techniques in which the antibody was used and whether or not controls were included to determine the specificity of Bax detection (Fig. 1D, Supplementary Information 4). The majority of experiments made use of immunoblotting, with other approaches such as immunofluorescence and immunohistochemistry being far less prominent. We noted that only one study presented an immunoblot suggesting specificity based on siRNA-based depletion of Bax expression (Fig. 1D, Supplementary Information 4). However, the methods description in said study does not provide information on the transfection procedure, the source or the sequence of the siRNA used and furthermore did not include molecular weight markers in the immunoblot display. In addition, we screened the citations listed in the product data-sheet available at https://datasheets.scbt.com/sc-7480.pdf, yet none of the listed studies included controls demonstrating reagent specificity (Supplementary Information 5). We did not specifically screen for publications making use of related products in which the antibody is available conjugated to agarose or various fluorophores. The development of hybridoma technologies for the large-scale generation of monoclonal antibodies [2, 3] paved the way for the continuous production of specific antibodies with defined specificity. It can therefore be assumed that the monoclonal Bax Antibody (B-9) on sale today is identical to that used in the very first publications referring to this reagent and needs to be considered a reagent that has been widely used but provides false-positive signals. Since the quality of antibody validation data as well as validation claims differ substantially across antibody vendors, we propose that care must be taken when using widely available commercial antibodies without further in-house validation for specificity. Still, especially with the ease by which siRNA-based depletion and genome editing methods can be applied, the burden to proof reagent quality should rather lie with the manufacturers and vendors.

## Supplementary information

Supplementary Information - all combined

Original Data
