# Supplementary material for: Why Bax detection in >1400 publications might be flawed
Source: Cell Death Dis. 2024 Dec 5;15(12):880. doi: 10.1038/s41419-024-07273-6 (PMC11621539; doi:10.1038/s41419-024-07273-6)
Supplement: Supplementary file 1 — Supplementary Information - all combined [file 41419_2024_7273_MOESM1_ESM.pdf]

Supplementary Information 1: Complete list of literature references for the use of Bax antibody (B-9): sc-7480 obtained from https://www.scbt.com/de/p/bax-antibody-b-9 as of 13.05.2024; 17:30 OESZ

Count	PMID	Description
1	10037601	p53 and ATM Dependent Apoptosis Induced by Telomeres Lacking TRF2 Karlseder, J. et al. 1999. Science. 283: 1321-1325.
2	10075662	Activation of NF-kappaB by RANK requires tumor necrosis factor receptor-associated factor (TRAF) 6 and NF-kappaB-inducing kinase. Identification of a novel TRAF6 interaction motif. Darnay, BG. et al. 1999. J. Biol. Chem. 274: 7724-31.
3	10110120200225964601	Modular biomaterials vaccine technology protects against multiple pathogens and septic shock Meng, Q. et al. 2022. Sci Rep. 12: 22360.
4	10206987	Endostain Induces Endothelial Cell Apoptosis Dhanabal, M. et al. 1999. J. Biol. Chem. 274: 11721-11726.
5	10449754	Bcl-2 family members do not inhibit apoptosis by binding the caspase activator Apaf-1. Moriishi, K. et al. 1999. Proc. Natl. Acad. Sci. U.S.A. 96: 9683-8.
6	10501549	Localization of survival motor neuron protein in human apoptotic-like and regenerating muscle fibers, and neuromuscular junctions. Neuroreport. 10: 1637-41.
7	10652358	Role of tyrosine phosphorylation in the regulation of the interaction of heterogenous nuclear ribonucleoprotein K protein with its protein and RNA partners. Ostrowski, J. et al. 2000. J. Biol. Chem. 275: 3619-28.
8	10734238	The amyloid beta peptide abeta (25-35) induces apoptosis independent of p53. Blasko, I. et al. 2000. FEBS Lett. 470: 221-5.
9	10741285	Changes in p21WAF1, pRb, Mdm-2, Bax and Bcl-2 expression in cervical cancer cell lines transfected with a p53 expressing adenovirus. Huang, TG. et al. 2000. Eur. J. Cancer. 36: 249-56.
10	10744705	Physical interaction between Wilms tumor 1 and p73 proteins modulates their functions. Scharnhorst, V. et al. 2000. J. Biol. Chem. 275: 10202-11.
11	10772908	Bcl-2 antibodies induce hemoglobin release by red blood cells loaded with in vitro translated Bcl-2 and its cleaved fragment. Chen, C. et al. 2000. Biochem. Biophys. Res. Commun. 270: 816-20.
12	10773823	Bcl-2 and Bax proteins are present in interphase nuclei of mammalian cells. Hoetelmans, R. et al. 2000. Cell Death Differ. 7: 384-92.
13	10802378	Transfection with mutant p53 gene inhibits heat-induced apoptosis in a head and neck cell line of human squamous cell carcinoma. Ota, I. et al. 2000. Int. J. Radiat. Oncol. Biol. Phys. 47: 495-501.
14	10851071	Proneness to UV-induced apoptosis in human fibroblasts defective in transcription coupled repair is associated with the lack of Mdm2 transactivation Conforti, G. et al. 2000. Oncogene. 19: 2714-2720.
15	10854211	Increased Exp[ression and Activation of Stress-Activated Protein Kinases/c-Jun NH2-Terminal Protein Kinases in Atheroclerotic Lesions Coincide with p53 Metzler, B. et al. 2000. Am. J. Pathology. 156: 1875-1886.
16	10960491	Surfactant modulates intracellular signaling of the adhesion receptor L-selectin. Brenner, B. et al. 2000. Pediatr Res. 48: 283-8.
17	10969781	Human THP-1 monocytic leukemic cells induced to undergo monocytic differentiation by bryostatin 1 are refractory to proteasome inhibitor-induced apoptosis. Chen, C. et al. 2000. Cancer Res. 60: 4377-85.
18	10972857	Apoptosis and apoptotic-related factors in mucoepidermoid carcinoma of the oral minor salivary glands. Yin, HF. et al. 2000. Pathol. Int. 50: 603-9.
19	11029754	N-terminal cleavage of bax by calpain generates a potent proapoptotic 18-kDa fragment that promotes bcl-2-independent cytochrome C release and apoptotic cell death. Gao, G. et al. 2000. J. Cell. Biochem. 80: 53-72.
20	11042339	Expression of bcl-2, bax, and caspase-3 in the brain of the developing rat. Mooney, SM. et al. 2000. Brain Res. Dev. Brain Res. 123: 103-17.
21	11146441	Arsenic trioxide induces apoptosis in human gastric cancer cells through up-regulation of p53 and activation of caspase-3. Jiang, XH. et al. 2001. Int. J. Cancer. 91: 173-9.
22	11169978	Hepatocyte growth factor induces pro-apoptotic genes in HepG2 hepatoma but not in B16-F1 melanoma cells. Matteucci, E. et al. 2001. J. Cell. Physiol. 186: 387-96.
23	11226429	Inhibition of caspase-1 induces cell death in pancreatic carcinoma cells and potentially modulates expression levels of bcl-2 family proteins. Schlosser, S. et al. 2001. FEBS Lett. 491: 104-8.
24	11236928	Different inducibility of radiation- or heat-induced p53-dependent apoptosis after acute or chronic irradiation in human cultured squamous cell carcinoma cells. Takahashi, A. et al. 2001. Int. J. Radiat. Biol. 77: 215-24.
25	11267863	Effects of a novel synthetic retinoid on malignant glioma in vitro: inhibition of cell proliferation, induction of apoptosis and differentiation. Costa, SL. et al. 2001. Eur. J. Cancer. 37: 520-30.
26	11271628	Comparison of apoptosis and apoptosis-related gene products between extranodal oral B-cell lymphoma and maxillofacial nodal B-cell lymphoma. Yin, H. et al. 2001. J. Oral Pathol. Med. 30: 141-7.
27	11331249	Human RERE is localized to nuclear promyelocytic leukemia oncogenic domains and enhances apoptosis. Cell Growth Differ. 12: 201-10.
28	11337381	Increased Osteoblast Apoptosis in Apert Craniosynostosis. Role of Protein Kinase C and Interleukin-1. Lemonnier, J. et al. 2001. Am. J. Pathology. 158: 1833-1842.
29	11340567	Radiation-induced apoptosis in human myeloma cell line increases BCL-2/BAX dimer formation and does not result in BAX/BAX homodimerization. Filippovich, IV. et al. 2001. Int. J. Cancer. 92: 651-60.
30	11343245	Fas-induced apoptosis in mouse hepatocytes is dependent on C/EBPbeta. Mukherjee, D. et al. 2001. Hepatology. 33: 1166-72.
31	11397703	Oxidized LDL modulates Bax/Bcl-2 through the lectinlike Ox-LDL receptor-1 in vascular smooth muscle cells Kataoka, H. et al. 2001. Arterioscler Thromb Vasc Biol. 21: 955-960.
32	11421874	Expression of hepatocyte growth factor, transforming growth factor alpha, apoptosis related proteins Bax and Bcl-2, and gastrin in human gastric cancer. Konturek, PC. et al. 2001. Aliment. Pharmacol. Ther. 15: 989-99.
33	11477453	The cytotoxic effect of E1B 55-kDa mutant adenovirus on human hepatocellular carcinoma cell lines. Zhao, J. et al. 2001. Cancer Gene Ther. 8: 333-41.
34	11489446	Effects of prenatal exposure to ethanol on the expression of bcl-2, bax and caspase 3 in the developing rat cerebral cortex and thalamus. Mooney, SM. et al. 2001. Brain Res. 911: 71-81.
35	11500052	Appearance of shortened Bcl-2 and Bax proteins and lack of evidence for apoptosis in rat forebrain after severe experimental traumatic brain injury. Xiong, Y. et al. 2001. Biochem. Biophys. Res. Commun. 286: 401-5.
36	11509323	Apoptosis in neonatal murine lung exposed to hyperoxia. McGrath-Morrow, SA. et al. 2001. Am. J. Respir. Cell Mol. Biol. 25: 150-5.
37	11517246	Recruitment of the mitochondrial-dependent apoptotic pathway in amyotrophic lateral sclerosis Guegan, C. et al. 2001. J Neurosci. 21(17): 6569-6576.
38	11576453	Radiation response of apoptosis in C57BL/6N mouse spleen after whole-body irradiation. Takahashi, A. et al. 2001. Int. J. Radiat. Biol. 77: 939-45.
39	11592846	GDNF protects against aluminum-induced apoptosis in rabbits by upregulating Bcl-2 and Bcl-XL and inhibiting mitochondrial Bax translocation. Ghribi, O. et al. 2001. Neurobiol. Dis. 8: 764-73.
40	11641418	Enhanced acetaminophen hepatotoxicity in transgenic mice overexpressing BCL-2. Adams, ML. et al. 2001. Mol. Pharmacol. 60: 907-15.
41	11694525	Restoration of Transforming Growth Factor- Signaling Enhances Radiosensitivity by Altering the Bcl-2/Bax Ratio in the p53 Mutant Pancreatic Cancer Cell Line MIA PaCa-2* Ahmed, M. et al. 2002. J. Biol. Chem. 277: 2234-2246.
42	11694645	Short-chain fatty acids inhibit invasive human colon cancer by modulating uPA, TIMP-1, TIMP-2, mutant p53, Bcl-2, Bax, p21 and PCNA protein expression in an in vitro cell culture model. Emenaker NJ et al. 2001. J Nutr. 131: 3041S-6S.
43	11699554	Apoptosis signals in sporadic amyotrophic lateral sclerosis: an immunocytochemical study. Embacher, N. et al. 2001. Acta Neuropathol. 102: 426-434.
44	11730720	Effects and mechanisms of aloe-emodin on cell death in human lung squamous cell carcinoma Lee HZ et al. 2001. Eur. J. Pharmacol. 431: 287-295.
45	11751403	Regulation of cyclin D1 and p16(INK4A) is critical for growth arrest during mammary involution. Gadd, M. et al. 2001. Cancer Res. 61: 8811-9.
46	11753684	Functional p53 is required for triptolide-induced apoptosis and AP-1 and nuclear factor-kappaB activation in gastric cancer cells Jiang, X et al. 2001. Oncogene. 20: 8009-8018.
47	11796515	Intracrine PTHrP protects against serum starvation-induced apoptosis and regulates the cell cycle in MCF-7 breast cancer cells. : Tovar Sepulveda, VA. et al. 2002. Endocrinology. 143(2): 596-606.
48	11851356	Apoptosis and post-infarction left ventricular remodeling. Baldi, A. et al. 2002. J. Mol. Cell. Cardiol. 34: 165-74.
49	11854168	Twist haploinsufficiency in Saethre--Chotzen syndrome induces calvarial osteoblast apoptosis due to increased TNFalpha expression and caspase-2 activation Yousfi, M. et al. 2002. Human Molecular Genetics. 11: 359-369.
50	11858462	Alteration of the Bcl-2:Bax ratio in the placenta as pregnancy proceeds. De Falco, M. et al. 2001. Histochem. J. 33: 421-5.
51	11864976	The course of etoposide-induced apoptosis from damage to DNA and p53 activation to mitochondrial release of cytochrome c. Karpinich, NO. et al. 2002. J. Biol. Chem. 277: 16547-52.
52	11883953	Bcl-X(L) and calyculin A prevent translocation of Bax to mitochondria during apoptosis. Ganju, N. et al. 2002. Biochem Biophys Res Commun. 291: 1258-64.
53	11911839	p53-Independent induction of Fas and apoptosis in leukemic cells by an adenosine derivative, Cl-IB-MECA. Kim, SG. et al. 2002. Biochem. Pharmacol. 63: 871-80.
54	11915028	The in vivo apoptotic effect of interferon alfa-2b on rat preneoplastic liver involves Bax protein. de Lujan Alvarez, M. et al. 2002. Hepatology. 35: 824-833.
55	11931841	Bcl-2 family-mediated apoptotic effects of 3,3'-diindolylmethane (DIM) in human breast cancer cells. Hong, C. et al. 2002. Biochem. Pharmacol. 63: 1085-97.
56	11997250	Regulation of apoptosis in the endocardial cushions of the developing chick heart Keyes, WM. et al. 2002. Am J Physiol Cell Physiol. 282(6): C1348-1360.
57	12007567	Down-modulation of Bcl-XL, release of cytochrome c and sequential activation of caspases during honokiol-induced apoptosis in human squamous lung cancer CH27 cells Yang SE et al. 2002. Biochem. Pharmacol. 63: 1641-1651.
58	12065092	Regulation of Bcl-2 family molecules and activation of caspase cascade involved in gypenosides-induced apoptosis in human hepatoma cells. Wang QF et al. 2002. Cancer Lett. 183: 169-78.
59	12090934	Flavones from Scutellaria baicalensis Georgi attenuate apoptosis and protein oxidation in neuronal cell lines. Choi, J. et al. 2002. Biochim. Biophys. Acta. 1571: 201-10.
60	12091474	Lithium inhibits aluminum-induced apoptosis in rabbit hippocampus, by preventing cytochrome c translocation, Bcl-2 decrease, Bax elevation and caspase-3 activation. Ghribi, O. et al. 2002. J. Neurochem. 82: 137-45.
61	12097139	Biphasic translocation of Bax to mitochondria Capano, M. et al. 2002. Biochem J. 367(Pt 1): 169-178.
62	12106610	Modulation of tamoxifen-induced apoptosis by peripheral benzodiazepine receptor ligands in breast cancer cells. Strohmeier R et al. 2002. Biochem Pharmacol. 64: 99-107.
63	12107341	Expression of Bcl-2 and Bax in hypokalemic nephropathy in rats. Kimura, T. et al. 2001. Pathobiology. 69: 237-48.
64	12115564	Effector mechanisms of norcantharidin-induced mitotic arrest and apoptosis in human hepatoma cells. Chen YN et al. 2002. Int J Cancer. 100: 158-65.
65	12118068	Selective impairment of p53-mediated cell death in fibroblasts from sporadic Alzheimer's disease patients Uberti, D. et al. 2002. J. Cell Sci. 115: 3131-3138.
66	12164688	Proliferation and apoptosis in long-term surviving low grade gliomas in relation to radiotherapy. J. Neurooncol. 58: 157-65.
67	12173037	Activation of NF-kappaB and upregulation of intracellular anti-apoptotic proteins via the IGF-1/Akt signaling in human multiple myeloma cells: therapeutic implications. Mitsiades CS, et al. 2002. Oncogene. 21: 5673-7683.
68	12197672	Vasoactive intestinal peptide stimulation modulates the expression of Bcl-2 family members in the adrenal gland of the lizard Podarcis sicula. De Falco, M. et al. Histochem. J. 33: 639-45.
69	12198774	Negative correlation between the ratio of Bax to Bcl-2 and the size of tumor treated by culture supernatants from Kupffer cells. Chen, GG. et al. 2002. Clin. Exp. Metastasis. 19: 457-64.
70	12203123	Novel target for induction of apoptosis by cyclo-oxygenase-2 inhibitor SC-236 through a protein kinase C-beta1-dependent pathway Jiang, X.H. et al. 2002. Oncogene. 21: 6113-6122.
71	12213291	4-Fluoro-N-butylphenylacetamide: a synthetic phenylacetate derivative that upregulates Bcl-X(S), activates caspase cascade and induces apoptosis in human squamous lung cancer CH27 cells. Chan HC et al. 2002. Cancer Lett. 186: 211-21.
72	12213439	Instrumental activation of bid by caspase-1 in a transgenic mouse model of ALS. Guégan, C. et al. 2002. Mol. Cell. Neurosci. 20: 553-62.
73	12237847	TGF-beta induces cell death in the oligodendroglial cell line OLI-neu. Schuster, N. et al. 2002. Glia. 40: 95-108.
74	12372900	N-tosyl-L-phenylalanyl-chloromethyl ketone eliminates the increase in caspase-3 and bcl-2 caused by brain injury in the newborn rat. Feng, Y. et al. 2002. Pharmacology. 66: 115-9.
75	12431242	Retinoid-induced apoptosis in B-cell chronic lymphocytic leukaemia cells is mediated through caspase-3 activation and is independent of p53, the retinoic acid receptor, and differentiation. Pepper C et al. 2002. Eur J Haematol. 69: 227-35.
76	12446453	The vitamin D3 analog EB1089 induces apoptosis via a p53-independent mechanism involving p38 MAP kinase activation and suppression of ERK activity in B-cell chronic lymphocytic leukemia cells in vitro Pepper, C. et al. 2003. Blood. 101: 2454-2460.
77	12473386	Merkel cell carcinomas infiltrated with CD33+ myeloid cells and CD8+ T cells are 4associated with improved outcome T, et al. 2017. Jaad. 65: 121-9.
78	12481899	Antisense oligonucleotides complementary to Bax transcripts reduce the susceptibility of B-cell chronic lymphocytic leukaemia cells to apoptosis in a bcl-2 independent manner. Pepper C et al. 2002. Leuk Lymphoma. 43: 2003-9.
79	12522090	Effector mechanism of magnolol-induced apoptosis in human lung squamous carcinoma CH27 cells Yang, S.E. et al. 2003. Br. J. Pharmacol. 138: 193-201.
80	12531875	Defective TNF-alpha-mediated hepatocellular apoptosis and liver damage in acidic sphingomyelinase knockout mice Garcia-Ruiz, C. et al. 2003. J. Clin. Invest. 111: 197-208.
81	12558993	Vitamin E blocks early events induced by 1-methyl-4-phenylpyridinium (MPP+) in cerebellar granule cells. Gonzalez-Polo, R.A. et al. 2003. J Neurochem. 84: 305-15.
82	12563316	Activated protein C blocks p53-mediated apoptosis in ischemic human brain endothelium and is neuroprotective Cheng, T. et al. 2003. Nature Medicine. 9: 338-342.
83	12595970	Evaluation of warm ischemia-reperfusion injury using heat shock protein in the rat liver. Sakai, T. et al. 2003. Transpl. Int. 16: 88-99.
84	12614281	Involvement of the mitochondrial pathway in cold storage and rewarming-associated apoptosis of human renal proximal tubular cells. Salahudeen, AK. et al. 2003. Am. J. Transplant. 3: 273-80.
85	12624600	Glioma apoptosis induced by macrophages involves both death receptor-dependent and independent pathways Chen, G.G. et al. 2003. J Lab Clin Med. 141: 190-199.
86	12629556	Adipose tissue cellularity and apoptosis after intracerebroventricular injections of leptin and 21 days of recovery in rats. Gullicksen, PS. et al. 2003. Int. J. Obes. Relat. Metab. Disord. 27: 302-12.
87	12637528	Smad3 Potentiates Transforming Growth Factor beta (TGFbeta)-induced Apoptosis and Expression of the BH3-only Protein Bim in WEHI 231 B Lymphocytes Wildey, G.M. et al. 2003. J. Biol. Chem. 278: 18069-18077.
88	12644821	Evaluation of clinical significance of TP53, BCL-2, BAX and MEK1 expression in 229 ovarian carcinomas treated with platinum-based regimen Kupryjanczyk, J. et al. 2003. Br J Cancer. 88: 848-854.
89	12695689	Leukemic and non-leukemic lymphocytes from patients with Li Fraumeni syndrome demonstrate loss of p53 function. Bcl-2 family dysregulation and intrinsic resistance to conventional chemotherapeutic drugs but not flavopiridol. Pepper C et al. 2003. Cell Cycle. 2: 53-8.
90	12700662	Antiviral agent Cidofovir decreases Epstein-Barr virus (EBV) oncoproteins and enhances the radiosensitivity in EBV-related malignancies Abdulkarim B, et al. 2003. Oncogene. 22: 2260-2271.
91	12706452	A phenylacetate derivative, SCK6, inhibits cell proliferation via G1 cell cycle arrest and apoptosis Chan, H.C. et al. 2003. Eur. J. Pharmacol. 467: 31-39.
92	12714668	Protective effect of docosahexaenoic acid on oxidative stress-induced apoptosis of retina photoreceptors. Rotstein, NP. et al. 2003. Invest. Ophthalmol. Vis. Sci. 44: 2252-9.
93	12721309	Regulation of Bcl-xL Expression by H2O2 in Cardiac Myocytes Valks, D.M. et al. 2003. J. Biol. Chem. 278: 25542-25547.
94	12753152	Differential, stage-dependent expression of Hsp70, Hsp110 and Bcl-2 in colorectal cancer. Hwang, TS. et al. 2003. J. Gastroenterol. Hepatol. 18: 690-700.
95	12791942	Different expression patterns of Bcl-2, Bcl-xl, and Bax proteins after sublethal forebrain ischemia in C57Black/Crj6 mouse striatum Wu, C. et al. 2003. Stroke. 34: 1803-1808.
96	12810152	Biochemical studies of apoptosis induced by tamoxifen in estrogen receptor positive and negative breast cancer cell lines. Salami, S. et al. 2003. Clin. Biochem. 36: 247-53.
97	12821940	Hepatocyte growth factor induces apoptosis through the extrinsic pathway in hepatoma cells: favouring role of hypoxia-inducible factor-1deficiency Matteucci E, et al. 2003. Oncogene. 22: 4062-4073.
98	12829850	Cooperative interactions of simian immunodeficiency virus Nef, AP-2, and CD3-zeta mediate the selective induction of T-cell receptor-CD3 endocytosis. Shin, MR. et al. 2017. J. Virol. 77: 8116-26.
99	12868066	Transforming growth factor-beta and tumor necrosis factor-alpha cooperate to induce apoptosis in the oligodendroglial cell line OLI-neu. Schuster, N. et al. 2003. J. Neurosci. Res. 73: 324-33.
100	12883359	Antiapoptotic action of 1alpha,25-dihydroxyvitamin D3 in primary human melanocytes Sauer, B et al. 2003. Melanoma Res. 13: 339-347.
101	12893773	The proteasome inhibitor bortezomib interacts synergistically with histone deacetylase inhibitors to induce apoptosis in Bcr/Abl+ cells sensitive and resistant to STI571. Yu C, et al. 2003. Blood. 102(10): 3765-3774.
102	12931104	The correlation between expression of apoptosis-related proteins and myocardial functional reserve evaluated by dobutamine stress echocardiography in patients with dilated cardiomyopathy. Ho, YL. et al. 2003. J Am Soc Echocardiogr. 16: 931-6.
103	14555963	Cell-cell contacts trigger programmed necrosis and induce cyclooxygenase-2 expression. Bizik, J. et al. 2004. Cell Death Differ. 11: 183-195.
104	14560002	Impaired regulation of tumor suppressor p53 caused by mutations in the xeroderma pigmentosum DDB2 gene: mutual regulatory interactions between p48(DDB2) and p53. Itoh, T. et al. 2003. Mol. Cell. Biol. 23: 7540-53.
105	14572466	Regulation of Bax translocation through phosphorylation at Ser-70 of Bcl-2 by MAP kinase in NO-induced neuronal apoptosis. Ishikawa, Y. et al. 2003. Mol. Cell. Neurosci. 24: 451-9.
106	14597184	Detection of DNA fragmentation and apoptotic proteins, and quantification of uncoupling protein expression by real-time RT-PCR in adipose tissue. Gullicksen, PS. et al. 2004. J. Biochem. Biophys. Methods. 58: 1-13.
107	14607278	Oxidation of indole-3-acetic acid by horseradish peroxidase induces apoptosis in G361 human melanoma cells. Kim, DS. et al. 2004. Cellular signalling. 16: 81-8.
108	14622098	Sequential activation of individual caspases, and of alterations in Bcl-2 proapoptotic signals in a mouse model of Huntington's disease. Zhang, Y. et al. 2003. J. Neurochem. 87: 1184-92.
109	14680805	Antisense BAG-1 sensitizes HeLa cells to apoptosis by multiple pathways. Xiong, J. et al. 2003. Biochem. Biophys. Res. Commun. 312: 585-91.
110	14695654	Toxic action of etoposide on mouse peritoneal macrophages and its modulation by interleukin 3. Olmos, G. et al. Cell Biochem. Funct. 22: 45-52.
111	14712225	The tumor suppressor p16(INK4a) gene is a regulator of apoptosis induced by ultraviolet light and cisplatin. Al-Mohanna, MA. et al. 2004. Oncogene. 23: 201-12.
112	14724652	P-glycoprotein-mediated drug efflux is a resistance mechanism of chronic myelogenous leukemia cells to treatment with imatinib mesylate. Illmer, T. et al. 2004. Leukemia. 18(3): 401-408.
113	14732211	Norepinephrine induces apoptosis in neonatal rat endothelial cells via down-regulation of Bcl-2 and activation of beta-adrenergic and caspase-2 pathways Fu, Y.C. et al. 2004. Cardiovasc. Res. 61: 143-151.
114	14977852	Imexon-induced apoptosis in multiple myeloma tumor cells is caspase-8 dependent. Evens, AM. et al. 2004. Clin. Cancer Res. 10: 1481-91.
115	14983013	The Bik BH3-only protein is induced in estrogen-starved and antiestrogen-exposed breast cancer cells and provokes apoptosis. Hur, J. et al. 2004. Proc. Natl. Acad. Sci. USA. 101: 2351-6.
116	14984262	A G-quadruplex ligand 3,3'-diethyloxadicarbocyanine iodide induces mitochondrion-mediated apoptosis but not decrease of telomerase activity in nasopharyngeal carcinoma NPC-TW01 cells. Li, CP. et al. 2004. Pharm Res. 21: 93-100.
117	14987955	Heyneanol A induces apoptosis via cytochrome c release and caspase activation in human leukemic U937 cells. Lee, EO. et al. 2004. Life Sci. 74: 2313-26.
118	14990581	Loss of MYC Confers Resistance to Doxorubicin-induced Apoptosis by Preventing the Activation of Multiple Serine Protease- and Caspase-mediated Pathways Grassilli, E. et al. 2004. J. Biol. Chem. 279: 21318-21326.
119	15020525	Cyclo-oxygenase-2 (COX-2) expression at the site of recent myocardial infarction: friend or foe? Abbate, A. et al. 2004. Heart. 90: 440-3.
120	15026553	Down-regulation of Bcl-2 is associated with cisplatin resistance in human small cell lung cancer H69 cells Kumar Biswas, S. et al. 2004. Mol Cancer Ther. 3: 327-334.
121	15048135	Molecular mechanisms of apoptosis in the liver of rats after portal branch ligation with and without retrorsine. Picard, C. et al. 2004. Lab. Invest. 84: 618-28.
122	15057313	Bim regulation may determine hippocampal vulnerability after injurious seizures and in temporal lobe epilepsy. Shinoda,S. et al. 2004. J. Clin. Invest. 113(7): 1059-1068.
123	15081873	A novel mechanism for pergolide-induced neuroprotection: inhibition of NF-kappaB nuclear translocation. Uberti, D. et al. 2004. Biochem. Pharmacol. 67: 1743-50.
124	15135649	The peroxisome proliferator BR931 kills FaO cells by p53-dependent apoptosis. Simbula, G. et al. 2004. Life Sci. 75: 271-86.
125	15146550	Characterization of salvicine-resistant lung adenocarcinoma A549/SAL cell line. Miao, ZH. et al. 2004. Int. J. Cancer. 110: 627-632.
126	15147889	Expression of estrogen receptor beta in prostate carcinoma cells inhibits invasion and proliferation and triggers apoptosis. Cheng, J. et al. 2004. FEBS Lett. 566: 169-72.
127	15155628	Lipopolysaccharide Protects Primary B Lymphocytes from Apoptosis by Preventing Mitochondrial Dysfunction and Bax Translocation to Mitochondria Souvannavong, V. et al. 2004. Infection and Immunity. 72: 3260-3266.
128	15172883	Minocycline inhibits apoptosis and inflammation in a rat model of ischemic renal injury. Am. J. Physiol. Renal Physiol. 287: F760-6.
129	15200158	Histopathological and molecular changes during apoptosis produced by 7H-dibenzo[c,g]-carbazole in mouse liver. Martín-Burriel, I. et al. Toxicol Pathol. 32: 202-11.
130	15205359	Ectopic expression of Bcl-XL or Ku70 protects human colon cancer cells (SW480) against curcumin-induced apoptosis while their down-regulation potentiates it Rashmi, R. et al. 2004. Carcinogenesis. 25: 1867-1877.
131	15210576	Antidiabetic drug miglitol inhibits myocardial apoptosis involving decreased hydroxyl radical production and Bax expression in an ischaemia/reperfusion rabbit heart. Wang, N. et al. 2004. Br. J. Pharmacol. 142: 983-90.
132	15246189	Expression of p53 protein and the apoptotic regulatory molecules Bcl-2, Bcl-XL, and Bax in locally advanced squamous cell carcinoma of the lung. Shabnam, MS. et al. 2004. Lung Cancer. 45: 181-8.
133	15277502	Expression of apoptosis markers in the retinas of human subjects with diabetes. Abu-El-Asrar, AM. et al. 2004. Invest. Ophthalmol. Vis. Sci. 45: 2760-6.
134	15314639	Abnormalities of apoptotic and cell cycle regulatory proteins in distinct histopathologic components of benign prostatic hyperplasia. Prostate Cancer Prostatic Dis. 7: 321-6.
135	15314656	The telomeric protein TRF2 binds the ATM kinase and can inhibit the ATM-dependent DNA damage response Karlseder J, et al. 2004. PLoS Biology. 2(8): e240.
136	15356200	The c-Jun N-terminal protein kinase signaling pathway mediates Bax activation and subsequent neuronal apoptosis through interaction with Bim after transient focal cerebral ischemia Okuno, S. et al. 2004. J Neurosci. 24(36): 7879-7887.
137	15369734	Methylene chloride fraction of Scutellaria barbata induces apoptosis in human U937 leukemia cells via the mitochondrial signaling pathway. Cha, YY. et al. 2004. Clin. Chim. Acta. 348: 41-8.
138	15377997	Dynamic evolution of the adenine nucleotide translocase interactome during chemotherapy-induced apoptosis Verrier, F. et al. 2004. Oncogene. 23: 8049-8064.
139	15386938	Damaging effect of cigarette smoke extract on primary cultured human umbilical vein endothelial cells and its mechanism. Yang, YM. et al. 2004. Biomed. Environ. Sci. 17: 121-34.
140	15453993	Higher calpastatin levels correlate with resistance to calpain-mediated proteolysis and neuronal apoptosis in juvenile rats after spinal cord injury. Wingrave, JM. et al. 2004. Journal of neurotrauma. 21: 1240-54.
141	15464845	The vaccinia virus B1R kinase induces p53 downregulation by an Mdm2-dependent mechanism. Santos, C.R. et al. 2004. Virology. 328: 254-265.
142	15466854	Pea3 Transcription Factor Cooperates with USF-1 in Regulation of the Murine bax Transcription without Binding to an Ets-binding Site Firlej, Virginie et al. 2005. J. Biol. Chem. 280: 887-898.
143	15476743	Extracellular signal-regulated kinase activation and Bcl-2 downregulation mediate apoptosis after gemcitabine treatment partly via a p53-independent pathway Chang, G.C. et al. 2004. Eur J Pharmacol. 502: 169-183.
144	15517879	Apoptosis-related gene expression affected by a GnRH analogue without induction of programmed cell death in LNCaP cells. Angelucci, C. et al. Anticancer Res. 24: 2729-38.
145	15523687	Ursolic acid induces apoptosis through mitochondrial intrinsic pathway and caspase-3 activation in M4Beu melanoma cells. Harmand, PO. et al. 2005. Int. J. Cancer. 114: 1-11.
146	15543558	Characterization of events associated with apoptosis/anoikis induced by snake venom metalloproteinase BaP1 on human endothelial cells. Díaz, C. et al. 2005. J. Cell. Biochem. 94: 520-8.
147	15547822	Expression of apoptosis-related genes after fetal tracheal occlusion in rabbits. De Paepe, ME. et al. 2004. J. Pediatr. Surg. 39: 1616-25.
148	15569402	Centrophenoxine improves chronic cerebral ischemia induced cognitive deficit and neuronal degeneration in rats. Liao, Y. et al. 2004. Acta Pharmacol. Sin. 25: 1590-6.
149	15589371	Redox modulation of human prostate carcinoma cells by selenite increases radiation-induced cell killing. Husbeck, B. et al. 2005. Free Radic. Biol. Med. 38: 50-7.
150	15590985	Thrombopoietin regulates proliferation, apoptosis, secretory activity and intracellular messengers in porcine ovarian follicular cells: involvement of protein kinase A Sirotkin, A.V. et al. 2004. J Endocrinol. 183: 595-604.
151	15661804	Human colon cancer cells lacking Bax resist curcumin-induced apoptosis and Bax requirement is dispensable with ectopic expression of Smac or downregulation of Bcl-XL Rashmi, R. et al. 2005. Carcinogenesis. 26: 713-723.
152	15728126	Farnesyltransferase inhibitor R115777 (Zarnestra, Tipifarnib) synergizes with paclitaxel to induce apoptosis and mitotic arrest and to inhibit tumor growth of multiple myeloma cells Zhu K, et al. 2005. Blood. 105(12): 4759-4766.
153	15728256	Cbl-mediated ubiquitination of alpha5 integrin subunit mediates fibronectin-dependent osteoblast detachment and apoptosis induced by FGFR2 activation Kaabeche, K. et al. 2005. J Cell Sci. 118: 1223-1232.
154	15737691	Hypersensitivity in DNA mismatch repair-deficient colon carcinoma cells to DNA polymerase reaction inhibitors. Takahashi, T. et al. 2005. Cancer Lett. 220: 85-93.
155	15778017	Dietary modulation of 7,12-dimethylbenz[a]anthracene (DMBA)-induced adrenal toxicity in female Sprague-Dawley rats. Fu, X. et al. 2005. Food Chem. Toxicol. 43: 765-74.
156	15800711	Peroxisome proliferator-activated receptors (PPAR) agonists affect cell viability, apoptosis and expression of cell cycle related proteins in cell lines of glial brain tumors. Strakova, N. et al. 2005. Neoplasma. 52: 126-36.
157	15809372	Novel cardioprotective role of a small heat-shock protein, Hsp20, against ischemia/reperfusion injury Fan, GC. et al. 2005. Circulation. 111: 1792-1799.
158	15827328	Resveratrol-induced apoptotic death in human U251 glioma cells. Jiang, H. et al. 2005. Mol. Cancer Ther. 4: 554-61.
159	15831769	Modulation of apoptotic pathways in intestinal mucosa during hibernation Fleck, C.C. et al. 2005. Am. J. Physiol. Regul. Integr. Comp. Physiol. 289: R586-R595.
160	15841044	Bcl-XL protein levels determine apoptotic index in pancreatic carcinoma. Sharma, J. et al. 2005. Pancreas. 30: 337-42.
161	15851100	Old yellow enzyme interferes with Bax-induced NADPH loss and lipid peroxidation in yeast. Reekmans, R. et al. 2005. FEMS Yeast Res. 5: 711-25.
162	15857606	Dietary flavonoids as proteasome inhibitors and apoptosis inducers in human leukemia cells. Biochem. Pharmacol. 69: 1421-32.
163	15868939	Involvement of Bax, Bcl-2 and caspase 3 in hydroxyurea- or etoposide-induced apoptosis of mouse interleukin-3-dependent lymphoma cells. Olmos G. et al. 2005. Anticancer Res. 25: 999-1007.
164	15869997	Influence of p53 and bcl-2 on chemosensitivity in benign and malignant prostatic cell lines. Serafin, AM. et al. Urol. Oncol. 23: 123-9.
165	15881612	Sphingosine 1-phosphate is involved in cytoprotective actions of calcitriol in human fibroblasts and enhances the intracellular Bcl-2/Bax rheostat Sauer, B. et al. 2005. Pharmazie. 60: 298-304.
166	15891997	Bax, a predictive marker for therapeutic response to preoperative chemoradiotherapy in patients with rectal carcinoma Chang, Hee Jin et al et al. 2005. Human Pathology. 36: 364-371.
167	15941375	Progesterone differentially regulates pro- and anti-apoptotic gene expression in cerebral cortex following traumatic brain injury in rats. Yao, XL. et al. 2005. J. Neurotrauma. 22: 656-68.
168	15941563	Emodin induces apoptosis in human lung adenocarcinoma cells through a reactive oxygen species-dependent mitochondrial signaling pathway Su, Y.T. et al. 2005. Biochem Pharmacol. 70: 229-241.
169	15944736	TAp63alpha induces apoptosis by activating signaling via death receptors and mitochondria. Gressner, O. et al. 2005. EMBO J. 24: 2458-2471.
170	15947793	MIF loss impairs Myc-induced lymphomagenesis. Talos, F. et al. 2005. Cell death and differentiation. 12: 1319-28.
171	15969986	Induction of thyroid papillary carcinoma cell proliferation by estrogen is associated with an altered expression of Bcl-xL Lee, M.L. et al. 2005. Cancer J. 11: 113-121.
172	15972297	FASEB J. 1504-6.
173	15975526	Glycerol enhances radiosensitivity in a human oral squamous cell carcinoma cell line (Ca9-22) bearing a mutant p53 gene via Bax-mediated induction of apoptosis. Imai, Y. et al. 2005. Oral Oncol. 41: 631-6.
174	15982327	IL-6, its receptors and its relationship with bcl-2 and bax proteins in infiltrating and in situ human breast carcinoma Garcia-Tunon, I. et al. 2005. Histopathology. 47: 82-89.
175	16012953	Bid is upstream of lysosome-mediated caspase 2 activation in tumor necrosis factor alpha-induced hepatocyte apoptosis. Guicciardi, ME. et al. 2005. Gastroenterology. 129: 269-84.
176	16018998	Low temperature protects mammalian cells from apoptosis initiated by various stimuli in vitro. Sakurai, T. et al. 2005. Exp. Cell Res. 309: 264-72.
177	16045454	Decreases in phosphoinositide-3-kinase/Akt and extracellular signal-regulated kinase 1/2 signaling activate components of spinal motoneuron death. Newbern, J. et al. 2005. J. Neurochem. 94: 1652-65.
178	16049172	Essential role for survivin in early brain development Jiang, Y. et al. 2005. J Neurosci. 25(30): 6962-6970.
179	16080521	Predictors of response to chemo-radiotherapy and radiotherapy for esophageal squamous cell carcinoma. Miyazaki, T. et al. Anticancer Res. 25: 2749-55.
180	16122904	Systemic inflammation induces apoptosis with variable vulnerability of different brain regions. Semmler, A. et al. 2005. J. Chem. Neuroanat. 30: 144-57.
181	16135788	The BRG1- and hBRM-associated factor BAF57 induces apoptosis by stimulating expression of the cylindromatosis tumor suppressor gene Wang L. et al. 2005. Mol. Cell. Biol. 25: 7953-7965.
182	16136583	Expression of PCNA, p53, Bax, and Bcl-X in oral poorly differentiated and basaloid squamous cell carcinoma: relationships with prognosis. Sampaio-Góes, FC. et al. 2005. Head Neck. 27: 982-9.
183	16195739	TAp73/Delta Np73 influences apoptotic response, chemosensitivity and prognosis in hepatocellular carcinoma. Cell Death Differ. 2005 Dec;(12): Müller, M. et al. 2005
184	16212980	Over-expression of p73beta results in apoptotic death of post-mitotic hNT neurons. Jiang, Y. et al. 2006. Journal of the neurological sciences. 240: 1-6.
185	16213662	Flavonoids purified from Rhus verniciflua Stokes actively inhibit cell growth and induce apoptosis in human osteosarcoma cells. Jang, HS. et al. 2005. Biochim. Biophys. Acta. 1726: 309-316.
186	16215989	Knocking down PML impairs p53 signaling transduction pathway and suppresses irradiation induced apoptosis in breast carcinoma cell MCF-7. Bao-Lei, T. et al. 2006. J. Cell. Biochem. 97: 561-71.
187	16227409	Role of p21waf1/cip1 in effects of oxaliplatin in colorectal cancer cells. Hata, T. et al. 2005. Mol. Cancer Ther. 4: 1585-94.
188	16235684	Ubiquitous expression of cyclooxygenase-2 in meningiomas and decrease in cell growth following in vitro treatment with the inhibitor celecoxib: potential therapeutic application. Ragel, BT. et al. 2005. J. Neurosurg. 103: 508-17.
189	16242145	Antiapoptotic effect of calcitonin gene-related peptide on oxidative stress-induced injury in H9c2 cardiomyocytes via the RAMP1/CRLR complex. Sueur, S. et al. 2005. J. Mol. Cell. Cardiol. 39: 955-63.
190	16242231	Effect of subchronic exposure to acrylamide induced on the expression of bcl-2, bax and caspase-3 in the rat nervous system. Li, SX. et al. 2006. Toxicology. 217: 46-53.
191	16311913	A herbal medicine for the treatment of lung cancer. Ranga, RS. et al. 2005. Mol. Cell. Biochem. 280: 125-33.
192	16365298	Complex I deficiency primes Bax-dependent neuronal apoptosis through mitochondrial oxidative damage. Perier, C. et al. 2005. Proc. Natl. Acad. Sci. USA. 102(52): 19121-19131.
193	16374552	Cyclin D1 overexpression induces epidermal growth factor-independent resistance to apoptosis linked to BCL-2 in human A431 carcinoma. Rieber, M. et al. 2006. Apoptosis. 11: 121-9.
194	16376985	Enhanced basal apoptosis in cultured term human cytotrophoblasts is associated with a higher expression and physical interaction of p53 and Bak. Hu, C. et al. Placenta. 27: 978-83.
195	16394635	Enhanced expression of transferrin receptor confers UV-resistance in human and monkey cells. Chen, Z. et al. 2005. J. Radiat. Res. 46: 443-51.
196	16403436	Depression of proteasome activities during the progression of cardiac dysfunction in pressure-overloaded heart of mice. Tsukamoto, O. et al. 2006. Biochem. Biophys. Res. Commun. 340: 1125-33.
197	16403632	Relationship between IL-10 and tumor markers in breast cancer patients Llanes-Fernandez, L. et al. 2006. Breast. 15(4): 482-489.
198	16423893	A multifunctional cytoprotective agent that reduces neurodegeneration after ischemia Jiang, ZG. et al. 2006. Proc. Natl. Acad. Sci. USA. 103(5): 1581-1586.
199	16430474	Cell cycle regulators and apoptosis-associated proteins in relation to proliferative activity and degree of apoptosis in HNPCC versus sporadic endometrial carcinoma. Histopathology. 48: 275-85.
200	16441440	Toxoplasma gondii inhibits ultraviolet light-induced apoptosis through multiple interactions with the mitochondrion-dependent programmed cell death pathway. Carmen, JC. et al. 2006. Cell. Microbiol. 8: 301-15.
201	16464633	Cell proliferation and apoptosis culminate in early stages of oral oncogenesis. Derka, S. et al. 2006. Oral Oncol. 42: 540-50.
202	16480951	2-Deoxy-D-ribose inhibits hypoxia-induced apoptosis by suppressing the phosphorylation of p38 MAPK. Ikeda, R. et al. 2006. Biochem. Biophys. Res. Commun. 342: 280-5.
203	16496378	Modulation of apoptosis in the mouse brain after morphine treatments and morphine withdrawal Emeterio, E.P. et al. 2006. J Neurosci Res. 83: 1352-1361.
204	16537564	Apoptosis in mitochondrial myopathies is linked to mitochondrial proliferation. Auré, K. et al. 2006. Brain. 129: 1249-59.
205	16537918	Overlapping Roles for Homeodomain-Interacting Protein Kinases Hipk1 and Hipk2 in the Mediation of Cell Growth in Response to Morphogenetic and Genotoxic Signals Isono K et al. 2006. Mol. Cell. Biol. 26(7): 2758-2771.
206	16572399	c-Myc-dependent etoposide-induced apoptosis involves activation of Bax and caspases, and PKCdelta signaling. Albihn, A. et al. 2006. J. Cell. Biochem. 98: 1597-614.
207	16585127	Death receptor and mitochondrial pathways are involved in germ cell apoptosis in an experimental model of autoimmune orchitis. Theas, MS. et al. 2006. Hum. Reprod. 21: 1734-1742.
208	16596265	Anandamide-induced apoptosis in Chang liver cells involves ceramide and JNK/AP-1 pathway. Giuliano, M. et al. 2006. International journal of molecular medicine. 17: 811-9.
209	16651429	Celastrol, a Triterpene Extracted from the Chinese "Thunder of God Vine," Is a Potent Proteasome Inhibitor and Suppresses Human Prostate Cancer Growth in Nude Mice Yang, H. et al. 2006. Cancer Res. 66: 4758-4765.
210	16702812	Octahedral Pt(IV) complex K101 induces apoptosis via ERK1/2 activation and the p53 pathway in human colon cancer cells. Kwon, YE. et al. 2006. Anticancer Drugs. 17: 553-8.
211	16709574	JNK- and p38 kinase-mediated phosphorylation of Bax leads to its activation and mitochondrial translocation and to apoptosis of human hepatoma HepG2 cells Kim, B.J. et al. 2006. J Biol Chem. 281: 21256-21265.
212	16716514	Modulation of brain apoptosis-related proteins by the opioid antagonist naltrexone in mice. San-Emeterio, E.P. et al. 2006. Neurosci Lett. 403: 276-279.
213	16730720	5α-dihydrotestosterone treatment induces metabolic changes associated with Polycystic ovary syndrome without interfering with hyPothalamic lePtin and glucocorticoid signaling Vital-Reyes, V. et al. 2006. Arch Biol Sci. 68(3): 473-481.
214	16732981	Apoptosis induced by short hairpin RNA-mediated STAT6 gene silencing in human colon cancer cells. Zhang, MS. et al. 2006. Chin. Med. J. 119: 801-8.
215	16751401	Group B Streptococcus Induces Macrophage Apoptosis by Calpain Activation Fettucciari, K. et al. 2006. J. Immunology. 176: 7542-7556.
216	16776338	Sequential and spatial profiles of apoptosis in ischemic penumbra after two-vein occlusion in rats. Nishioka, T. et al. 2006. J. Neurosurg. 104: 938-44.
217	16806112	Neuroprotective role of Z-ligustilide against forebrain ischemic injury in ICR mice. Kuang, X. et al. 2006. Brain Res. 1102: 145-53.

218	16849563	The Human Orthologue of Drosophila Ecdysoneless Protein Interacts with p53 and Regulates Its Function Zhang, Y. et al. 2006. Cancer Res. 66: 7167-7175.
219	16865289	DHMEQ, a novel NF-kappaB inhibitor, induces apoptosis and cell-cycle arrest in human hepatoma cells. Nishimura, D. et al. 2006. Int. J. Oncol. 29: 713-9.
220	16866858	Expression of Egr-1 gene and its correlation with the oncogene proteins in non-irradiated and irradiated esophageal squamous cell carcinoma. Wu, MY. et al. 2006. Dis. Esophagus. 19: 267-72.
221	16870742	Mitochondrial translocation of p53 mediates release of cytochrome c and hippocampal CA1 neuronal death after transient global cerebral ischemia in rats Endo, H. et al. 2006. J Neurosci. 26(30): 7974-7983.
222	16888648	Cell Death Differ. 511-23.
223	16964588	The effect of chelerythrine on cell growth, apoptosis, and cell cycle in human normal and cancer cells in comparison with sanguinarine. Malíková, J. et al. 2006. Cell Biol. Toxicol. 22: 439-453.
224	16998810	SIRT1 interacts with p73 and suppresses p73-dependent transcriptional activity. Dai, JM. et al. 2007. J. Cell. Physiol. 210: 161-6.
225	17055086	CD133^{+} melanoma subpopulation acquired resistance to caffeic acid phenethyl ester-induced apoptosis is attributed to the elevated expression of ABCB5: significance for melanoma treatment. El-Khattouti, A. et al. 2015. Cancer Lett. 357: 83-104.
226	17079469	A novel anticancer gold(III) dithiocarbamate compound inhibits the activity of a purified 20S proteasome and 26S proteasome in human breast cancer cell cultures and xenografts Milacic, V. et al. 2006. Cancer Res. 66(21): 10478-10486.
227	17091766	Apoptotic markers p53, Bcl-2 and Bax in primary lung cancer. Porebska, I. et al. In Vivo. 20: 599-604.
228	17093135	The tumor proteasome is a primary target for the natural anticancer compound Withaferin A isolated from "Indian winter cherry". Yang, H. et al. 2007. Mol. Pharmacol. 71: 426-37.
229	17094488	Resistance to cisplatin and adriamycin is associated with the inhibition of glutathione efflux in MCF-7-derived cells. Osbild, S. et al. Anticancer Res. 26: 3595-600.
230	17118559	Immobilized alpha2,6-linked sialic acid suppresses caspase-3 activation during anti-IgM antibody-induced apoptosis in Ramos cells. Azuma, Y. et al. 2007. Biochim. Biophys. Acta. 1770: 279-85.
231	17123509	Skeletal unloading induces osteoblast apoptosis and targets alpha5beta1-PI3K-Bcl-2 signaling in rat bone. Dufour, C. et al. 2007. Exp. Cell Res. 313: 394-403.
232	17133357	Development of the terminally differentiated state sensitizes epiphyseal chondrocytes to apoptosis through caspase-3 activation. Pucci, B. et al. 2007. J. Cell. Physiol. 210: 609-15.
233	17136495	Increased ROS generation and p53 activation in alpha-lipoic acid-induced apoptosis of hepatoma cells. Simbula, G. et al. 2007. Apoptosis. 12: 113-23.
234	17141999	Age-related hearing loss in CD/1 mice is associated to ROS formation and HIF target proteins up-regulation in the cochlea. Riva, C. et al. 2007. Exp. Gerontol. 42: 327-336.
235	17145862	Lithium treatment prevents neurocognitive deficit resulting from cranial irradiation Yazlovitskaya, EM. et al. 2006. Cancer Res. 66(23): 11179-11186.
236	17164408	Activation of the IGF1 system characterizes cholangiocyte survival during progression of primary biliary cirrhosis. Onori, P. et al. 2006. J Histochem Cytochem. 55: 327-334.
237	17194518	Expression changes of apoptotic-related proteins in nerve tissues of rats treated with allyl chloride. Wang, QS. et al. 2007. Toxicology. 231: 58-67.
238	17216583	The functional haplotype of peptidylarginine deiminase IV (S55G, A82V and A112G) associated with susceptibility to rheumatoid arthritis dominates apoptosis of acute T leukemia Jurkat cells. Hung, H.C. et al. 2007. Apoptosis. 12: 475-487.
239	17291488	Matrine upregulates the cell cycle protein E2F-1 and triggers apoptosis via the mitochondrial pathway in K562 cells. Jiang, H. et al. 2007. Eur. J. Pharmacol. 559: 98-108.
240	17299396	Recombinant human erythropoietin alpha modulates the effects of radiotherapy on colorectal cancer microvessels. Ceelen, W. et al. 2007. Br. J. Cancer. 96: 692-700.
241	17364023	Reactivation of the p53 pathway as a treatment modality for KSHV-induced lymphomas Sarek, G. et al. 2007. J. Clin. Invest. 117: 1019-1028.
242	17376698	Time-dependent onset of Interferon-alpha2b-induced apoptosis in isolated hepatocytes from preneoplastic rat livers Alvarez, M.D. et al. 2007. Cytokine. 36: 245-253.
243	17384938	Chronic methadone treatment and repeated withdrawal impair cognition and increase the expression of apoptosis-related proteins in mouse brain Tramullas, M. et al. 2007. Psychopharmacology (Berl). 193: 107-120.
244	17431754	High Bcl-2/Bax ratio in Walker tumor cells protects mitochondria but does not prevent H2O2-induced apoptosis via calcineurin pathways. Zecchin, KG. et al. 2007. J. Bioenerg. Biomembr. 39: 186-94.
245	17434727	Sulindac treatment in hereditary non-polyposis colorectal cancer. Eur. J. Cancer. 43: 1251-6.
246	17461764	Specific apoptosis induction in human papillomavirus-positive cervical carcinoma cells by photodynamic antisense regulation. Yamayoshi, A. et al. 2007. Oligonucleotides. 17: 66-79.
247	17470478	Free fatty acids sensitise hepatocytes to TRAIL mediated cytotoxicity Malhi, H. et al. 2007. Gut. 56(8): 1124-1131.
248	17483343	A novel prodrug of the green tea polyphenol (-)-epigallocatechin-3-gallate as a potential anticancer agent. Landis-Piwowar, KR. et al. 2007. Cancer Res. 67: 4303-4310.
249	17483459	Two molecular pathways initiate mitochondria-dependent dopaminergic neurodegeneration in experimental Parkinson's disease Perier, C. et al. 2007. Proc. Natl. Acad. Sci. USA. 104(19): 8161-8166.
250	17486276	Expression of Nkx3.1 enhances 17beta-estradiol anti-tumor action in PC3 human prostate cancer cells. Wang, P. et al. 2007. Asian J. Androl. 9: 353-60.
251	17492505	Expression of Bcl-2, Bax and Caspase-3 in nerve tissues of rats chronically exposed to 2,5-hexanedione. Cui, N. et al. 2007. Neurochem. Res. 32: 1566-72.
252	17504094	The role of Bax/Bcl-2 and pro-caspase peptides in hypoxia/reperfusion-dependent regulation of MAPK(ERK): discordant proteomic effect of MAPK(p38). Haddad, JJ. et al. 2007. Protein Pept. Lett. 14: 361-71.
253	17541980	Pristimerin induces apoptosis by targeting the proteasome in prostate cancer cells. Yang, H. et al. 2008. J. Cell. Biochem. 103: 234-44.
254	17565738	Camptothecin-induced apoptosis is enhanced by Myc and involves PKCdelta signaling. Albihn, A. et al. 2007. Int. J. Cancer. 121: 1821-9.
255	17673183	Protection by doxycycline against doxorubicin-induced oxidative stress and apoptosis in mouse testes. Yeh, Y.C. et al. 2007. Biochem Pharmacol. 74: 969-980.
256	17674369	Altering DNA base excision repair: use of nuclear and mitochondrial-targeted N-methylpurine DNA glycosylase to sensitize astroglia to chemotherapeutic agents. Harrison, JF. et al. 2007. Glia. 55: 1416-1425.
257	17679146	Role of peroxidases, thiols and Bak/Bax in tumor cell susceptibility to Cu[DEDTC]2 Viola-Rhenals, M. et al. 2007. Biochem Pharmacol. 74(6): 841-850.
258	17686764	Tumor necrosis factor-related apoptosis-inducing ligand activates a lysosomal pathway of apoptosis that is regulated by Bcl-2 proteins. Werneburg, NW. et al. 2007. J. Biol. Chem. 282: 28960-70.
259	17710536	Piracetam improves cognitive deficits caused by chronic cerebral hypoperfusion in rats. He, Z. et al. 2008. Cell. Mol. Neurobiol. 28: 613-27.
260	17825529	Diabetes enhances cell proliferation but not Bax/Bcl-2-mediated apoptosis during oral oncogenesis. Vairaktaris, E. et al. 2008. Int J Oral Maxillofac Surg. 37: 60-5.
261	17828456	Celecoxib after the onset of reperfusion reduces apoptosis in the amygdala. Kaloustian, S. et al. 2007. Apoptosis. 12: 1945-51.
262	17855663	Ursolic acid inhibits STAT3 activation pathway leading to suppression of proliferation and chemosensitization of human multiple myeloma cells Pathak, A.K. et al. 2007. Mol Cancer Res. 5: 943-955.
263	17881166	Cimicifuga foetida extract inhibits proliferation of hepatocellular cells via induction of cell cycle arrest and apoptosis. Tian, Z. et al. 2007. J Ethnopharmacol. 114: 227-33.
264	17881359	p53 is a key molecular target of ursodeoxycholic acid in regulating apoptosis Amaral, J.D. et al. 2007. J Biol Chem. 282: 34250-34259.
265	17885668	Bax-inhibiting peptide protects cells from polyglutamine toxicity caused by Ku70 acetylation. Li, Y. et al. 2007. Cell Death Differ. 14: 2058-2067.
266	17893235	Alpha-lipoic acid promotes the growth of rat hepatic pre-neoplastic lesions in the choline-deficient model. Perra, A. et al. 2008. Carcinogenesis. 29: 161-8.
267	17893875	Erythropoietin treatment of human ovarian cancer cells results in enhanced signaling and a paclitaxel-resistant phenotype. Solar, P. et al. 2008. Int. J. Cancer. 122: 281-8.
268	17899381	Sequential phases of Ca2+ alterations in pre-apoptotic cells. Cerella, C. et al. 2007. Apoptosis. 12: 2207-19.
269	17982703	Evaluation of copper-dependent proteasome-inhibitory and apoptosis-inducing activities of novel pyrrolidine dithiocarbamate analogues. Yu, Z. et al. 2007. Int. J. Mol. Med. 20: 919-25.
270	17999188	Early carcinogenic events in HNPCC adenomas: differences with sporadic adenomas. Dig. Dis. Sci. 53: 1660-8.
271	18019411	Modulation of Bax expression in physiological and pathological human placentas throughout pregnancy. Cobellis, L. et al. In Vivo. 21: 777-83.
272	18023291	Thrombin peptide TP508 prevents nitric oxide mediated apoptosis in chondrocytes in the endochondral developmental pathway. Zhong, M. et al. 2008. Biochim. Biophys. Acta. 1783: 12-22.
273	18027855	Fibroblast nemosis arrests growth and induces differentiation of human leukemia cells. Kankuri, E. et al. 2008. Int. J. Cancer. 122: 1243-1252.
274	18042550	DJ-1 decreases Bax expression through repressing p53 transcriptional activity. Fan, J. et al. 2008. J. Biol. Chem. 283: 4022-4030.
275	18048012	Expression of survivin and p16(INK4a)/Cdk6/pRB proteins and induction of apoptosis in response to radiation and cisplatin in meningioma cells. Al-Khalaf, HH. et al. 2008. Brain Res. 1188: 25-34.
276	18070986	Wogonin preferentially kills malignant lymphocytes and suppresses T-cell tumor growth by inducing PLCgamma1- and Ca2+-dependent apoptosis. Baumann, S. et al. 2008. Blood. 111: 2354-2363.
277	18081943	Gamma-tocotrienol-induced apoptosis in human gastric cancer SGC-7901 cells is associated with a suppression in mitogen-activated protein kinase signalling. Sun, W. et al. 2008. Br. J. Nutr. 99: 1247-1254.
278	18084329	Suppression of cFLIP is sufficient to sensitize human melanoma cells to TRAIL- and CD95L-mediated apoptosis. Geserick, P. et al. 2007. Oncogene. 27: 3211-3220.
279	18097588	Differential involvement of mitochondria during ursolic acid-induced apoptotic process in HaCaT and M4Beu cells. Duval, RE. et al. 2008. Oncol. Rep. 19: 145-9.
280	18166137	Antitumor activity and mechanisms of action of total glycosides from aerial part of Cimicifuga dahurica targeted against hepatoma. Tian, Z. et al. 2007. BMC Cancer. 7: 237.
281	18191419	Activity of novel Cdc25 inhibitors and preliminary evaluation of their potentiation of chemotherapeutic drugs in human breast cancer cells. Brault, L. et al. 2008. Life Sci. 82: 315-23.
282	18206650	Levels changes of blood leukocytes and intracellular signalling pathways in COPD patients with respect to smoking attitude. Rumora, L. et al. 2008. Clin. Biochem. 41: 387-94.
283	18296448	Implantation-associated gene-1 (Iag-1): a novel gene involved in the early process of embryonic implantation in rat. Xia, HF. et al. 2008. Hum. Reprod. 23: 1581-93.
284	18300387	Inhibition of proteasome activity by the dietary flavonoid apigenin is associated with growth inhibition in cultured breast cancer cells and xenografts. Chen, D. et al. 2007. Breast Cancer Res. 9: R80.
285	18304598	Disulfiram promotes the conversion of carcinogenic cadmium to a proteasome inhibitor with pro-apoptotic activity in human cancer cells. Li, L. et al. 2008. Toxicol. Appl. Pharmacol. 229: 206-14.
286	18317946	Premature senescence of human endothelial cells induced by inhibition of glutaminase. Unterluggauer, H. et al. 2008. Biogerontology. 9: 247-59.
287	18323654	Stabilization of p53 is involved in quercetin-induced cell cycle arrest and apoptosis in HepG2 cells. Tanigawa, S. et al. 2008. Biosci. Biotechnol. Biochem. 72: 797-804.
288	18339323	Sumoylation is critical for DJ-1 to repress p53 transcriptional activity. Fan, J. et al. 2008. FEBS Lett. 582: 1151-1156.
289	18363432	Radiation-induced hyposuppression of the hypothalamic-pituitary-adrenal axis is associated with alterations of hippocampal corticosteroid receptor expression. Velicković, N. et al. 2008. Radiat. Res. 169: 397-407.
290	18370874	Cyclin-dependent kinase inhibitor roscovitine induces cell cycle arrest and apoptosis in rabbit retinal pigment epithelial cells. Wu, PC. et al. 2008. J Ocul Pharmacol Ther. 24: 25-33.
291	18378961	Transforming growth factor-beta prevents osteoblast apoptosis induced by skeletal unloading via PI3K/Akt, Bcl-2, and phospho-Bad signaling. Dufour C, et al. 2008. Am. J. Physiol. 294: 794-801.
292	18385271	Antiapoptotic properties of erythropoiesis-stimulating proteins in models of cisplatin-induced acute kidney injury. Salahudeen, AK. et al. 2008. Am. J. Physiol. Renal Physiol. 294: F1354-65.
293	18501397	Pyrrolidine dithiocarbamate-zinc(II) and -copper(II) complexes induce apoptosis in tumor cells by inhibiting the proteasomal activity. Milacic, V. et al. 2008. Toxicol. Appl. Pharmacol. 231: 24-33.
294	18506996	Cellular response to irinotecan in colon cancer cell lines showing differential response to 5-fluorouracil. Haug, K. et al. Anticancer Res. 28: 583-92.
295	18509476	A bacterial cytotoxin identifies the RhoA exchange factor Net1 as a key effector in the response to DNA damage. PLoS ONE. 3: e2254.
296	18510170	Dihydroartemisinin induces apoptosis in human leukemia cells HL60 via downregulation of transferrin receptor expression. Anticancer Drugs. 19: 247-55.
297	18564172	Effects of cyclooxygenase-2 non-selective and selective inhibitors on proliferation inhibition and apoptosis induction of esophageal squamous carcinoma cells. Li, P. et al. 2009. Diseases of the esophagus : official journal of the International Society for Diseases of the Esophagus / I.S.D.E. 22: 21-31.
298	18627254	Disruption of Bax protein prevents neuronal cell death but produces cognitive impairment in mice following traumatic brain injury. Tehranian, R. et al. 2008. J. Neurotrauma. 25: 755-67.
299	18632596	Ginkgo biloba extract 761 reduces doxorubicin-induced apoptotic damage in rat hearts and neonatal cardiomyocytes. [Epub ahead of print] Liu, T.J. et al. 2008. Cardiovasc Res.
300	18636149	The A3 adenosine receptor agonist CF102 induces apoptosis of hepatocellular carcinoma via de-regulation of the Wnt and NF-kappaB signal transduction pathways Bar-Yehuda, S. et al. 2008. Int J Oncol. 33: 287-295.
301	18683188	Humic acid induces G1 phase arrest and apoptosis in cultured vascular smooth muscle cells. Hseu, YC. et al. 2009. Environ. Toxicol. 24: 243-258.
302	18767070	Apple polyphenol phloretin potentiates the anticancer actions of paclitaxel through induction of apoptosis in human hep G2 cells. Yang, KC. et al. 2009. Mol. Carcinog. 48: 420-431.
303	18794115	Curcumin inhibits the proteasome activity in human colon cancer cells in vitro and in vivo Milacic V, et al. 2008. Cancer Res. 68(18): 7283-7292.
304	18798056	Susceptibility for NSAIDs-induced apoptosis correlates to p53 gene status in gastric cancer cells. Zhang, YJ. et al. 2008. Cancer Invest. 26: 868-77.
305	18800351	Diallyl trisulfide selectively causes Bax- and Bak-mediated apoptosis in human lung cancer cells. Xiao, D. et al. 2009. Environ. Mol. Mutagen. 50: 201-212.
306	18937880	Dysregulation of cell death machinery in the prefrontal cortex of human alcoholics. Johansson, S. et al. 2009. Int. J. Neuropsychopharmacol. 12: 109-115.
307	18937971	TP53, BCL-2 and BAX analysis in 199 ovarian cancer patients treated with taxane-platinum regimens. Ziółkowska-Seta, I. et al. Gynecol. Oncol. 112: 179-84.
308	18949390	Induction of tumor cell apoptosis by taurine Schiff base copper complex is associated with the inhibition of proteasomal activity. Zhang, X. et al. 2008. Int. J. Mol. Med. 22: 677-82.
309	19020042	Selective activation of p38 mitogen-activated protein kinase in dopaminergic neurons of substantia nigra leads to nuclear translocation of p53 in 1-methyl-4-phenyl-1,2,3,6-tetrahydropyridine-treated mice. Karunakaran, S. et al. 2008. J. Neurosci. 28: 12500-12509.
310	19082491	Anti-proliferative and apoptosis-inducible activity of Sarcodonin G from Sarcodon scabrosus in HeLa cells. Dong, M. et al. 2009. Int. J. Oncol. 34: 201-7.
311	19097688	Andrographolide enhances 5-fluorouracil-induced apoptosis via caspase-8-dependent mitochondrial pathway involving p53 participation in hepatocellular carcinoma (SMMC-7721) cells. Yang, L. et al. 2009. Cancer Lett. 276: 180-8.
312	19115221	Thyroid hormone receptor ligands induce regression of rat preneoplastic liver lesions causing their reversion to a differentiated phenotype. Perra, A. et al. 2009. Hepatology. 49: 1287-96.
313	19121919	Inhibition of proliferation and induction of apoptosis by gamma-tocotrienol in human colon carcinoma HT-29 cells. Xu, WL. et al. 2009. Nutrition. 25: 555-66.
314	19123483	In vitro and in vivo study of phloretin-induced apoptosis in human liver cancer cells involving inhibition of type II glucose transporter. Wu, CH. et al. 2009. Int. J. Cancer. 124: 2210-2219.
315	19130057	Activation of ER stress and inhibition of EGFR N-glycosylation by tunicamycin enhances susceptibility of human non-small cell lung cancer cells to erlotinib. Ling, YH. et al. 2009. Cancer Chemother. Pharmacol. 64: 539-48.
316	19130237	Sodium butyrate sensitizes human colon adenocarcinoma COLO 205 cells to both intrinsic and TNF-alpha-dependent extrinsic apoptosis. Pajak, B. et al. 2009. Apoptosis. 14: 203-217.
317	19165796	Different apoptosis modalities in periprosthetic membranes. J Biomed Mater Res A. 92: 175-84.
318	19165859	Shikonin exerts antitumor activity via proteasome inhibition and cell death induction in vitro and in vivo. Yang, H. et al. 2009. Int. J. Cancer. 124: 2450-9.
319	19208092	Arsenic-trioxide-induced apoptosis of chronic lymphocytic leukemia cells. Bairey O, Vanichkin A, Shpilberg O. et al.
320	19247369	Hdm2 is a ubiquitin ligase of Ku70-Akt promotes cell survival by inhibiting Hdm2-dependent Ku70 destabilization. Gama, V. et al. 2009. Cell Death Differ. 16: 758-769.
321	19264955	Induction of p53 contributes to apoptosis of HCT-116 human colon cancer cells induced by the dietary compound fisetin. Lim, DY. et al. 2009. Am. J. Physiol. Gastrointest. Liver Physiol. 296: G1060-8.
322	19266349	Nkx3.1 and p27(KIP1) cooperate in proliferation inhibition and apoptosis induction in human androgen-independent prostate cancer cells. Wang, P. et al. 2009. Cancer Invest. 27: 369-75.
323	19278624	Hydrogen peroxide-induced Akt phosphorylation regulates Bax activation. Sadidi, M. et al. 2009. Biochimie. 91: 577-85.
324	19291793	Pml and TAp73 interacting at nuclear body mediate imatinib-induced p53-independent apoptosis of chronic myeloid leukemia cells. Liu, JH. et al. 2009. Int. J. Cancer. 125: 71-7.
325	19337512	The proteasome is a molecular target of environmental toxic organotins. Shi, G. et al. 2009. Environ. Health Perspect. 117: 379-86.
326	19395858	ERK activation increases nitroprusside induced apoptosis in human melanoma cells irrespective of p53 status: role of superoxide dismutases. Gomez-Sarosi, LA. et al. 2009. Cancer Biol. Ther. 8: 1173-82.
327	19418225	Apoptosis-inducing factor plays a critical role in caspase-independent, pyknotic cell death in hydrogen peroxide-exposed cells. Son, YO. et al. 2009. Apoptosis. 14: 796-808.
328	19428345	Enhancement of esculetin on arsenic trioxide-provoked apoptosis in human leukemia U937 cells. Lin, TH. et al. 2009. Chem. Biol. Interact. 180: 61-68.
329	19429377	MAPK activation is necessary to the apoptotic death of KB cells induced by the essential oil isolated from Artemisia iwayomogi. Cha, JD. et al. 2009. J Ethnopharmacol. 123: 308-314.
330	19476552	Modulation of stress proteins and apoptotic regulators in the anoxia tolerant turtle brain. Kesaraju, S. et al. 2009. J. Neurochem. 109: 1413-26.
331	19491899	High Ki67, Bax, and thymidylate synthase expression well correlates with response to chemoradiation therapy in locally advanced rectal cancers: proposal of a logistic model for prediction. Kikuchi, M. et al. 2009. Br J Cancer. 101: 116-23.
332	19502098	The rotamase Pin1 is up-regulated, hypophosphorylated and required for cell cycle progression in head and neck squamous cell carcinomas. Wiegand, S. et al. 2009. Oral Oncol. 45: e140-e149.
333	19528817	Chronic pretreatment with celecoxib reduces infarct size. Lada-Moldovan, L. et al. 2009. J. Cardiovasc. Pharmacol. 54: 31-7.
334	19540915	Pyridoxamine protects intestinal epithelium from ionizing radiation-induced apoptosis. Thotala, D. et al. 2009. Free Radic. Biol. Med. 47: 779-85.
335	19657228	The C-terminus of interferon gamma receptor beta chain (IFNgammaR2) has antiapoptotic activity as a Bax inhibitor. Cancer Biol. Ther. 8: 1771-86.
336	19665148	Heme oxygenase-1 prevents hyperthyroidism induced hepatic damage via an antioxidant and antiapoptotic pathway. Giriş, M. et al. 2010. J. Surg. Res. 164: 266-275.
337	19669692	Chronic social isolation is related to both upregulation of plasticity genes and initiation of proapoptotic signaling in Wistar rat hippocampus. Djordjevic A et al. 2009. J Neural Transm. 116: 1579-1589.
338	19685289	Combination treatment with resveratrol and sulforaphane induces apoptosis in human U251 glioma cells. Jiang H, Shang X, Wu H, Huang G, Wang Y, Al-Holou S, Gautam SC, Chopp M. et al.
339	19696786	Role of FK506-binding protein 51 in the control of apoptosis of irradiated melanoma cells. Romano, S. et al. 2010. Cell Death Differ. 17: 145-157.
340	19699182	Proteasome inhibitors prevent cisplatin-induced mitochondrial release of apoptosis-inducing factor and markedly ameliorate cisplatin nephrotoxicity. Liu, L. et al. 2010. Biochem. Pharmacol. 79: 137-146.
341	19705353	Coumarin A/AA induces apoptosis-like cell death in HeLa cells mediated by the release of apoptosis-inducing factor. Alvarez-Delgado, C. et al. 2009 Jul-Aug. J. Biochem. Mol. Toxicol. 23: 263-272.
342	19709099	Suberythemal ultraviolet B radiation alters the expression of cell cycle-related proteins in the epidermis of human subjects without leading to photoprotection. Narbutt, J. et al. 2009. Br. J. Dermatol. 161: 890-6.
343	19740103	Interferon-gamma bolsters CD95/Fas-mediated apoptosis of astroglioma cells. FEBS J. 276: 5920-35.
344	19782136	Long-term incubation with beta-amyloid peptides impairs endothelium-dependent vasodilatation in isolated rat basilar artery. Chisari, M. et al. 2009. Pharmacol. Res. -.
345	19790055	Induction of an antiinflammatory effect and prevention of cartilage damage in rat knee osteoarthritis by CF101 treatment. Bar-Yehuda, S. et al. 2009. Arthritis Rheum. 60: 3061-71.
346	19809836	Tumor cellular proteasome inhibition and growth suppression by 8-hydroxyquinoline and clioquinol requires their capabilities to bind copper and transport copper into cells. Zhai, S. et al. 2010. J. Biol. Inorg. Chem. 15: 259-69.
347	19815400	Isorhamnetin protects rat ventricular myocytes from ischemia and reperfusion injury. Zhang, N. et al. 2011. Exp. Toxicol. Pathol. 63: 33-8.
348	19885572	Novel 8-hydroxylquinoline analogs induce copper-dependent proteasome inhibition and cell death in human breast cancer cells. Milacic, V. et al. 2009. Int. J. Oncol. 35: 1481-91.
349	19892564	Tumor necrosis factor alpha induced by Trypanosoma cruzi infection mediates inflammation and cell death in the liver of infected mice. Ronco MT et al. 2010. Cytokine. 49: 64-72.
350	19896474	Enhanced cerebral ischemic lesions after two-vein occlusion in diabetic rats. Wajima, D. et al. 2010. Brain Res. 1309: 126-35.
351	19903580	Triptolide simultaneously induces reactive oxygen species, inhibits NF-kappaB activity and sensitizes 5-fluorouracil in colorectal cancer cell lines. Cancer Lett. 291: 200-8.
352	19946756	Mitochondrial cholesterol transporter, StAR, inhibits human THP-1 monocyte-derived macrophage apoptosis. Bai, Q. et al. 2010. Lipids. 45: 29-36.
353	19962231	Antitumor activity of novel fluoro-substituted (-)-epigallocatechin-3-gallate analogs. Yang, H. et al. 2010. Cancer Lett. 292: 48-53.
354	19995710	Dexamethasone effects on Bax expression in the mouse testicular germ cells. Mahmoud, H. et al. 2009. Folia Histochem. Cytobiol. 47: 237-41.
355	20025076	Curcumin inhibits the Sonic Hedgehog signaling pathway and triggers apoptosis in medulloblastoma cells. Elamin, MH. et al. 2010. Mol. Carcinog. 49: 302-314.
356	20036724	Ganoderma lucidum induced apoptosis in NB4 human leukemia cells: involvement of Akt and Erk. Calviño, E. et al. 2010. J Ethnopharmacol. 128: 71-78.
357	20041963	Potential role of CXCL10 in the induction of cell injury and mitochondrial dysfunction. Singh, L. et al. 2010. Int J Exp Pathol. 91: 210-23.
358	20054652	AChE deficiency or inhibition decreases apoptosis and p53 expression and protects renal function after ischemia/reperfusion. Ye, W. et al. 2010. Apoptosis. 15: 474-487.
359	20151116	Antiapoptotic effect of angiotensin-II type-1 receptor blockade in renal tubular cells of hyperoxaluric rats. Tunçdemir, M. et al. 2010. Urol. Res. 38: 71-80.
360	20164374	Hyperglycemia induces apoptosis in rat liver through the increase of hydroxyl radical: new insights into the insulin effect. Francés, DE. et al. 2010. J. Endocrinol. 205: 187-200.
361	20200969	TNF-alpha's effects on proliferation and apoptosis in human mesenchymal stem cells depend on RUNX2 expression. Ghali, O. et al. 2010. Journal of bone and mineral research : the official journal of the American Society for Bone and Mineral Research. 25: 1616-26.
362	20224069	Nrf2-regulated PPAR{gamma} expression is critical to protection against acute lung injury in mice. Cho, HY. et al. 2010. American journal of respiratory and critical care medicine. 182: 170-82.
363	20299527	Inhibition of mTORC1 signaling reduces tumor growth but does not prevent cancer progression in a mouse model of thyroid cancer. Guigon, CJ. et al. 2010. Carcinogenesis. 31: 1284-1291.
364	20304020	Differential effect of platelet activating factor on 1-methyl-4-phenylpyridinium-induced cell death through regulation of apoptosis-related protein activation. Ha, SW. et al. 2010 May-Jun. Neurochem. Int. 56: 819-828.
365	20354190	Noscapine, a benzylisoquinoline alkaloid, sensitizes leukemic cells to chemotherapeutic agents and cytokines by modulating the NF-kappaB signaling pathway. Sung B et al. 2010. Cancer Res. 70: 3259-3268.
366	20371723	Garcinol potentiates TRAIL-induced apoptosis through modulation of death receptors and antiapoptotic proteins. Prasad S et al. 2010. Mol Cancer Ther. 9: 856-868.
367	20403207	The conformation change of Bcl-2 is involved in arsenic trioxide-induced apoptosis and inhibition of proliferation in SGC7901 human gastric cancer cells. Zheng, Y. et al. 2010. World journal of surgical oncology. 8: 31.
368	20410588	Granulocyte colony-stimulating factor treatment prevents cognitive impairment following status epilepticus in rats. Zhang, Y. et al. 2010. Biol. Pharm. Bull. 33: 572-9.
369	20421947	Cdh11 acts as a tumor suppressor in a murine retinoblastoma model by facilitating tumor cell death. Marchong, MN. et al. 2010. PLoS Genet. 6: -.
370	20427144	Expression of luteal estrogen receptor, interleukin-1, and apoptosis-associated genes after PGF2alpha administration in rabbits at different stages of pseudopregnancy. Maranesi, M. et al. 2010. Domest. Anim. Endocrinol. 39: 116-130.
371	20461754	MDM2 gene amplification in colorectal cancer is associated with disease progression at the primary site, but inversely correlated with distant metastasis. Sugano, N. et al. 2010. Genes Chromosomes Cancer. 49: 620-9.
372	20504359	Anti-proliferative effect of LXR agonist T0901317 in ovarian carcinoma cells. Rough, JJ. et al. 2010. J Ovarian Res. 3: -.
373	20511721	Hypertonicity-induced mitochondrial membrane permeability in renal medullary interstitial cells: protective role of osmolytes. Zhang, L. et al. 2010. Cell. Physiol. Biochem. 25: 753-60.
374	20525171	Downregulation of SPARC expression decreases gastric cancer cellular invasion and survival. Yin, J. et al. 2010. J. Exp. Clin. Cancer Res. 29: -.
375	20537511	Eugenol restricts DMBA croton oil induced skin carcinogenesis in mice: downregulation of c-Myc and H-ras, and activation of p53 dependent apoptotic pathway. Pal, D. et al. 2010. J. Dermatol. Sci. 59: 31-39.
376	20543557	The polycomb group protein BMI1 is a transcriptional target of HDAC inhibitors. Bommi, PV. et al. 2010. Cell Cycle. 9: -.
377	20554630	Apoptosis contributes to testicular toxicity induced by two isomers of bromopropanes. Xin, QQ. et al. 2010. Toxicol Ind Health. 26: 513-524.
378	20556587	Aortic cell apoptosis in rat primary aldosteronism model. Yan, Y. et al. 2010. J. Huazhong Univ. Sci. Technol. Med. Sci. 30: 385-90.
379	20568239	Cytotoxic action of Ganoderma lucidum on interleukin-3 dependent lymphoma DA-1 cells: involvement of apoptosis proteins. Calviño, E. et al. 2011. Phytother Res. 25: 25-32.
380	20599452	Pro-apoptotic effect of methylguanidine on hydrogen peroxide-treated rat glioma cell line. Marzocco S. et al. 2010. Neurochem Int. 57(5): 518-524.
381	20623537	Chronic social isolation suppresses proplastic response and promotes proapoptotic signalling in prefrontal cortex of Wistar rats. Djordjevic A et al. 2010. J Neurosci Res. 88: 2524-2533.
382	20663862	Overexpression of CD133 promotes drug resistance in C6 glioma cells. Angelastro, JM. et al. 2010. Mol. Cancer Res. 8: 1105-15.
383	20691257	Arecoline, a major alkaloid of the areca nut, causes neurotoxicity through enhancement of oxidative stress and suppression of the antioxidant protective system. Shih, YT. et al. 2010. Free Radic. Biol. Med. 49: 1471-1479.
384	20709028	Doxorubicin-induced cell death requires cathepsin B in HeLa cells. Bien, S. et al. 2010. Biochem. Pharmacol. 80: 1466-1477.
385	20724592	Prostate apoptosis response-4 is expressed in normal cholangiocytes, is down-regulated in human cholangiocarcinoma, and promotes apoptosis of neoplastic cholangiocytes when induced pharmacologically. Franchitto, A. et al. 2010. Am. J. Pathol. 177: 1779-90.
386	20833159	Brassinosteroids cause cell cycle arrest and apoptosis of human breast cancer cells. Steigerová, J. et al. 2010. Chem. Biol. Interact. 188: 487-96.
387	20837473	Gossypol induces death receptor-5 through activation of the ROS-ERK-CHOP pathway and sensitizes colon cancer cells to TRAIL. Sung B et al. 2010. J Biol Chem. 285: 35418-35427
388	20874679	Antiapoptotic effect of novel compound from Herba leonuri-leonurine (SCM-198): a mechanism through inhibition of mitochondria dysfunction in H9c2 cells. Liu XH et al. 2010. Curr Pharm Biotechnol. 11: 895-905.
389	20933054	Modulation of Bcl-2-related protein expression in pancreatic beta cells by pro-inflammatory cytokines and its dependence on the antioxidative defense status. Mehmeti I, Lenzen S, Lortz S. et al. 2011. Molecular and cellular endocrinology. 332: 88-96.
390	20939431	Expression of the major fas family and Bcl-2 family of proteins in epithelial ovarian cancer (EOC) and their correlation to chemotherapeutic response and outcome. Oncol. Res. 18: 549-59.
391	20950152	Growth inhibition effect of β-catenin small interfering RNA-mediated gene silencing on human colon carcinoma HT-29 cells. Xu, WL. et al. 2010. Cancer Biother. Radiopharm. 25: 529-537.
392	21035514	7-ketocholesterol induces apoptosis in differentiated PC12 cells via reactive oxygen species-dependent activation of NF-κB and Akt pathways. Jang, ER. et al. 2011. Neurochem. Int. 58: 52-59.
393	21035945	A novel dithiocarbamate analogue with potentially decreased ALDH inhibition has copper-dependent proteasome-inhibitory and apoptosis-inducing activity in human breast cancer cells. Wang, F. et al. 2011. Cancer Lett. 300: 87-95.
394	21078664	Nimbolide sensitizes human colon cancer cells to TRAIL through reactive oxygen species- and ERK-dependent up-regulation of death receptors, p53, and Bax Gupta SC, Reuter S, Phromnoi K, Park B, Hema PS, Nair M, Aggarwal BB. et al. 2011. J Biol Chem. 286: 1134-1146.
395	21104071	Inorganic phosphate induces mammalian growth plate chondrocyte apoptosis in a mitochondrial pathway involving nitric oxide and JNK MAP kinase. Zhong, M. et al. 2011. Calcif. Tissue Int. 88: 96-108.
396	21127198	ROS and CHOP are critical for dibenzylideneacetone to sensitize tumor cells to TRAIL through induction of death receptors and downregulation of cell survival proteins. Prasad S et al. 2011. Cancer Res. 71: 538-549.
397	21128225	Inhibition of signal transducer and activator of transcription 5 by the inhibitor of janus kinases stimulates dormant human leukemia CD34+ /CD38- cells and sensitizes them to antileukemia agents. Yang J, Nishioka C, Kojima S, Takeuchi A, Phillip Koeffler H, Yokoyama A. et al.
398	21156789	Ursolic acid, a pentacyclin triterpene, potentiates TRAIL-induced apoptosis through p53-independent up-regulation of death receptors: evidence for the role of reactive oxygen species and JNK. Prasad S et al. 2011. J Biol Chem. 286: 5546-5557.
399	21223552	A human ribonuclease induces apoptosis associated with p21WAF1/CIP1 induction and JNK inactivation. Castro, J. et al. 2011. BMC Cancer. 11: -.
400	21224352	Amiloride modulates alternative splicing in leukemic cells and resensitizes Bcr-AblT315I mutant cells to imatinib. Chang WH et al. 2011. Cancer Res. 71: 383-392.
401	21241688	Apoptosis induced by a new flavonoid in human hepatoma HepG2 cells involves reactive oxygen species-mediated mitochondrial dysfunction and MAPK activation. Liu, H. et al. 2011. European journal of pharmacology. 654: 209-16.
402	21246688	Flexible gold-catalyzed regioselective oxidative difunctionalization of unactivated alkenes. de Haro, T. et al. 2011. Angew. Chem. Int. Ed. Engl. 50: 906-910.
403	21273574	Inhibition of tumor cellular proteasome activity by triptolide extracted from the Chinese medicinal plant 'thunder god vine'. Lu, L. et al. 2011. Anticancer Res. 31: 1-10.
404	21284050	Role of DNA methyltransferase 1 in pharyngeal cancer related to treatment resistance Miao-Fen,
405	21310776	Testicular apoptosis and histopathological changes induced by a 2.45 GHz electromagnetic field. Saygin, M. et al. 2011. Toxicol Ind Health. 27: 455-463.
406	21383988	Guggulsterone targets smokeless tobacco induced PI3K/Akt pathway in head and neck cancer cells. Macha MA, Matta A, Chauhan SS, Siu KW, Ralhan R. et al.
407	21410437	The role of oxidized cytochrome c in regulating mitochondrial reactive oxygen species production and its perturbation in ischaemia. Biochem. J. 493-505.
408	21414309	Fluoxetine affects antioxidant system and promotes apoptotic signaling in Wistar rat liver. [Epub ahead of print] Djordjevic J et al. Eur J Pharmacol.
409	21423629	ShRNA-Targeted Centromere Protein A Inhibits Hepatocellular Carcinoma Growth. Li, Y. et al. 2011. PLoS ONE. 6: -.
410	21432213	Gene expression in primary cultured astrocytes affected by aluminum: alteration of chaperons involved in protein folding. Aremu, DA. et al. 2011. Environ Health Prev Med. 16: 16-24.
411	21478255	Thirdhand smoke: a new dimension to the effects of cigarette smoke on the developing lung. Rehan, VK. et al. 2011. Am. J. Physiol. Lung Cell Mol. Physiol. 301: L1-L8.
412	21479951	Characterization of spontaneous cell death in monolayer cultures of primary hepatocytes. Vinken, M. et al. 2011. Arch. Toxicol. 85: 1589-96.
413	21481476	Tumor necrosis factor alpha pathways develops liver apoptosis in type 1 diabetes mellitus. Ingaramo PI et al. 2011. Mol Immunol. 48: 1397-1407.
414	21494564	Separase phosphosite mutation leads to genome instability and primordial germ cell depletion during oogenesis. Xu, J. et al. 2011. PLoS ONE. 6: -.
415	21503881	Nestin expression is lost in ventricular fibroblasts during postnatal development of the rat heart and re-expressed in scar myofibroblasts. Béguin, PC. et al. 2012. J. Cell. Physiol. 227: 813-20.
416	21505475	Cilostazol minimizes venous ischemic injury in diabetic and normal rats. Wajima, D. et al. 2011. J. Cereb. Blood Flow Metab. 31: 2030-40.
417	21523767	Expression and Function Role of DNA Methyltransferase 1 in Human Bladder Cancer Miao-Fen,
418	21543203	Flavokawain B inhibits growth of human squamous carcinoma cells: Involvement of apoptosis and cell cycle dysregulation in vitro and in vivo. Lin, E. et al. 2012. The Journal of nutritional biochemistry. 23: 368-78.
419	21567405	Human nuclear clusterin mediates apoptosis by interacting with Bcl-XL through C-terminal coiled coil domain. Kim, N. et al. 2012. J. Cell. Physiol. 227: 1157-67.
420	21617209	Anticancer effect of dihydroartemisinin (DHA) in a pancreatic tumor model evaluated by conventional methods and optical imaging. Aung, W. et al. 2011. Anticancer research. 31: 1549-58.
421	21654191	Bid as a potential target of apoptotic effects exerted by low doses of PPARγ and RXR ligands in breast cancer cells. Bonofiglio D, Cione E, Vizza D, Perri M, Pingitore A, Qi H, Catalano S, Rovito D, Genchi G, Andò S. et al.
422	21663398	Geldanamycin analog 17-DMAG limits apoptosis in human peripheral blood cells by inhibition of p53 activation and its interaction with heat-shock protein 90 kDa after exposure to ionizing radiation. Fukumoto, R. et al. 2011. Radiat. Res. 176: 333-45.
423	21671008	Insulin receptor signaling activated by penta-O-galloyl-α-D: -glucopyranose induces p53 and apoptosis in cancer cells. Cao, Y. et al. 2011. Apoptosis. 16: 902-13.
424	21716958	The Differential Effect of Toxoplasma Gondii Infection on the Stability of BCL2-Family Members Involves Multiple Activities. Carmen JC, Sinai AP. et al.
425	21725820	Inhibition of tumor growth by recombinant adenovirus containing human lactoferrin through inducing tumor cell apoptosis in mice bearing EMT6 breast cancer. Wang, J. et al. 2011. Arch. Pharm. Res. 34: 987-95.
426	21760537	Parkin-mediated protection of dopaminergic neurons in a chronic MPTP-minipump mouse model of Parkinson disease. J. Neuropathol. Exp. Neurol. 70: 686-97.
427	21767629	Mycotoxin zearalenone induces AIF- and ROS-mediated cell death through p53- and MAPK-dependent signaling pathways in RAW264.7 macrophages. Yu, JY. et al. 2011. Toxicol In Vitro. 25: 1654-1663.
428	21776823	Antidiabetic drug metformin induces apoptosis in human MCF breast cancer via targeting ERK signaling. Malki, A. et al. 2011. Oncol. Res. 19: 275-85.
429	21799732	p16(INK4a) positively regulates cyclin D1 and E2F1 through negative control of AUF1
430	21850202	Vitamin E modulates cigarette smoke extract-induced cell apoptosis in mouse embryonic cells. Chen ZL, Tao J, Yang J, Yuan ZL, Liu XH, Jin M, Shen ZQ, Wang L, Li HF, Qiu ZG, Wang JF, Wang XW, Li JW. et al.
431	21900958	Essential requirement of cytochrome c release for caspase activation by procaspase-activating compound defined by cellular models. Seervi, M. et al. 2011. Cell Death Dis. 2: -.
432	21904929	Mechanism of functional recovery after repetitive transcranial magnetic stimulation (rTMS) in the subacute cerebral ischemic rat model: neural plasticity or anti-apoptosis? Yoon, KJ. et al. 2011. Exp Brain Res. 214: 549-56.
433	21976111	ATR controls the p21(WAF1/Cip1) protein up-regulation and apoptosis in response to low UV fluences. Al-Khalaf, HH. et al. 2012. Mol. Carcinog. 51: 930-8.
434	22141411	Extracellular recombinant annexin II confers UVC-radiation resistance and increases the Bcl-xL to Bax protein ratios in human UVC-radiation-sensitive cells. Kita, K. et al. 2011. Radiat. Res. 176: 732-42.
435	22180709	Increased extrinsic apoptotic pathway activity in patients with hepatocellular carcinoma following transarterial embolization. Wang SH, Chen LM, Yang WK, Lee JD. et al.
436	22187166	Glucans from the edible mushroom Pleurotus pulmonarius inhibit colitis-associated colon carcinogenesis in mice. Lavi, I. et al. 2012. J. Gastroenterol. 47: 504-18.
437	22189540	Annexin 1 protects against apoptosis induced by serum deprivation in transformed rat retinal ganglion cells. Sun, J. et al. 2012. Mol. Biol. Rep. 39: 5543-51.
438	22207432	Expressions of bax, bcl-2 and Ki-67 in odontogenic keratocysts (Keratocystic Odontogenic Tumor) in comparison with ameloblastomas and radicular cysts. Soluk Tekkeşın M, Mutlu S, Olgaç V. et al.
439	22209856	Inhibition of rapamycin-induced autophagy causes necrotic cell death associated with Bax/Bad mitochondrial translocation.
440	22252435	Survivin expression increases during aging and enhances the resistance of aged human fibroblasts to genotoxic stress. Al-Khalaf, HH. et al. 2013. Age (Dordr). 35: 549-62.
441	22264334	Administration of ON 01210.Na after exposure to ionizing radiation protects bone marrow cells by attenuating DNA damage response. Suman, S. et al. 2012. Radiat Oncol. 7: -.

442	22270152	Protein kinase A signals apoptotic activation in glucose-deprived hepatocytes: participation of reactive oxygen species. Ferretti AC et al. 2012. Apoptosis. 17: 475-491.
443	22309278	Recombination adenovirus-mediated human lactoferrin cDNA inhibits the growth of human MCF-7 breast cancer cells. Wang, J. et al. 2012. J. Pharm. Pharmacol. 64: 457-63.
444	22324429	The chalcone flavokawain B induces G2/M cell-cycle arrest and apoptosis in human oral carcinoma HSC-3 cells through the intracellular ROS generation and downregulation of the Akt/p38 MAPK signaling pathway. Hseu, Y.C. et al. 2012. Journal of agricultural and food chemistry. 60: 2385-97.
445	22336108	Regulation of p14ARF expression by miR-24: a potential mechanism compromising the p53 response during retinoblastoma development. To, KH. et al. 2012. BMC Cancer. 12: 69.
446	22365399	Correlation of seven biological factors (Hsp90a, p53, MDM2, Bcl-2, Bax, Cytochrome C, and Cleaved caspase3) with clinical outcomes of ALK+ anaplastic large-cell lymphoma. Li, HL. et al. 2011. Biomed. Environ. Sci. 24: 630-41.
447	22366758	N-terminally cleaved Bcl-xL mediates ischemia-induced neuronal death. Nat. Neurosci. 15: 574-80.
448	22386815	Ellagic acid protects human keratinocyte (HaCaT) cells against UVA-induced oxidative stress and apoptosis through the upregulation of the HO-1 and Nrf-2 antioxidant genes. Hseu, Y.C. et al. 2012. Food and chemical toxicology : an international journal published for the British Industrial Biological Research Association. 50: 1245-55.
449	22415852	Celecoxib inhibits growth of human autosomal dominant polycystic kidney cyst-lining epithelial cells through the VEGF/Raf/MAPK/ERK signaling pathway. Xu, T. et al. 2012. Molecular biology reports. 39: 7743-53.
450	22427815	Molecular prognostic prediction for locally advanced nasopharyngeal carcinoma by support vector machine integrated approach. Wan, XB. et al. 2012. PLoS ONE. 7: -.
451	22460798	The ubiquitin E3 ligase parkin regulates the proapoptotic function of Bax. Johnson, BN. et al. 2012. Proc. Natl. Acad. Sci. U.S.A. 109: 6283-8.
452	22466850	Essential oil from Cryptomeria japonica induces apoptosis in human oral epidermoid carcinoma cells via mitochondrial stress and activation of caspases.
453	22488775	Valproic acid synergistically enhances the cytotoxicity of clofarabine in pediatric acute myeloid leukemia cells. Xie, C. et al. 2012. Pediatr Blood Cancer. 59: 1245-51.
454	22492309	Sulfuretin from heartwood of Rhus verniciflua triggers apoptosis through activation of Fas, Caspase-8, and the mitochondrial death pathway in HL-60 human leukemia cells.
455	22537845	Overexpression of cellular prion protein (PrP(C)) prevents cognitive dysfunction and apoptotic neuronal cell death induced by amyloid-β (Aβ1.40) administration in mice. Rial, D. et al. 2012. Neuroscience. 215: 79-89.
456	22617775	A feasibility study on gene therapy of pancreatic carcinoma with Ad-PUMA. Wang, H. et al. 2012. Cancer Biol Ther. 13: -.
457	22647853	LEDGF gene silencing impairs the tumorigenicity of prostate cancer DU145 cells by abating the expression of Hsp27 and activation of the Akt/ERK signaling pathway. Bhargavan, B. et al. 2012. Cell death & disease. 3: e316.
458	22648569	The effect of the Ginkgo biloba extract in the expression of Bax, Bcl-2 and bone mineral content of Wistar rats with glucocorticoid-induced osteoporosis. Lucinda, LM. et al. 2013. Phytother Res. 27: 515-20.
459	22659879	Angiotensin II regulates activation of Bim via Rb/E2F1 during apoptosis: involvement of interaction between AMPKβ1/2 and Cdk4. Kim, YC. et al. 2012. Am. J. Physiol. Lung Cell Mol. Physiol. 303: L228-L238.
460	22678884	Multimodal neuroprotection induced by PACAP38 in oxygen-glucose deprivation and middle cerebral artery occlusion stroke models. Lazarovici, P. et al. 2012. J. Mol. Neurosci. 48: 526-40.
461	22688594	Differential expression of Fas family members and Bcl-2 family members in benign versus malignant epithelial ovarian cancer (EOC) in North Indian population. Chaudhry, P. et al. 2012. Mol. Cell. Biochem. 368: 119-26.
462	22701509	Inhibition of Cell Growth and Induction of Apoptosis by Antrodia camphorata in HER-2/neu-Overexpressing Breast Cancer Cells through the Induction of ROS, Depletion of HER-2/neu, and Disruption of the PI3K/Akt Signaling Pathway. Lee, C.C. et al. 2012. Evidence-based complementary and alternative medicine : eCAM. 2012: 702857.
463	22711707	Epstein-Barr virus infection and expression of B-cell oncogenic markers in HIV-related diffuse large B-cell Lymphoma. Chao, C. et al. 2012. Clin. Cancer Res. 18: 4702-12.
464	22740896	Early evaluation of the apoptotic index ratio is useful in predicting the efficacy of chemoradiotherapy in esophageal squamous cell carcinoma. Sobajima, J. et al. 2012. Oncol Lett. 3: 287-292.
465	22746342	Mitochondrial ferritin attenuates β-amyloid-induced neurotoxicity: reduction in oxidative damage through the Erk/P38 mitogen-activated protein kinase pathways. Wu, W.S. et al. 2013. Antioxidants & redox signaling. 18: 158-69.
466	22750966	Extracellular release of annexin II from pancreatic cancer cells and resistance to anticancer drug-induced apoptosis by supplementation of recombinant annexin II. Sato, T. et al. 2012. Pancreas. 41: 1247-54.
467	22811811	Bax expression is a candidate prognostic and predictive marker of colorectal cancer. Katkoori, VR. et al. 2010. J Gastrointest Oncol. 1: 76-89.
468	22847295	A possible cross-talk between autophagy and apoptosis in generating an immune response in melanoma. Hossain, A. et al. 2012. Apoptosis. 17: 1066-78.
469	22884257	Response of liver to lipopolysaccharide treatment in male and female rats. Depboylu, B. et al. 2013. Exp. Toxicol. Pathol. 65: 645-50.
470	22912669	Withaferin A inhibits the proteasome activity in mesothelioma in vitro and in vivo. Yang, H. et al. 2012. PLoS ONE. 7: -.
471	22922085	Camel urine components display anti-cancer properties in vitro. Al-Yousef, N. et al. 2012. J Ethnopharmacol. 143: 819-25.
472	22939933	Mechanisms of natural brassinosteroid-induced apoptosis of prostate cancer cells. Steigerová, J. et al. 2012. Food Chem. Toxicol. 50: 4068-76.
473	22957312	Thyroid hormone receptor β suppresses SV40-mediated tumorigenesis via novel nongenomic actions. Kim DW, Zhao L, Hanover J, Willingham M, Cheng SY. et al.
474	22958292	Anti-leukemic effect of a synthetic compound, (±) trans-dihydronarciclasine (HYU-01) via cell-cycle arrest and apoptosis in acute myeloid leukemia. Kim, SJ. et al. 2012. APMIS. 120: 836-45.
475	22959317	Effects of fluoxetine on plasticity and apoptosis evoked by chronic stress in rat prefrontal cortex. Djordjevic, A. et al. 2012. Eur. J. Pharmacol. 693: 37-44.
476	23047484	10-acetylirciformonin B, a sponge furanoterpenoid, induces DNA damage and apoptosis in leukemia cells. Su, JH. et al. 2012. Molecules. 17: 11839-11848.
477	23049605	The Antitumor Activity of Antrodia camphorata in Melanoma Cells: Modulation of Wnt/β-Catenin Signaling Pathways. Hseu, Y.C. et al. 2012. Evidence-based complementary and alternative medicine : eCAM. 2012: 197309.
478	23142973	Cellular and computational studies of proteasome inhibition and apoptosis induction in human cancer cells by amino acid Schiff base-copper complexes. Zuo, J. et al. 2013. J. Inorg. Biochem. 118: 83-93.
479	23154865	Enhanced heterodimerization of Bax by Bcl-2 mutants improves irradiated cell survival. Verma, YK. et al. 2013. Apoptosis. 18: 212-25.
480	23179253	Dietary fat modifies mitochondrial and plasma membrane apoptotic signaling in skeletal muscle of calorie-restricted mice. López-Domínguez JA. et al. 2012. Age (Dordr).
481	23194196	Human gut flora-fermented nondigestible fraction from cooked bean (Phaseolus vulgaris L.) modifies protein expression associated with apoptosis, cell cycle arrest, and proliferation in human adenocarcinoma colon cancer cells. Campos-Vega R, García-Gasca T, Guevara-Gonzalez R, Ramos-Gomez M, Oomah BD, Loarca-Piña G. et al. 2012. Journal of agricultural and food chemistry. 60: 12443-50.
482	23260670	Gambogic acid is a tissue-specific proteasome inhibitor in vitro and in vivo. Li, X. et al. 2013. Cell Rep. 3: 211-22.
483	23261678	Olive oil oleuropein has anti-breast cancer properties with higher efficiency on ER-negative cells. Elamin, MH. et al. 2013. Food Chem. Toxicol. 53: 310-6.
484	23285100	HDAC inhibitor L-carnitine and proteasome inhibitor bortezomib synergistically exert anti-tumor activity in vitro and in vivo. Huang, H. et al. 2012. PLoS ONE. 7: -.
485	23337877	A feedback regulation between Kindlin-2 and GLI1 in prostate cancer cells. Gao, J. et al. 2013. FEBS Lett. 587: 631-8.
486	23362234	Role of WT1-ZNF224 interaction in the expression of apoptosis-regulating genes. Montano, G. et al. 2013. Hum. Mol. Genet. 22: 1771-82.
487	23364261	Curcumin abates hypoxia-induced oxidative stress based-ER stress-mediated cell death in mouse hippocampal cells (HT22) by controlling Prdx6 and NF-κB regulation. Chhunchha, B. et al. 2013. Am. J. Physiol., Cell Physiol. 304: C636-C655.
488	23376438	Cellular mechanisms of the cytotoxic effects of the zearalenone metabolites α-zearalenol and β-zearalenol on RAW264.7 macrophages. Lu, J. et al. 2013. Toxicol In Vitro. 27: 1007-17.
489	23380911	Suppression of CLIC4/mtCLIC enhances hydrogen peroxide-induced apoptosis in C6 glioma cells Xu Y, Kang J, Yuan Z, Li H, Su J, Li Y, Kong X, Zhang H, Wang W, Sun L. et al.
490	23514663	Distinct Radiosensitivity of Lung Carcinoma Stem-Like Side Population and Main Population Cells. Xia, P. et al. 2013. Cancer Biother. Radiopharm. -.
491	23602052	S1 kills MCF-7/ADR cells more than MCF-7 cells: A protective mechanism of endoplasmic reticulum stress. Song, T. et al. 2013. Biomed. Pharmacother. -.
492	23619020	The anti-cancer activity of Antrodia camphorata against human ovarian carcinoma (SKOV-3) cells via modulation of HER-2/neu signaling pathway. Yang, H.L. et al. 2013. Journal of ethnopharmacology. 148: 254-65.
493	23685194	Neuroprotective effect of suppression of astrocytic activation by arundic acid on brain injuries in rats with acute subdural hematomas. Wajima, D. et al. 2013. Brain Res. 1519: 127-35.
494	23708648	Khat (Catha edulis) generates reactive oxygen species and promotes hepatic cell apoptosis via MAPK activation. Abid, MD. et al. 2013. International journal of molecular medicine. 32: 389-95.
495	23711831	Mechanism for hepato-protective action of Liangxue Huayu Recipe (LHR): blockade of mitochondrial cytochrome c release and caspase activation. Jiang, ZQ. et al. 2013. J Ethnopharmacol. 148: 851-60.
496	23727332	Copper(II) mixed chelate compounds induce apoptosis through reactive oxygen species in neuroblastoma cell line CHP-212. Gutiérrez, AG. et al. 2013. J. Inorg. Biochem. 126: 17-25.
497	23735541	Apoptosis induced by paclitaxel via Bcl-2, Bax and caspases 3 and 9 activation in NB4 human leukaemia cells is not modulated by ERK inhibition. Morales-Cano, D. et al. 2013. Exp. Toxicol. Pathol. -.
498	23782269	Regulation of endoplasmic reticulum stress in rat cortex by p62/ZIP through the Keap1-Nrf2-ARE signalling pathway after transient focal cerebral ischaemia. Wang, W. et al. 2013. Brain injury. 27: 924-33.
499	23805241	CpG-Oligodeoxynucleotide Treatment Protects against Ionizing Radiation-Induced Intestine Injury. Zhang C, Ni J, Li BL, Gao F, Liu H, Liu W, Huang YJ, Cai JM. et al.
500	23805250	Human but Not Laboratory Borna Disease Virus Inhibits Proliferation and Induces Apoptosis in Human Oligodendrocytes In Vitro Li D, Lei Y, Deng J, Zhou C, Zhang Y, Li W, Huang H, Cheng S, Zhang H, Zhang L, Huang R, Liu X, Ma L, Wang X, Li J, Xie P. et al.
501	23840792	Oncogenic Actions of the Nuclear Receptor Corepressor (NCOR1) in a Mouse Model of Thyroid Cancer Fozzatti, L. et al. 2013. PloS one. 8: e67954.
502	23907458	Wip1 suppresses apoptotic cell death through direct dephosphorylation of BAX in response to γ-radiation. Song JY, Ryu SH, Cho YM, Kim YS, Lee BM, Lee SW, Choi J. et al.
503	23907941	Changes of Bcl-2, Bax and Caspase-3 expression in the dermal microvascular endothelial cells and the epidermal layers of the eschar (tache noire) in patients with Mediterranean spotted fever. Baltadzhiev, IG. et al. 2013. Folia histochemica et cytobiologica / Polish Academy of Sciences, Polish Histochemical and Cytochemical Society. 51: 121-6.
504	23928451	Therapeutic and space radiation exposure of mouse brain causes impaired DNA repair response and premature senescence by chronic oxidant production. Suman S, Rodriguez OC, Winters TA, Fornace AJ Jr, Albanese C, Datta K. et al.
505	23954967	Autophagy reduces neuronal damage and promotes locomotor recovery via inhibition of apoptosis after spinal cord injury in rats. Tang, P. et al. 2014. Mol. Neurobiol. 49: 276-87.
506	23957231	Bioefficacy of hydromethanolic extract of tuber of Chlorophytum borivilianum (Safed Musli) for the management of male infertility in cyproterone acetate-treated albino rats. Ray, S. et al. 2014. Andrologia. 46: 659-71.
507	23989931	Loss of a negative feedback loop involving pea3 and cyclin d2 is required for pea3-induced migration in transformed mammary epithelial cells. Ladam, F. et al. 2013. Mol. Cancer Res. 11: 1412-24.
508	23994433	C-peptide preserves the renal microvascular architecture in the streptozotocin-induced diabetic rat. Flynn, ER. et al. J. Diabetes Complicat. 27: 538-47.
509	24026294	Effects of diagnostic ultrasound on HSP70 expression in chorionic villi in rats during early pregnancy and the role of HSP70 in apoptosis in chorionic villi. Liu, H. et al. 2013. Int. J. Mol. Med. 32: 1085-92.
510	24027430	Stress-induced isoforms of MDM2 and MDM4 correlate with high-grade disease and an altered splicing network in pediatric rhabdomyosarcoma. Jacob AG, O'Brien D, Singh RK, Comiskey DF Jr, Littleton RM, Mohammad F, Gladman JT, Widmann MC, Jeyaraj SC, Bolinger C, Anderson JR, Barkauskas DA, Boris-Lawrie K, Chandler DS. et al.
511	24099173	Vitamin D analog EB1089 induces apoptosis in a subpopulation of SGC-7901 gastric cancer cells through a mitochondrial-dependent apoptotic pathway. Wang, W. et al. 2013. Nutr Cancer. 65: 1067-75.
512	24099796	A novel binuclear palladacycle complex inhibits melanoma growth in vitro and in vivo through apoptosis and autophagy. Aliwaini, S. et al. 2013. Biochemical pharmacology. 86: 1650-63.
513	24113180	A novel role for the anti-senescence factor TBX2 in DNA repair and cisplatin resistance. Wansleben, S. et al. 2013. Cell death & disease. 4: e846.
514	24129924	Apoptosis in differentiating C2C12 muscle cells selectively targets Bcl-2-deficient myotubes. Schöneich, C. et al. 2014. Apoptosis. 19: 42-57.
515	24141717	Caspase-cleaved arrestin-2 and BID cooperatively facilitate cytochrome C release and cell death. Kook, S. et al. 2014. Cell death and differentiation. 21: 172-84.
516	24151909	Nicotinamide treatment reduces the levels of oxidative stress, apoptosis, and PARP-1 activity in Aβ(1-42)-induced rat model of Alzheimer's disease. Turunc Bayrakdar, E. et al. 2014. Free Radic. Res. 48: 146-58.
517	24195080	Phytoestrogen α-Zearalanol attenuates homocysteine-induced apoptosis in human umbilical vein endothelial cells. Liu, T. et al. 2013. Biomed Res Int. 2013: 813450.
518	24224909	The resveratrol attenuates ethanol-induced hepatocyte apoptosis via inhibiting ER-related caspase-12 activation and PDE activity in vitro. Liu, L.Q. et al. 2014. Alcoholism, clinical and experimental research. 38: 683-93.
519	24289330	FoxO3a modulation and promotion of apoptosis by interferon-α2b in rat preneoplastic liver. Parody, J.P. et al. 2014. Liver international : official journal of the International Association for the Study of the Liver. 34: 1566-77.
520	24295264	Activation of extrinsic apoptotic pathway from bladder biopsy in patients with interstitial cystitis/painful bladder syndrome. Lee, JD. et al. 2013. Urology. 82: 1451.e7-11.
521	24330704	Eugenol triggers apoptosis in breast cancer cells through E2F1/survivin down-regulation. Al-Sharif, I. et al. 2013. BMC Cancer. 13: 600.
522	24337105	Capsaicin induces apoptosis in PC12 cells through ER stress. Krizanova, O. et al. 2014. Oncol. Rep. 31: 581-8.
523	24349255	Endoplasmic reticulum stress contributes to Helicobacter pylori VacA-induced apoptosis. Akazawa, Y. et al. 2013. PLoS ONE. 8: e82322.
524	24371808	Cell-cycle control of developmentally regulated transcription factors accounts for heterogeneity in human pluripotent cells. Qiao, Z. et al. 2015. Stem Cell Reports. 1: 532-44.
525	24373880	Calcium channel blocker verapamil accelerates gambogic acid-induced cytotoxicity via enhancing proteasome inhibition and ROS generation. Liu, N. et al. 2014. Toxicol In Vitro. 28: 419-25.
526	24385699	Recombinant adenovirus-p53 (Gendicine) sensitizes a pancreatic carcinoma cell line to radiation. Li, J. et al. 2013. Chin. J. Cancer Res. 25: 715-21.
527	24392119	Gold(III)-dithiocarbamato peptidomimetics in the forefront of the targeted anticancer therapy: preclinical studies against human breast neoplasia. Nardon, C. et al. 2014. PLoS ONE. 9: e84248.
528	24427275	Gambogenic acid kills lung cancer cells through aberrant autophagy. Mei, W. et al. 2014. PLoS ONE. 9: e83604.
529	24433123	Ischemic preconditioning attenuates brain injury induced by ischemia/reperfusion during moderate hypothermia low-flow procedures. Ma, ZF. et al. 2014. Int. J. Neurosci. 124: 824-33.
530	24448368	Triptolide induces apoptosis through the SERCA 3 upregulation in PC12 cells. Krizanova, O. et al. 2014. Gen. Physiol. Biophys. 33: 137-44.
531	24486291	Crizotinib (PF-2341066) induces apoptosis due to downregulation of pSTAT3 and BCL-2 family proteins in NPM-ALK(+) anaplastic large cell lymphoma. Hamedani, FS. et al. 2014. Leuk. Res. 38: 503-8.
532	24617288	The ethyl acetate fraction of Sargassum muticum attenuates ultraviolet B radiation-induced apoptotic cell death via regulation of MAPK- and caspase-dependent signaling pathways in human HaCaT keratinocytes. Piao, MJ. et al. 2014. Pharm Biol. 52: 1110-8.
533	24631961	The anti-tumor activity of Antrodia salmonea in human promyelocytic leukemia (HL-60) cells is mediated via the induction of G1 cell-cycle arrest and apoptosis in vitro or in vivo. Hseu, YC. et al. 2014. J Ethnopharmacol. 153: 499-510.
534	24658544	miR-17/20 sensitization of breast cancer cells to chemotherapy-induced apoptosis requires Akt1. Yu, Z. et al. 2014. Oncotarget. 5: 1083-90.
535	24661733	Elevated expression of CD147 in patients with endometriosis and its role in regulating apoptosis and migration of human endometrial cells. Jin, A. et al. 2014. Fertility and sterility. 101: 1681-7.e1.
536	24664502	Interleukin 17A promotes pneumococcal clearance by recruiting neutrophils and inducing apoptosis through a p38 mitogen-activated protein kinase-dependent mechanism in acute otitis media. Wang, W. et al. 2014. Infection and immunity. 82: 2368-77.
537	24691092	Dietary Fat and Aging Modulate Apoptotic Signaling in Liver of Calorie-Restricted Mice. López-Domínguez, J.A. et al. 2014. The journals of gerontology. Series A, Biological sciences and medical sciences.
538	24716481	Phloroglucinol inhibits ultraviolet B radiation-induced oxidative stress in the mouse skin. Piao, MJ. et al. 2014. Int. J. Radiat. Biol. 90: 928-35.
539	24722287	Orally active microtubule-targeting agent, MPT0B271, for the treatment of human non-small cell lung cancer, alone and in combination with erlotinib. Tsai, AC. et al. 2014. Cell Death Dis. 5: e1162.
540	24730533	Neonatal maternal separation up-regulates protein signalling for cell survival in rat hypothalamus. Irles, C. et al. 2014. Stress. 17: 275-84.
541	24743740	Agonist antibodies activating the Met receptor protect cardiomyoblasts from cobalt chloride-induced apoptosis and autophagy. Gallo, S. et al. 2014. Cell death & disease. 5: e1185.
542	24748476	Hsp70 regulates the doxorubicin-mediated heart failure in Hsp70-transgenic mice. Naka K, K. et al. 2014. Cell Stress Chaperones. 19: 853-64.
543	24750068	The evaluation of human tenon's fibroblasts and endothelial cell responses to antifibrotics alone and in combination with α-tocopherol. Engin, KN. et al. 2015. Curr. Eye Res. 40: 19-29.
544	24751974	DHA2, a synthesized derivative of bisbibenzyl, exerts antitumor activity against ovarian cancer through inhibition of XIAP and Akt/mTOR pathway. Pang, Y. et al. 2014. Food and chemical toxicology : an international journal published for the British Industrial Biological Research Association. 69: 163-74.
545	24787013	Reversion of apoptotic resistance of TP53-mutated Burkitt lymphoma B-cells to spindle poisons by exogenous activation of JNK and p38 MAP kinases. Farhat, M. et al. 2014. Cell death & disease. 5: e1201.
546	24895574	Cytotoxic effect of icaritin and its mechanisms in inducing apoptosis in human burkitt lymphoma cell line. Li, ZJ. et al. 2014. Biomed Res Int. 2014: 391512.
547	24902901	The PI3K regulatory subunits p55α and p50α regulate cell death in vivo. Pensa, S. et al. 2014. Cell Death Differ. 21: 1442-50.
548	24912524	A novel proteasome inhibitor suppresses tumor growth via targeting both 19S proteasome deubiquitinases and 20S proteolytic peptidases. Liu, N. et al. 2014. Sci Rep. 4: 5240.
549	24929064	Aging causes exacerbated ischemic brain injury and failure of sevoflurane post-conditioning: role of B-cell lymphoma-2. Dong, P. et al. 2014. Neuroscience. 275: 2-11.
550	24938873	Diverse effect of WWOX overexpression in HT29 and SW480 colon cancer cell lines Nowakowska, M. et al. 2014. Tumour Biol. 35: 9291-9301.
551	24944122	Optimal method for short-term or long-term islet preservation: comparison of islet culture, cold preservation and cryopreservation. Liu, F. et al. 2014. Journal of artificial organs : the official journal of the Japanese Society for Artificial Organs.
552	24955152	Effects of intralipid and caffeic acid phenethyl ester on neurotoxicity, oxidative stress, and acetylcholinesterase activity in acute chlorpyriphos intoxication. Ozkan, U. et al. 2014. Int J Clin Exp Med. 7: 837-46.
553	24966374	NMDA receptor-dependent LTD is required for consolidation but not acquisition of fear memory. Liu, X. et al. 2014. J. Neurosci. 34: 8741-8.
554	24966955	Initiation of premature senescence by Bcl-2 in hypoxic condition. Motor, S. et al. 2014. Int J Clin Exp Pathol. 7: 2446-53.
555	24987917	DNA damage signaling and apoptosis in preinvasive tubal lesions of ovarian carcinoma. Chene, G. et al. 2015. Int. J. Gynecol. Cancer. 25: 761-9.
556	24990082	Histone deacetylation critically determines T cell subset radiosensitivity. J. Immunol. 193: 1451-8.
557	24995577	Human acute promyelocytic leukemia NB4 cells are sensitive to esculetin through induction of an apoptotic mechanism. Rubio, V. et al. 2014. Chemico-biological interactions. 220: 129-39.
558	25017797	Metal-based 2,3-indolinedione derivatives as proteasome inhibitors and inducers of apoptosis in human cancer cells. Zhang, P. et al. 2014. Int. J. Mol. Med. 34: 870-9.
559	25056435	Time course of cerebral hypoperfusion-induced neurodegenerative changes in the cortex of male and female rats. Stanojlović, M. et al. 2014. Folia biologica. 60: 123-32.
560	25084783	Leiomyoma cells in 3-dimensional cultures demonstrate an attenuated response to fasudil, a rho-kinase inhibitor, when compared to 2-dimensional cultures. Malik, M. et al. 2014. Reproductive sciences (Thousand Oaks, Calif.). 21: 1126-38.
561	25109418	Celecoxib induces apoptosis via a mitochondria-dependent pathway in the H22 mouse hepatoma cell line. Shao, D. et al. 2014. Molecular medicine reports. 10: 2093-8.
562	25114230	Interleukin-1 deficiency prolongs ovarian lifespan in mice. Uri-Belapolsky, S. et al. 2014. Proc. Natl. Acad. Sci. U.S.A. 111: 12492-7.
563	25118018	In vitro anticancer activity of loquat tea by inducing apoptosis in human leukemia cells. Zar, PP. et al. 2014. Biosci. Biotechnol. Biochem. 78: 1731-7.
564	25142864	Reduction of myeloid-derived suppressor cells and lymphoma growth by a natural triterpenoid. Radwan, FF. et al. 2015. J. Cell. Biochem. 116: 102-14.
565	25179584	Induction of macrophage cell-cycle arrest and apoptosis by humic acid. Yang, H.L. et al. 2014. Environmental and molecular mutagenesis. 55: 741-50.
566	25228009	Short hairpin RNA-mediated down-regulation of CENP-A attenuates the aggressive phenotype of lung adenocarcinoma cells. Wu, Q. et al. 2014. Cell Oncol (Dordr). 37: 399-407.
567	25231051	Viriditoxin regulates apoptosis and autophagy via mitotic catastrophe and microtubule formation in human prostate cancer cells. Kundu, S. et al. 2014. International journal of oncology. 45: 2331-40.
568	25269557	Effects of doxorubicin and gemcitabine on the induction of apoptosis in breast cancer cells. Zheng, RR. et al. 2014. Oncol. Rep. 32: 2719-25.
569	25296973	CAMK2N1 inhibits prostate cancer progression through androgen receptor-dependent signaling. Wang, T. et al. 2014. Oncotarget. 5: 10293-306.
570	25301748	Brain iron accumulation exacerbates the pathogenesis of MPTP-induced Parkinson's disease. You, LH. et al. 2015. Neuroscience. 284: 234-46.
571	25366571	Does coenzyme-Q have a protective effect against atorvastatin induced myopathy? A histopathological and immunohistochemical study in albino rats. Khalil, MS. et al. 2015. Histol. Histopathol. 30: 383-90.
572	25370579	Epigallocatechin-3-gallate induces the apoptosis of hepatocellular carcinoma LM6 cells but not non-cancerous liver cells. Zhang, Y. et al. 2015. International journal of molecular medicine. 35: 117-24.
573	25405380	Cardioprotective effects of tilianin in rat myocardial ischemia-reperfusion injury. Guo, X. et al. 2015. Mol Med Rep. 11: 2227-33.
574	25415049	Syringaresinol protects against hypoxia/reoxygenation-induced cardiomyocytes injury and death by destabilization of HIF-1α in a FOXO3-dependent mechanism. Cho, S. et al. 2015. Oncotarget. 6: 43-55.
575	25434584	Anticancer agent icaritin induces apoptosis through caspase-dependent pathways in human hepatocellular carcinoma cells. Mol Med Rep. 11: 3094-100.
576	25444915	The palladacycle, AJ-5, exhibits anti-tumour and anti-cancer stem cell activity in breast cancer cells. Aliwaini, S. et al. 2015. Cancer Lett. 357: 206-18.
577	25467746	Effects of diabetes on retinal pigment epithelial cell proliferation and mitogen-activated protein kinase signaling in dark Agouti rats. Al-Hussaini, H. et al. 2015. Exp. Toxicol. Pathol. 67: 117-24.
578	25491731	Wilms' tumor suppressor gene (WT1) suppresses apoptosis by transcriptionally downregulating BAX expression in immature rat granulosa cells. Park, M. et al. 2014. J Ovarian Res. 7: 118.
579	25544676	Autophagy in Normal and Abnormal Early Human Pregnancies. Avagliano, L. et al. 2015. Reprod Sci. 22: 838-44.
580	25546121	Establishment of a rapid drug screening system based on embryonic stem cells. Hong, EJ. et al. 2015. Environ. Toxicol. Pharmacol. 39: 327-38.
581	25550457	PI-PLCβ1b affects Akt activation, cyclin E expression, and caspase cleavage, promoting cell survival in pro-B-lymphoblastic cells exposed to oxidative stress. Piazzi, M. et al. 2015. FASEB J. 29: 1383-94.
582	25552935	MiR-467a is upregulated in radiation-induced mouse thymic lymphomas and regulates apoptosis by targeting Fas and Bax. Gao, F. et al. 2015. Int. J. Biol. Sci. 11: 109-21.
583	25625683	Ameliorating the effect of astragaloside IV on learning and memory deficit after chronic cerebral hypoperfusion in rats. Kim, S. et al. 2015. Molecules. 20: 1904-21.
584	25627828	Role of CtBP2 in the Apoptosis of Retinal Ganglion Cells. Wang, W. et al. 2015. Cell. Mol. Neurobiol. 35: 633-40.
585	25641341	PAC exhibits potent anti-colon cancer properties through targeting cyclin D1 and suppressing epithelial-to-mesenchymal transition. Al-Qasem, A. et al. 2016. Mol. Carcinog. 55: 233-44.
586	25695841	Inhibition of autophagy by 3-methyladenine potentiates sulforaphane-induced cell death of BE(2)-C human neuroblastoma cells. Horwacik, I. et al. 2015. Molecular medicine reports. 12: 535-42.
587	25714330	Effects of resveratrol on benign prostatic hyperplasia by the regulation of inflammatory and apoptotic proteins. Chung, KS. et al. 2015. J. Nat. Prod. 78: 689-94.
588	25714483	Proteasome Dysfunction Associated to Oxidative Stress and Proteotoxicity in Adipocytes Compromises Insulin Sensitivity in Human Obesity. Díaz-Ruiz, A. et al. 2015. Antioxidants & redox signaling. 23: 597-612.
589	25748203	Involvement of mitochondrial pathway of apoptosis in urothelium in ketamine-associated urinary dysfunction. Wu, P. et al. 2015. Am. J. Med. Sci. 349: 344-51.
590	25777481	Repeated low-dose 17β-estradiol treatment prevents activation of apoptotic signaling both in the synaptosomal and cellular fraction in rat prefrontal cortex following cerebral ischemia. Stanojlović, M. et al. Neurochemistry international. 83-84: 1-8.
591	25778982	Identification of biomarkers regulated by rexinoids (LGD1069, LG100268 and Ro25-7386) in human breast cells using Affymetrix microarray. Seo, HS. et al. 2015. Molecular medicine reports. 12: 800-18.
592	25782989	TPN-associated intestinal epithelial cell atrophy is modulated by TLR4/EGF signaling pathways. Freeman, JJ. et al. 2015. FASEB journal : official publication of the Federation of American Societies for Experimental Biology. 29: 2943-58.
593	25828396	Activation of PPARβ/δ protects cardiac myocytes from oxidative stress-induced apoptosis by suppressing generation of reactive oxygen/nitrogen species and expression of matrix metalloproteinases. Barlaka, E. et al. Pharmacol. Res. 95-96: 102-10.
594	25833759	Akt Activation and Inhibition of Cytochrome C Release: Mechanistic Insights into Leptin-promoted Survival of Type II Alveolar Epithelial Cells. Chen, H. et al. 2015. J. Cell. Biochem. 116: 2313-24.
595	25847504	The effects of alpha-lipoic acid on breast of female albino rats exposed to malathion: Histopathological and immunohistochemical study. Omran, OM. et al. 2015. Pathol. Res. Pract. 211: 462-9.
596	25873352	Dickkopf-1 induces apoptosis in the JEG3 and BeWo trophoblast tumor cell lines through the mitochondrial apoptosis pathway. Cui, H. et al. 2015. Int. J. Oncol. 46: 2555-61.
597	25880086	GSK-3β-dependent downregulation of γ-taxilin and αNAC merge to regulate ER stress responses. Hotokezaka, Y. et al. 2015. Cell death & disease. 6: e1719.
598	25891426	Ulinastatin inhibits cerebral ischemia-induced apoptosis in the hippocampus of gerbils. Cho, YS. et al. 2015. Mol Med Rep. 12: 1796-802.
599	25901902	Bone marrow-derived mesenchymal stem cells attenuate acute liver injury and regulate the expression of fibrinogen-like-protein 1 and signal transducer and activator of transcription 3. Zou, Z. et al. 2015. Mol Med Rep. 12: 2089-97.
600	25901924	Protective function of tocilizumab in human cardiac myocytes ischemia reperfusion injury. Cheng, HF. et al. 2015. Asian Pac J Trop Med. 8: 48-52.
601	25914345	JNK and NFκB dependence of apoptosis induced by vinblastine in human acute promyelocytic leukaemia cells. Calviño, E. et al. 2015. Cell biochemistry and function. 33: 211-9.
602	25916043	Impact of GLP-1 analogue on oxidative damage and hepatic regeneration in experimental 70% hepatectomy model. Battal, M. et al. Hepatogastroenterology. 62: 257-60.
603	25936427	Apigenin induces caspase-dependent apoptosis by inhibiting signal transducer and activator of transcription 3 signaling in HER2-overexpressing SKBR3 breast cancer cells. Seo, HS. et al. 2015. Mol Med Rep. 12: 2977-84.
604	25963797	Molecular mechanisms underlying oxytocin-induced cardiomyocyte protection from simulated ischemia-reperfusion. Gonzalez-Reyes, A. et al. 2015. Mol. Cell. Endocrinol. 412: 170-81.
605	26021820	Dermato-protective properties of ergothioneine through induction of Nrf2/ARE-mediated antioxidant genes in UVA-irradiated Human keratinocytes. Hseu, Y.C. et al. 2015. Free radical biology & medicine. 86: 102-17.
606	26022894	Comparative Expression of Apoptotic Markers in Lung Adenocarcinoma and Squamous Cell Carcinoma. Porębska, I. et al. 2015. Adv. Exp. Med. Biol. 873: 101-7.
607	26053091	NDRG4 is a novel oncogenic protein and p53 associated regulator of apoptosis in malignant meningioma cells. Kotipatruni, RP. et al. 2015. Oncotarget. 6: 17594-604.
608	26066836	NGF Expression in Reelin-Deprived Retinal Cells: A Potential Neuroprotective Effect. Balzamino, BO. et al. 2015. Neuromolecular Med. 17: 314-25.
609	26085572	Bee venom suppresses testosterone-induced benign prostatic hyperplasia by regulating the inflammatory response and apoptosis. Chung, KS. et al. 2015. Exp. Biol. Med. (Maywood). 240: 1656-63.
610	26099663	The mechanism of acacetin-induced apoptosis on oral squamous cell carcinoma. Kim, CD. et al. 2015. Archives of oral biology. 60: 1283-98.
611	26101251	FKBP51 employs both scaffold and isomerase functions to promote NF-κB activation in melanoma. Romano, S. et al. 2015. Nucleic Acids Res. 43: 6983-93.
612	26130998	Balsalazide Potentiates Parthenolide-Mediated Inhibition of Nuclear Factor-κB Signaling in HCT116 Human Colorectal Cancer Cells. Kim, HY. et al. 2015. Intestinal research. 13: 233-41.
613	26131191	RhoBTB2 (DBC2) functions as a multifunctional tumor suppressor in thyroid cancer cells via mitochondrial apoptotic pathway. Wang, CJ. et al. 2015. Int J Clin Exp Med. 8: 5954-8.
614	26161241	Neuroprotective Effect of Ulinastatin on Spinal Cord Ischemia-Reperfusion Injury in Rabbits. Liu, B. et al. 2015. Oxid Med Cell Longev. 2015: 624819.
615	26169075	In vitro evaluation of the anti-apoptotic drug Z-VAD-FMK on human ovarian granulosa cell lines for further use in ovarian tissue transplantation. Fransolet, M. et al. 2015. J. Assist. Reprod. Genet. 32: 1551-9.
616	26206332	Silencing Egr1 Attenuates Radiation-Induced Apoptosis in Normal Tissues while Killing Cancer Cells and Delaying Tumor Growth. Zhao, D.Y. et al. 2015. Molecular cancer therapeutics. 14: 2343-52.
617	26219228	Isoliensinine induces apoptosis in triple-negative human breast cancer cells through ROS generation and p38 MAPK/JNK activation. Zhang, X. et al. 2015. Scientific reports. 5: 12579.
618	26219891	Dual PI3K/mTOR inhibitor, XL765 (SAR245409), shows superior effects to sole PI3K [XL147 (SAR245408)] or mTOR [rapamycin] inhibition in prostate cancer cell models. Gravina, GL. et al. 2015. Tumour biology : the journal of the International Society for Oncodevelopmental Biology and Medicine.
619	26239548	Hochu-ekki-to (Bu-zhong-yi-qi-tang), a herbal medicine, enhances cisplatin-induced apoptosis in HeLa cells. Sato, T. et al. 2015. Mol Med Rep. 12: 6215-20.
620	26254599	Mechanism of Tang Luo Ning effect on attenuating of oxidative stress in sciatic nerve of STZ-induced diabetic rats. Yang, X. et al. 2015. J Ethnopharmacol. 174: 1-10.
621	26285136	High Inorganic Phosphate Intake Promotes Tumorigenesis at Early Stages in a Mouse Model of Lung Cancer. Lee, S. et al. 2015. PLoS ONE. 10: e0135582.
622	26300396	Fibrinogen-derived fibrinostatin inhibits tumor growth through anti-angiogenesis. Zhao, C. et al. 2015. Cancer Sci. 106: 1596-606.
623	26306526	HN Protein of Newcastle Disease Virus Induces Apoptosis Through SAPK/JNK Pathway. Rajmani, RS. et al. 2015. Appl. Biochem. Biotechnol. 177: 940-56.
624	26310353	Butein sensitizes HeLa cells to cisplatin through the AKT and ERK/p38 MAPK pathways by targeting FoxO3a. Zhang, L. et al. 2015. Int. J. Mol. Med. 36: 957-66.
625	26355461	6-Shogaol Inhibits Breast Cancer Cells and Stem Cell-Like Spheroids by Modulation of Notch Signaling Pathway and Induction of Autophagic Cell Death. Ray, A. et al. 2015. PloS one. 10: e0137614.
626	26360675	Impact of L-carnitine and Selenium Treatment on Testicular Apoptosis in Rats Exposed to 2.45 GHz Microwave Energy. Saygin, M. et al. 2015. West Indian Med J. 64: 55-61.
627	26379312	Vitamin D3 May Ameliorate the Ketoconazole Induced Adrenal Injury: Histological and Immunohistochemical Studies on Albino Rats. Khalil, MS. et al. 2015. Acta Histochem Cytochem. 48: 103-13.
628	26416955	Shemamruthaa, a Herbal Formulation Induces Apoptosis in Breast Cancer Cells and Inhibits Tumor Progression in Rats. Purushothaman, A. et al. 2015. J Evid Based Complementary Altern Med.
629	26475712	Mesenchymal stem cells suppress neuronal apoptosis and decrease IL-10 release via the TLR2/NFκB pathway in rats with hypoxic-ischemic brain damage. Gu, Y. et al. 2015. Mol Brain. 8: 65.
630	26476632	Food supplementation with rice bran enzymatic extract prevents vascular apoptosis and atherogenesis in ApoE-/- mice. Perez-Ternero, C. et al. 2015. European journal of nutrition.
631	26496900	CXC195 induces apoptosis and endoplastic reticulum stress in human hepatocellular carcinoma cells by inhibiting the PI3K/Akt/mTOR signaling pathway. Chen, XL. et al. 2015. Mol Med Rep. 12: 8229-36.
632	26499801	Fasting and Systemic Insulin Signaling Regulate Phosphorylation of Brain Proteins That Modulate Cell Morphology and Link to Neurological Disorders. Li, M. et al. 2015. J. Biol. Chem. 290: 30030-41.
633	26504614	Protective Effects of Intralipid and Caffeic Acid Phenethyl Ester on Nephrotoxicity Caused by Dichlorvos in Rats. Celik, MM. et al. 2015. J Anal Methods Chem. 2015: 491406.
634	26518459	Effects of chronic cerebral hypoperfusion and low-dose progesterone treatment on apoptotic processes, expression and subcellular localization of key elements within Akt and Erk signaling pathways in rat hippocampus. Stanojlović, M. et al. 2015. Neuroscience. 311: 308-21.
635	26668648	In vitro analysis of the role of the mitochondrial apoptosis pathway in CSBE therapy against human gastric cancer. Ji, YB. et al. 2015. Exp Ther Med. 10: 2403-2409.
636	26689702	Repeated Estradiol Treatment Attenuates Chronic Cerebral Hypoperfusion-Induced Neurodegeneration in Rat Hippocampus. Stanojlović, M. et al. 2016. Cellular and molecular neurobiology. 36: 989-99.
637	26691280	Apoptotic activity of 5-fluorouracil in breast cancer cells transformed by low doses of ionizing α-particle radiation. Ponce-Cusi, R. et al. 2016. Int. J. Oncol. 48: 774-82.
638	26721368	Ethylene glycol ethers induce apoptosis and disturb glucose metabolism in the rat brain. Pomierny, B. et al. 2016. Pharmacological reports : PR. 68: 162-71.
639	26747179	Upregulation of CD44v6 contributes to acquired chemoresistance via the modulation of autophagy in colon cancer SW480 cells. Lv, L. et al. 2016. Tumour biology : the journal of the International Society for Oncodevelopmental Biology and Medicine.
640	26788175	Factors affecting platinum sensitivity in cervical cancer. Kato, R. et al. 2015. Oncol Lett. 10: 3591-3598.
641	26822174	Phenethyl isothiocyanate enhances TRAIL-induced apoptosis in oral cancer cells and xenografts. Yeh, CC. et al. 2016. Clin Oral Investig. 20: 2343-2352.
642	26846577	The hypoxia-mimetic agent $CoCl_2$ induces chemotherapy resistance in LOVO colorectal cancer cells. Yang, G. et al. 2016. Mol Med Rep. 13: 2583-9.
643	26861394	Toxins (Basel). 38.
644	26865401	Ginsenoside Rg1 exerts a protective effect against Aβ25-35-induced toxicity in primary cultured rat cortical neurons through the NF-κB/NO pathway. Wu, J. et al. 2016. Int. J. Mol. Med.
645	26884946	Antitumor effects of crocin on human breast cancer cells. Lu, P. et al. 2015. Int J Clin Exp Med. 8: 20316-22.
646	26898445	RO-3306 prevents postovulatory aging-mediated spontaneous exit from M-II arrest in rat eggs cultured in vitro. Prasad, S. et al. 2016. Biomed. Pharmacother. 78: 216-225.
647	26934204	Cyanidin 3-O-β-Glucoside Ameliorates Ethanol-Induced Acute Liver Injury by Attenuating Oxidative Stress and Apoptosis: The Role of SIRT1/FOXO1 Signaling. Liu, J. et al. 2016. Alcohol. Clin. Exp. Res. 40: 457-66.
648	26939764	Dietary restriction suppresses apoptotic cell death, promotes Bcl-2 and Bcl-xl mRNA expression and increases the Bcl-2/Bax protein ratio in the rat cortex after cortical injury. Lončarević-Vasiljković, N. et al. 2016. Neurochemistry international.
649	26947592	Effects of chronic amiodarone treatment on rat testis. Özkaya, AK. et al. 2016. Acta Histochem. 118: 271-7.
650	26968952	In vitro and in vivo anti-tumor activity of CoQ0 against melanoma cells: inhibition of metastasis and induction of cell-cycle arrest and apoptosis through modulation of Wnt/β-catenin signaling pathways. Hseu, Y.C. et al. 2016. Oncotarget. 7: 22409-26.
651	26973251	Cyclophilin D counteracts P53-mediated growth arrest and promotes Ras tumorigenesis. Bigi, A. et al. 2016. Oncogene. 35: 5132-43.
652	26979341	Paeoniflorin protects Schwann cells against high glucose induced oxidative injury by activating Nrf2/ARE pathway and inhibiting apoptosis. Yang, X. et al. 2016. J Ethnopharmacol. 185: 361-9.
653	26986492	Rubus coreanus Miquel extract causes apoptosis of doxorubicin-resistant NCI/ADR-RES ovarian cancer cells via JNK phosphorylation. Kim, MK. et al. 2016. Mol Med Rep. 13: 4065-72.
654	27008382	Vitamin E, γ-tocotrienol, Protects Against Buthionine Sulfoximine-Induced Cell Death by Scavenging Free Radicals in SH-SY5Y Neuroblastoma Cells. Tan, JK. et al. 2016. Nutr Cancer. 68: 507-17.
655	27012679	Honokiol inhibits sphere formation and xenograft growth of oral cancer side population cells accompanied with JAK/STAT signaling pathway suppression and apoptosis induction. Huang, JS. et al. 2016. BMC Cancer. 16: 245.
656	27035869	The decreased expression of electron transfer flavoprotein β is associated with tubular cell apoptosis in diabetic nephropathy. Wang, H. et al. 2016. Int. J. Mol. Med. 37: 1290-8.
657	27069552	Kisspeptin 10 inhibits the Warburg effect in breast cancer through the Smad signaling pathway: both in vitro and in vivo. Song, GQ. et al. 2016. Am J Transl Res. 8: 188-95.
658	27108485	TLR2/NFκB signalling regulates endogenous IL-6 release from marrow-derived mesenchymal stromal cells to suppress the apoptosis of PC12 cells injured by oxygen and glucose deprivation. Shi, X. et al. 2016. Mol Med Rep. 13: 5358-64.
659	27123245	piR-651 and its function in 95-D lung cancer cells. Yao, J. et al. 2016. Biomed Rep. 4: 546-550.
660	27123496	Anti-proliferation effects of Cistanches salsa on the progression of benign prostatic hyperplasia. Jeon, HJ. et al. 2016. Can. J. Physiol. Pharmacol. 94: 104-11.
661	27133421	Gamma-tocotrienol acts as a BH3 mimetic to induce apoptosis in neuroblastoma SH-SY5Y cells. Tan, JK. et al. 2016. J. Nutr. Biochem. 31: 28-37.
662	27151590	Toona sinensis Inhibits Murine Leukemia WEHI-3 Cells and Promotes Immune Response In Vivo. Yang, H.L. et al. 2016. Integrative cancer therapies.
663	27178634	Piper nigrum ethanolic extract rich in piperamides causes ROS overproduction, oxidative damage in DNA leading to cell cycle arrest and apoptosis in cancer cells. de Souza Grinevicius, VM. et al. 2016. J Ethnopharmacol. 189: 139-47.
664	27191676	Anti-Proliferation Effects of Garlic (Allium sativum L.) on the Progression of Benign Prostatic Hyperplasia. Chung, KS. et al. 2016. Phytother Res. 30: 1197-203.
665	27346131	In vivo inhibition of tumor progression by 5 hydroxy-1,4-naphthoquinone (juglone) and 2-(4-hydroxyanilino)-1,4-naphthoquinone (Q7) in combination with ascorbate. Ourique, F. et al. 2016. Biochem. Biophys. Res. Commun. 477: 640-6.

666	27365043	Protective Effects of Intralipid and Caffeic Acid Phenethyl Ester (CAPE) on Hepatotoxicity and Pancreatic Injury Caused by Dichlorvos in Rats. Alp, H. et al. 2016. Biochem. Genet. 54: 803-815.
667	27441629	Rice Hull Extract Suppresses Benign Prostate Hyperplasia by Decreasing Inflammation and Regulating Cell Proliferation in Rats. Kim, CY. et al. 2016. J Med Food. 19: 746-54.
668	27470394	Hemolin triggers cell survival on fibroblasts in response to serum deprivation by inhibition of apoptosis. Bosch, RV. et al. 2016. Biomed. Pharmacother. 82: 537-46.
669	27488117	IQGAP1 gene silencing induces apoptosis and decreases the invasive capacity of human hepatocellular carcinoma cells. Zoheir, KM. et al. 2016. Tumour Biol. 37: 13927-13939.
670	27499118	Inhibitory effect of metformin combined with gemcitabine on pancreatic cancer cells in vitro and in vivo. Shi, Y. et al. 2016. Mol Med Rep. 14: 2921-8.
671	27565756	Increased DNA double-strand break was associated with downregulation of repair and upregulation of apoptotic factors in rat hippocampus after alcohol exposure. Suman, S. et al. 2016. Alcohol. 54: 45-50.
672	27573911	Cathepsin D is involved in the oxygen and glucose deprivation/reperfusion-induced apoptosis of astrocytes. Liu, J. et al. 2016. Int. J. Mol. Med. 38: 1257-63.
673	27593743	Protective Effects of Intralipid and Caffeic Acid Phenyl Esther (CAPE) on Neurotoxicity Induced by Ethanol in Rats. Basarslan, SK. et al. 2017. Turk Neurosurg. 27: 66-73.
674	27605402	Decursin in Angelica gigas Nakai (AGN) Enhances Doxorubicin Chemosensitivity in NCI/ADR-RES Ovarian Cancer Cells via Inhibition of P-glycoprotein Expression. Choi, HS. et al. 2016. Phytother Res. 30: 2020-2026.
675	27621182	Regulation of the Ku70 and apoptosis-related proteins in experimental diabetic nephropathy. Tunçdemir, M. et al. 2016. Metab. Clin. Exp. 65: 1466-77.
676	27622612	Coadministration of the FNIII14 Peptide Synergistically Augments the Anti-Cancer Activity of Chemotherapeutic Drugs by Activating Pro-Apoptotic Bim. Iyoda, T. et al. 2016. PLoS ONE. 11: e0162525.
677	27633628	Effect of Schiff base Cu(II) complexes on signaling pathways in HT-29 cells. Koňariková, K. et al. 2016. Mol Med Rep. 14: 4436-4444.
678	27705787	Non-canonical PI3K-Cdc42-Pak-Mek-Erk Signaling Promotes Immune-Complex-Induced Apoptosis in Human Neutrophils. Chu, JY. et al. 2016. Cell Rep. 17: 374-386.
679	27710854	Albendazole as a promising molecule for tumor control. Castro, LS. et al. 2016. Redox Biol. 10: 90-99.
680	27748836	Musulju improves benign prostatic hyperplasia by regulating inflammatory and apoptotic proteins. Ju, JY. et al. 2016. Mol Med Rep. 14: 4692-4698.
681	27748859	Human parvovirus B19 antibodies induce altered membrane protein expression and apoptosis of BeWo trophoblasts. Tzang, BS. et al. 2016. Mol Med Rep. 14: 4399-4406.
682	27779646	Heparin-binding epidermal growth factor-like growth factor and hepatocyte growth factor inhibit cholestatic liver injury in mice through different mechanisms. Sakamoto, K. et al. 2016. Int. J. Mol. Med. 38: 1673-1682.
683	27779649	Effect of curcumin and paclitaxel on breast carcinogenesis. Quispe-Soto, ET. et al. 2016. Int. J. Oncol. 49: 2569-2577.
684	27807255	Epigallocatechin-3-gallate protects against hepatic ischaemia-reperfusion injury by reducing oxidative stress and apoptotic cell death. Tak, E. et al. 2016. J. Int. Med. Res. 44: 1248-1262.
685	27817140	Presence of encircling granulosa cells protects against oxidative stress-induced apoptosis in rat eggs cultured in vitro. Tiwari, M. et al. 2016. Apoptosis.
686	27888018	1400W ameliorates acute hypobaric hypoxia/reoxygenation-induced cognitive deficits by suppressing the induction of inducible nitric oxide synthase in rat cerebral cortex microglia. Shi, Q. et al. 2017. Behav. Brain Res. 319: 188-199.
687	27911270	SAHA and/or MG132 reverse the aggressive phenotypes of glioma cells: An in vitro and vivo study. Yang, XF. et al. 2017. Oncotarget. 8: 3156-3169.
688	27919040	Canstatin induces apoptosis in gastric cancer xenograft growth in mice through the mitochondrial apoptotic pathway. Xing, YN. et al. 2014. Biosci. Rep. 34:
689	27951409	The contribution of keratinocytes in capecitabine-stimulated hand-foot-syndrome. Chen, M. et al. 2017. Environ. Toxicol. Pharmacol. 49: 81-88.
690	27995757	Protective effects of β-sheet breaker α/β-hybrid peptide against amyloid β-induced neuronal apoptosis in vitro. Kumar, S. et al. 2016. Chem Biol Drug Des.
691	28005083	Attenuating effects of caffeic acid phenethyl ester with intralipid on hepatotoxicity of chlorpyrifos in the case of rats. Dokuyucu, R. et al. 2016. Med Pr. 67: 743-749.
692	28053547	Anticancer activities of epigallocatechin-3-gallate against cholangiocarcinoma cells. Kwak, TW. et al. 2017. Onco Targets Ther. 10: 137-144.
693	28097640	Priming increases the anti-tumor effect and therapeutic window of 177Lu-octreotate in nude mice bearing human small intestine neuroendocrine tumor GOT1. Dalmo, J. et al. 2017. EJNMMI Res. 7: 6.
694	28102575	Bcl-2 family in non-small cell lung cancer: its prognostic and therapeutic implications. Sun, PL. et al. 2017. Pathol. Int. 67: 121-130.
695	28123477	Dexmedetomidine alleviates cerebral ischemia-induced short-term memory impairment by inhibiting the expression of apoptosis-related molecules in the hippocampus of gerbils. Choi, IY. et al. 2017. Exp Ther Med. 13: 107-116.
696	28123591	Isoimperatorin induces apoptosis of the SGC-7901 human gastric cancer cell line via the mitochondria-mediated pathway. Tong, K. et al. 2017. Oncol Lett. 13: 518-524.
697	28160569	Mitotic cell death induction by targeting the mitotic spindle with tubulin-inhibitory indole derivative molecules. Di Cesare, E. et al. 2017. Oncotarget. 8: 19738-19759
698	28178652	STAT3 is required for MiR-17-5p-mediated sensitization to chemotherapy-induced apoptosis in breast cancer cells. Liao, XH. et al. 2017. Oncotarget. 8: 15763-15774.
699	28219700	Inhibition of ROS production, autophagy or apoptosis signaling reversed the anticancer properties of Antrodia salmonea in triplenegative breast cancer (MDA-MB-231) cells Chia-Ting , et al. 2017. Food and Chemical Toxicology. 103: 1-17.
700	28223017	Novel BCL2 inhibitor, Disarib induces apoptosis by disruption of BCL2-BAK interaction. Vartak, SV. et al. 2017. Biochem. Pharmacol. 131: 16-28.
701	28245011	Inhibitory effects of different substituted transition metal-based krebs-type sandwich structures on human hepatocellular carcinoma cells. Wang, L. et al. 2017. Dalton Trans. 46: 2874-2883.
702	28260081	Minocycline attenuates sevoflurane-induced cell injury via activation of Nrf2. Tian, Y. et al. 2017. Int. J. Mol. Med. 39: 869-878.
703	28321485	Initial autophagic protection switches to disruption of autophagic flux by lysosomal instability during cadmium stress accrual in renal NRK-52E cells. Lee, WK. et al. 2017. Arch. Toxicol. 91: 3225-3245.
704	28323051	Anticancer and anti-angiogenic activities of extract from Actinidia eriantha Benth root. Wu, JG. et al. 2017. J Ethnopharmacol. 203: 1-10.
705	28348080	A positive feedback loop between Pim-1 kinase and HBP1 transcription factor contributes to hydrogen peroxide-induced premature senescence and apoptosis. Wang, S. et al. 2017. J. Biol. Chem. 292: 8207-8222.
706	28369131	Liver X receptors activation, through TO901317 binding, reduces neuroinflammation in Parkinson's disease. Paterniti, I. et al. 2017. PLoS ONE. 12: e0174470.
707	28373418	Differing Mechanisms of Death Induction by Fluorinated Taxane SB-T-12854 in Breast Cancer Cells. Jelinek, M. et al. 2017. Anticancer Res. 37: 1581-1590.
708	28409209	2-Acetyl-5-tetrahydroxybutyl imidazole (THI) protects 661W cells against oxidative stress. Fabiani, C. et al. 2017. Naunyn Schmiedebergs Arch. Pharmacol. 390: 741-751.
709	28439094	Selective Targeting of Cancer Cells by Oxidative Vulnerabilities with Novel Curcumin Analogs. Pignanelli, C. et al. 2017. Sci Rep. 7: 1105.
710	28450936	The traditional Korean herbal medicine Ga-Gam-Nai-Go-Hyan suppresses testosterone-induced benign prostatic hyperplasia by regulating inflammatory responses and apoptosis. Shin, SJ. et al. 2017. Exp Ther Med. 13: 1025-1031.
711	28454332	Zoledronic acid reverses cisplatin resistance in nasopharyngeal carcinoma cells by activating the mitochondrial apoptotic pathway. You, Y. et al. 2017. Oncol Lett. 13: 1840-1846.
712	28475602	Prolactin-induced neuroprotection against glutamate excitotoxicity is mediated by the reduction of [Ca2+]i overload and NF-κB activation. Rivero-Segura, NA. et al. 2017. PLoS ONE. 12: e0176910.
713	28513573	Nrf2 Inhibits Periodontal Ligament Stem Cell Apoptosis under Excessive Oxidative Stress. Liu, Y. et al. 2017. Int J Mol Sci. 18:
714	28531892	Emery-Dreifuss Muscular Dystrophy-Associated Mutant Forms of Lamin A Recruit the Stress Responsive Protein Ankrd2 into the Nucleus, Affecting the Cellular Response to Oxidative Stress. Angori, S. et al. 2017. Cell. Physiol. Biochem. 42: 169-184.
715	28551013	Citrus nomilin down-regulates TNF-α-induced proliferation of aortic smooth muscle cells via apoptosis and inhibition of IκB. Kim, J. et al. 2017. Eur. J. Pharmacol.
716	28586001	Apoptosis is induced by docosahexaenoic acid in breast cancer cells via death receptor and mitochondria-mediated pathways. Xue, M. et al. 2017. Mol Med Rep. 16: 978-982.
717	28589243	RNAi targeting STMN alleviates the resistance to taxol and collectively contributes to down regulate the malignancy of NSCLC cells in vitro and in vivo. Long, D. et al. 2017. Cell Biol. Toxicol. 34: 7-21.
718	28627468	A novel chalcone derivative, LQFM064, induces breast cancer cells death via p53, p21, KIT and PDGFRA. Cabral, BLS. et al. 2017. Eur J Pharm Sci. 107: 1-15.
719	28627637	Lentiviral vector-mediated co-overexpression of VEGF and Bcl-2 improves mesenchymal stem cell survival and enhances paracrine effects in vitro. Ni, X. et al. 2017. Int. J. Mol. Med. 40: 418-426.
720	28646810	Synergistic Anti-Breast-Cancer Effects of Combined Treatment With Oleuropein and Doxorubicin In Vivo. Elamin, MH. et al. 2017. Altern Ther Health Med.
721	28656004	Berberine Reverses Hypoxia-induced Chemoresistance in Breast Cancer through the Inhibition of AMPK- HIF-1α. Pan, Y. et al. 2017. Int. J. Biol. Sci. 13: 794-803.
722	28672952	Emodin protects against oxidative stress and apoptosis in HK-2 renal tubular epithelial cells after hypoxia/reoxygenation. Chen, H. et al. 2017. Exp Ther Med. 14: 447-452.
723	28692662	Potential malignant transformation in the gastric mucosa of immunodeficient mice with persistent Mycoplasma penetrans infection. Cao, S. et al. 2017. PLoS ONE. 12: e0180514.
724	28708259	Rapid modulation of the silent information regulator 1 by melatonin after hypoxia-ischemia in the neonatal rat brain. Carloni, S. et al. 2017. J. Pineal Res. 63:
725	28740353	Anti-apoptotic effect of banhasasim-tang on chronic acid reflux esophagitis. Shin, MR. et al. 2017. World J. Gastroenterol. 23: 4644-4653.
726	28762326	Long Noncoding RNA Urothelial Carcinoma Associated 1 Promotes the Proliferation and Metastasis of Human Lung Tumor Cells by Regulating MicroRNA-144. Li, D. et al. 2017. Oncol. Res.
727	28772050	The neuroprotective agent Rasagiline mesylate attenuates cardiac remodeling after experimental myocardial infarction. Varela, A. et al. 2017. ESC Heart Fail. 4: 331-340.
728	28775788	Berberine Enhances Chemosensitivity and Induces Apoptosis Through Dose-orchestrated AMPK Signaling in Breast Cancer. Pan, Y. et al. 2017. J Cancer. 8: 1679-1689.
729	28777347	γ-Tocotrienol Inhibits Proliferation and Induces Apoptosis Via the Mitochondrial Pathway in Human Cervical Cancer HeLa Cells. Xu, W. et al. 2017. Molecules. 22:
730	28789443	Anticancer effects of an extract from the scallop Patinopecten yessoensis on MCF-7 human breast carcinoma cells. Lee, C. et al. 2017. Oncol Lett. 14: 2207-2217.
731	28808311	Antitumor properties of Coenzyme Q0 against human ovarian carcinoma cells via induction of ROS-mediated apoptosis and cytoprotective autophagy You-Cheng , et al. Scientific Reports. 7: 8062.
732	28841232	Inhibiting heat shock protein 90 and the ubiquitin-proteasome pathway impairs metabolic homeostasis and leads to cell death in human pancreatic cancer cells. Belalcazar, A. et al. 2017. Cancer. 123: 4924-4933.
733	28849082	Short-term vagus nerve stimulation reduces myocardial apoptosis by downregulating microRNA-205 in rats with chronic heart failure. Xuan, Y. et al. 2017. Mol Med Rep. 16: 5847-5854.
734	28849092	Mdivi-1 attenuates sodium azide-induced apoptosis in H9c2 cardiac muscle cells. Xu, X. et al. 2017. Mol Med Rep.
735	28861322	14-3-3ζ loss impedes oncogene-induced mammary tumorigenesis and metastasis by attenuating oncogenic signaling. Joshi, S. et al. 2017. Am J Cancer Res. 7: 1654-1664.
736	28899498	Galangin ameliorates cisplatin induced nephrotoxicity in vivo by modulation of oxidative stress, apoptosis and inflammation through interplay of MAPK signaling cascade. Tomar, A. et al. 2017. Phytomedicine. 34: 154-161.
737	28915612	The roles of ING5 in gliomas: a good marker for tumorigenesis and a potential target for gene therapy. Zhao, S. et al. 2017. Oncotarget. 8: 56558-56568.
738	28918503	CoQ0-induced mitochondrial PTP opening triggers apoptosis via ROS-mediated VDAC1 upregulation in HL-60 leukemia cells and suppresses tumor growth in athymic nude mice/xenografted nude mice You-Cheng, et al. 2018. Arch Toxicol. 92: 301322.
739	28935920	Inhibition of PDE2 reverses beta amyloid induced memory impairment through regulation of PKA/PKG-dependent neuro-inflammatory and apoptotic pathways. Wang, L. et al. 2017. Sci Rep. 7: 12044.
740	28943902	Khat promotes human breast cancer MDA-MB-231 cell apoptosis via mitochondria and MAPK-associated pathways. Lu, Y. et al. 2017. Oncol Lett. 14: 3947-3952.
741	28944910	SH003-induced G1 phase cell cycle arrest induces apoptosis in HeLa cervical cancer cells. Lee, KM. et al. 2017. Mol Med Rep. 16: 8237-8244.
742	28968984	A novel deubiquitinase inhibitor b-AP15 triggers apoptosis in both androgen receptor-dependent and -independent prostate cancers. Cai, J. et al. 2017. Oncotarget. 8: 63232-63246
743	29098117	Human Chorionic Gonadotropin Mediated Generation of Reactive Oxygen Species Is Sufficient to Induce Meiotic Exit but Not Apoptosis in Rat Oocytes. Tiwari, M. et al. 2017. Biores Open Access. 6: 110-122.
744	29110485	Discovery of Aromatic Carbamates that Confer Neuroprotective Activity by Enhancing Autophagy and Inducing the Anti-Apoptotic Protein B-Cell Lymphoma 2 (Bcl-2). Kinarivala, N. et al. 2017. J. Med. Chem. 60: 9739-9756.
745	29111385	JNK1 inhibition by Licochalcone A leads to neuronal protection against excitotoxic insults derived of kainic acid. Busquets, O. et al. 2018. Neuropharmacology. 131: 440-452.
746	29138676	Resveratrol Modulation of Protein Expression inparkin-Mutant Human Skin Fibroblasts: A Proteomic Approach. Vergara, D. et al. 2017. Oxid Med Cell Longev. 2017: 2198243.
747	29138678	Febuxostat Modulates MAPK/NF-κBp65/TNF-αSignaling in Cardiac Ischemia-Reperfusion Injury. Khan, SI. et al. 2017. Oxid Med Cell Longev. 2017: 8095825.
748	29183790	17β-estradiol and tamoxifen protect mice from manganese-induced dopaminergic neurotoxicity. Pajarillo, E. et al. 2017. Neurotoxicology.
749	29212519	GPER mediates the angiocrine actions induced by IGF1 through the HIF-1α/VEGF pathway in the breast tumor microenvironment. Wei, Z. et al. 2003. Breast Cancer Res. 19: 129.
750	29217266	Auriculasin-induced ROS causes prostate cancer cell death via induction of apoptosis. Cho, HD. et al. 2017. Food Chem. Toxicol. 111: 660-669.
751	29257260	Upregulation of miR-185 promotes apoptosis of the human gastric cancer cell line MGC803. Fan, L. et al. 2018. Mol Med Rep. 17: 3115-3122.
752	29258835	The GluN1/GluN2B NMDA receptor and metabotropic glutamate receptor 1 negative allosteric modulator has enhanced neuroprotection in a rat subarachnoid hemorrhage model. Zhang, Z. et al. 2018. Exp. Neurol. 301: 13-25.
753	29272311	Genomic alterations during p53-dependent apoptosis induced by γ-irradiation of Molt-4 leukemia cells. Hage-Sleiman, R. et al. 2017. PLoS ONE. 12: e0190221.
754	29279520	Taurine Protects Primary Neonatal Cardiomyocytes Against Apoptosis Induced by Hydrogen Peroxide. Wang, J. et al. 2017. Int Heart J.
755	29328385	Tormentic acid inhibits IL-1β-induced chondrocyte apoptosis by activating the PI3K/Akt signaling pathway. Yang, Y. et al. 2018. Mol Med Rep. 17: 4753-4758.
756	29328418	microRNA-3941 targets IGF2 to control LPS-induced acute pneumonia in A549 cells. Fei, S. et al. 2018. Mol Med Rep. 17: 4019-4026.
757	29344241	Combined pitavastatin and dacarbazine treatment activates apoptosis and autophagy resulting in synergistic cytotoxicity in melanoma cells. Al-Qatati, A. et al. 2017. Oncol Lett. 14: 7993-7999.
758	29360968	Porcine parvovirus infection impairs progesterone production in luteal cells through mitogen-activated protein kinases, p53, and mitochondria-mediated apoptosis. Zhang, L. et al. 2018. Biol. Reprod. 98: 558-569.
759	29389945	Intermittent hypoxia causes histological kidney damage and increases growth factor expression in a mouse model of obstructive sleep apnea. Abuyassin, B. et al. 2018. PLoS ONE. 13: e0192084.
760	29396519	An Accord of Nuclear Receptor Expression in M. tuberculosis Infected Macrophages and Dendritic Cells. Moréno, SG. et al. 2018. Sci Rep. 8: 2296.
761	29416111	IRE1α siRNA relieves endoplasmic reticulum stress-induced apoptosis and alleviates diabetic peripheral neuropathy in vivo and in vitro. Yao, W. et al. 2018. Sci Rep. 8: 2579.
762	29436595	Anti-apoptotic effects of glycosaminoglycans via inhibition of ERK/AP-1 signaling in TNF-α-stimulated human dermal fibroblasts. Na, J. et al. 2018. Int. J. Mol. Med. 41: 3090-3098.
763	29436666	Daidzein induces choriocarcinoma cell apoptosis in a dose-dependent manner via the mitochondrial apoptotic pathway. Zheng, W. et al. 2018. Mol Med Rep. 17: 6093-6099.
764	29441020	Eriodictyol Attenuates Myocardial Ischemia-Reperfusion Injury through the Activation of JAK2. Li, D. et al. 2018. Front Pharmacol. 9: 33.
765	29467412	IL-32 gamma reduces lung tumor development through upregulation of TIMP-3 overexpression and hypomethylation. Biondi-Zoccai, GG. et al. 2018. Cell Death Dis. 9: 306.
766	29483377	Laparoscopic management of retroperitoneal injuries from penetrating abdominal trauma in haemodynamically stable patients. Koto, MZ. et al. J Minim Access Surg. 15: 25-30.
767	29494356	A novel penicillin derivative induces antitumor effect in melanoma cells. Blank, V. et al. 2018. Anticancer Drugs. 29: 416-428.
768	29553887	Seasonal Changes in Metabolism and Cellular Stress Phenomena in the Gilthead Sea Bream (Sparus aurata). Feidantsis, K. et al. Physiol. Biochem. Zool. 91: 878-895.
769	29563994	Shikonin induces mitochondria-mediated apoptosis and attenuates epithelial-mesenchymal transition in cisplatin-resistant human ovarian cancer cells. Nosaka, Y. et al. 2018. Oncol Lett. 15: 5417-5424.
770	29606027	Effects of cisplatin-5-fluorouracil combination therapy on oxidative stress, DNA damage, mitochondrial apoptosis, and death receptor signalling in retinal pigment epithelium cells. Güçlü, H. et al. 2018. Cutan Ocul Toxicol. 1-14.
771	29623239	Increased resistance to apoptosis during differentiation and syncytialization of BeWo choriocarcinoma cells. Wei, BR. et al. 2012. Adv Biosci Biotechnol. 3: 805-813.
772	29626597	Placentas from women with pregnancy-associated venous insufficiency show villi damage with evidence of hypoxic cellular stress. García-Honduvilla, N. et al. 2018. Hum. Pathol.
773	29669932	Nardilysin controls intestinal tumorigenesis through HDAC1/p53-dependent transcriptional regulation. Kanda, K. et al. 2018. JCI Insight. 3:
774	29682182	Anti-cancerous effect of cis-khellactone from Angelica amurensis through the induction of three programmed cell deaths. Jung, S. et al. 2018. Oncotarget. 9: 16744-16757.
775	29717765	Bromodomain-containing protein 4 is critical for the antiproliferative and pro-apoptotic effects of gambogic acid in anaplastic thyroid cancer. Wang, Y. et al. 2018. Int. J. Mol. Med. 42: 161-170.
776	29770446	Anticancer activity of tetrandrine by inducing pro-death apoptosis and autophagy in human gastric cancer cells. Bai, XY. et al. 2018. J. Pharm. Pharmacol.
777	29770794	Ursodeoxycholic acid protects interstitial Cajal-like cells in the gallbladder from undergoing apoptosis by inhibiting TNF-α expression. Wan, JF. et al. 2018. Acta Pharmacol Sin. 39: 1493-1500.
778	29781255	Combination therapy of canine osteosarcoma with canine bone marrow stem cells, bone morphogenetic protein and carboplatin in an in vivo model. Rici, REG. et al. 2018. Vet Comp Oncol.
779	29789524	Hirsutine induces mPTP-dependent apoptosis through ROCK1/PTEN/PI3K/GSK3β pathway in human lung cancer cells. Zhang, R. et al. 2018. Cell Death Dis. 9: 598.
780	29790427	Simulated microgravity culture enhances the neuroprotective effects of human cranial bone-derived mesenchymal stem cells in traumatic brain injury. Otsuka, T. et al. 2018. Stem Cells Dev.
781	29843746	miR-519a enhances chemosensitivity and promotes autophagy in glioblastoma by targeting STAT3/Bcl2 signaling pathway. Li, H. et al. 2018. J Hematol Oncol. 11: 70.
782	29859204	Neuroprotective effect of zolpidem against glutamate-induced toxicity is mediated via the PI3K/Akt pathway and inhibited by PK11195. Jembrek, MJ. et al. 2018. Toxicology.
783	29860221	Simvastatin ameliorates diabetic nephropathy by attenuating oxidative stress and apoptosis in a rat model of streptozotocin-induced type 1 diabetes. Al-Rasheed, NM. et al. 2018. Biomed. Pharmacother. 105: 290-298.
784	29869056	L-carnosine induces apoptosis/cell cycle arrest via suppression of NF-κB/STAT1 pathway in HCT116 colorectal cancer cells. Lee, J. et al. 2018. In Vitro Cell. Dev. Biol. Anim.
785	29872701	Targeting ornithine decarboxylase (ODC) inhibits esophageal squamous cell carcinoma progression. He, W. et al. 2017. NPJ Precis Oncol. 1: 13.
786	29886231	In vivo antitumor activity of by-products of Passiflora edulis f. flavicarpa Deg. Rich in medium and long chain fatty acids evaluated through oxidative stress markers, cell cycle arrest and apoptosis induction. Mota, NSRS. et al. 2018. Food Chem. Toxicol. 118: 557-565.
787	29901126	Synergistic apoptotic effects of silibinin in enhancing paclitaxel toxicity in human gastric cancer cell lines. 2018. Mol Med Rep. 18: 1835-1841.
788	29916532	Resveratrol induced reactive oxygen species and endoplasmic reticulum stress-mediated apoptosis, and cell cycle arrest in the A375SM malignant melanoma cell line. Heo, JR. et al. 2018. Int. J. Mol. Med.
789	29928358	Resveratrol suppresses human cervical carcinoma cell proliferation and elevates apoptosis via the mitochondrial and p53 signaling pathways. Li, L. et al. 2018. Oncol Lett. 15: 9845-9851.
790	29937996	Synuclein gamma expression enhances radiation resistance of breast cancer cells. Tian, L. et al. 2018. Oncotarget. 9: 27435-27447
791	29959408	Mechanistic insights into the augmented effect of bone marrow mesenchymal stem cells and thiazolidinediones in streptozotocin-nicotinamide induced diabetic rats. Hamza, AA. et al. 2018. Sci Rep. 8: 9827.
792	29972271	Targeting mitochondria with folic acid and vitamin B12 ameliorates nicotine mediated islet cell dysfunction. Bhattacharjee, A. et al. 2018. Environ. Toxicol.
793	30015841	The tumorigenic role of DSPP and its potential regulation of the unfolded protein response and ER stress in oral cancer cells. Gkouveris, I. et al. 2018. Int. J. Oncol. 53: 1743-1751.
794	30017436	Treatment with crocin improves cardiac dysfunction by normalizing autophagy and inhibiting apoptosis in STZ-induced diabetic cardiomyopathy. Feidantsis, K. et al. 2018. Nutr Metab Cardiovasc Dis. 28: 952-961.
795	30021393	Therapeutic efficacy of zingerone against vancomycin-induced oxidative stress, inflammation, apoptosis and aquaporin 1 permeability in rat kidney. Kandemir, FM. et al. 2018. Biomed. Pharmacother. 105: 981-991.
796	30057418	A dual role of miR-22 modulated by RelA/p65 in resensitizing fulvestrant-resistant breast cancer cells to fulvestrant by targeting FOXP1 and HDAC4 and constitutive acetylation of p53 at Lys382. Wang, B. et al. 2018. Oncogenesis. 7: 54.
797	30062060	Autophagic cell death participates in POMC-induced melanoma suppression. Wu, JC. et al. 2018. Cell Death Discov. 5: 11.
798	30071508	Knockdown of Mir-135b Sensitizes Colorectal Cancer Cells to Oxaliplatin-Induced Apoptosis Through Increase of FOXO1. Qin, Y. et al. 2018. Cell. Physiol. Biochem. 48: 1628-1637.
799	30087701	Roles of B7-H3 in Cervical Cancer and Its Prognostic Value. Han, S. et al. 2018. J Cancer. 9: 2612-2624.
800	30103475	4-Acetyl-Antroquinonol B Suppresses SOD2-Enhanced Cancer Stem Cell-Like Phenotypes and Chemoresistance of Colorectal Cancer Cells by Inducing hsa-miR-324 re-Expression. Bamodu, OA. et al. 2018. Cancers (Basel). 10:
801	30116354	TTB2 induces apoptosis in Ewing sarcoma cells. Zou, X. et al. 2018. Exp Ther Med. 16: 1021-1025.
802	30117083	Paeoniflorin attenuates atRAL-induced oxidative stress, mitochondrial dysfunction and endoplasmic reticulum stress in retinal pigment epithelial cells via triggering Ca2+/CaMKII-dependent activation of AMPK. Zhu, X. et al. 2018. Arch. Pharm. Res.
803	30132563	Serum-free-medium-type mesenchymal stem cell culture supernatant exerts a protective effect on A549 lung epithelial cells in acute lung injury induced by H2O2. Wu, J. et al. 2018. Oncol. Rep. 40: 3033-3039.
804	30132966	Apoptotic effect of resveratrol on human T-ALL cell line CCRF-CEM is unlikely exerted through alteration of BAX and BCL2 promoter methylation. Zadi Heydarabad, M. et al. 2018. J. Cell. Biochem.
805	30209133	The CDK 9-cyclin T1 complex mediates saturated fatty acid-induced vascular calcification by inducing expression of the transcription factor CHOP. Shiozaki, Y. et al. 2018. J. Biol. Chem.
806	30217003	Cryptotanshinone Induces Cell Cycle Arrest and Apoptosis of NSCLC Cells through the PI3K/Akt/GSK-3β Pathway. Kim, SA. et al. 2018. Int J Mol Sci. 19:
807	30217947	HADC2 regulates the diabetic vascular endothelial dysfunction by targeting MnSOD. Hou, Q. et al. 2018. Biosci. Rep.
808	30226569	ISL1 promotes cancer progression and inhibits cisplatin sensitivity in triple-negative breast cancer cells. Zhang, Y. et al. 2018. Int. J. Mol. Med. 42: 2343-2352.
809	30231080	Role of p53 and transcription-independent p53-induced apoptosis in shear-stimulated megakaryocytic maturation, particle generation, and platelet biogenesis. Luff, SA. et al. 2018. PLoS ONE. 13: e0203991.
810	30248657	Synthesis and antiproliferative activity of podocarpane and totarane derivatives. Mahdjour, S. et al. 2018. Eur J Med Chem. 158: 863-873.
811	30320380	Interferon γ suppresses dentin sialophosphoprotein in oral squamous cell carcinoma cells resulting in antitumor effects, via modulation of the endoplasmic reticulum response. Gkouveris, I. et al. 2018. Int. J. Oncol.
812	30322186	Anti-Proliferative Effects of HBX-5 on Progression of Benign Prostatic Hyperplasia. Jin, BR. et al. 2018. Molecules. 23:
813	30365120	Galla Rhois water extract inhibits lung metastasis by inducing AMPK-mediated apoptosis and suppressing metastatic properties of colorectal cancer cells. Mun, JG. et al. 2018. Oncol. Rep.
814	30366984	Macrophage migration inhibitory factor regulates mitochondrial dynamics and cell growth of human cancer cell lines through CD74-NFκB signaling. De, R. et al. 2018. J. Biol. Chem.
815	30378157	Endoplasmic reticulum stress, autophagic and apoptotic cell death, and immune activation by a natural triterpenoid in human prostate cancer cells. Johnson, BM. et al. 2018. J. Cell. Biochem.
816	30390334	Inhibitor of growth 4 inhibits cell proliferation, migration, and induces apoptosis of renal cell carcinoma cells. Sun, J. et al. 2018. J. Cell. Biochem.
817	30405447	Compound Danshen Dripping Pill Inhibits Retina Cell Apoptosis in Diabetic Rats. Zhang, Q. et al. 2018. Front Physiol. 9: 1501.
818	30405790	Ganoderic acid A exerts antitumor activity against MDA-MB-231 human breast cancer cells by inhibiting the Janus kinase 2/signal transducer and activator of transcription 3 signaling pathway. Yang, Y. et al. 2018. Oncol Lett. 16: 6515-6521.
819	30431140	Reactive oxygen species induced by icaritin promote DNA strand breaks and apoptosis in human cervical cancer cells. Chen, X. et al. 2018. Oncol. Rep.
820	30446901	Lower expression of Bax predicts poor clinical outcome in patients with glioma after curative resection and radiotherapy/chemotherapy. Wang, PG. et al. 2018. J. Neurooncol.
821	30456590	Epigallocatechin gallate suppresses premature senescence of preadipocytes by inhibition of PI3K/Akt/mTOR pathway and induces senescent cell death by regulation of Bax/Bcl-2 pathway. Kumar, R. et al. 2018. Biogerontology.
822	30465897	Ganoderma tsugae induced ROS-independent apoptosis and cytoprotective autophagy in human chronic myeloid leukemia cells. Hseu, Y.C. et al. 2019. Food and chemical toxicology : an international journal published for the British Industrial Biological Research Association. 124: 30-44.
823	30478761	Melatonin Rescue Oxidative Stress-Mediated Neuroinflammation/ Neurodegeneration and Memory Impairment in Scopolamine-Induced Amnesia Mice Model. Muhammad, T. et al. 2018. J Neuroimmune Pharmacol. 79: 1948-54.
824	30483133	N-myristoylation of Antimicrobial Peptide CM4 Enhances Its Anticancer Activity by Interacting With Cell Membrane and Targeting Mitochondria in Breast Cancer Cells. Li, C. et al. 2018. Front Pharmacol. 9: 1297.
825	30518922	Ferritin heavy subunit enhances apoptosis of non-small cell lung cancer cells through modulation of miR-125b/p53 axis. Biamonte, F. et al. 2018. Cell Death Dis. 9: 1174.
826	30528266	Zinc and p53 disrupt mitochondrial binding of HK2 by phosphorylating VDAC1. Xue, YN. et al. 2019. Exp. Cell Res. 374: 249-258.
827	30537467	A new human pyridinium metabolite of furosemide, inhibitor of mitochondrial complex I, is a candidate inducer of neurodegeneration. Laurencé, C. et al. 2019. Biochem. Pharmacol. 160: 14-23.
828	30543781	A new pyrrole based small molecule from Tinospora cordifolia induces apoptosis in MDA-MB-231 breast cancer cells via ROS mediated mitochondrial damage and restoration of p53 activity. Rashmi, KC. et al. 2018. Chem. Biol. Interact. 299: 120-130.
829	30549344	Role and regulation of proapoptotic Bax in oral squamous cell carcinoma and drug resistance. Alam, M. et al. 2018. Head Neck.
830	30590389	Cardiomyocyte-specific loss of RMP causes myocardial dysfunction and heart failure. Zhang, J. et al. 2018. Cardiovasc. Res.
831	30618732	Neuroprotective Effect of Quercetin Against the Detrimental Effects of LPS in the Adult Mouse Brain. Khan, A. et al. 2018. Front Pharmacol. 9: 1383.
832	30628717	Introduction of exogenous wild-type p53 mediates the regulation of oncoprotein 18/stathmin signaling via nuclear factor-κB in non-small cell lung cancer NCI-H1299 cells. Chen, S. et al. 2019. Oncol. Rep. 41: 2051-2059.
833	30633472	Protective action of N-acetylcysteine on sperm quality in cyclophosphamide-induced testicular toxicity in male Wistar rats. JBRA Assist Reprod. 23: 83-90.
834	30645642	LRRK2 kinase plays a critical role in manganese-induced inflammation and apoptosis in microglia. Kim, J. et al. 2019. PLoS ONE. 14: e0210248.
835	30664148	Protective roles of isoastilbin against Alzheimer's disease via Nrf2-mediated antioxidation and anti-apoptosis. Yu, H. et al. 2019. Int. J. Mol. Med.
836	30675309	Effects of Beclin 1 overexpression on aggressive phenotypes of colon cancer cells. Zhang, MY. et al. 2019. Oncol Lett. 17: 2441-2450.
837	30684467	Activation of microglia synergistically enhances neurodegeneration caused by MPP+ in human SH-SY5Y cells. Peng, Z. et al. 2019. Eur. J. Pharmacol.
838	30742328	GABA-containing compound gammapyrone protects against brain impairments in Alzheimer's disease model male rats and prevents mitochondrial dysfunction in cell culture. Pilipenko, V. et al. 2019. J. Neurosci. Res.
839	30744126	KIFC1 Inhibitor CW069 Induces Apoptosis and Reverses Resistance to Docetaxel in Prostate Cancer. Sekino, Y. et al. 2019. J Clin Med. 8:
840	30756299	Hesperetin Confers Neuroprotection by Regulating Nrf2/TLR4/NF-κB Signaling in an Aβ Mouse Model. Ikram, M. et al. 2019. Mol. Neurobiol.
841	30768982	Identification of microRNA-mRNA networks involved in cisplatin-induced renal tubular epithelial cells injury. Wu, J. et al. 2019. Eur. J. Pharmacol.
842	30783919	Propolis and Its Combination with Boric Acid Protect Against Ischemia/Reperfusion-Induced Acute Kidney Injury by Inhibiting Oxidative Stress, Inflammation, DNA Damage, and Apoptosis in Rats. Geyikoglu, F. et al. 2019. Biol Trace Elem Res.
843	30787609	An investigation on the cytotoxicity and caspase-mediated apoptotic effect of biologically synthesized gold nanoparticles using Cardiospermum halicacabum on AGS gastric carcinoma cells. Li, C. et al. 2019. Int J Nanomedicine. 14: 951-962.
844	30790227	Clinical significance of metabolism-related biomarkers in non-Hodgkin lymphoma - MCT1 as potential target in diffuse large B cell lymphoma. Afonso, J. et al. 2019. Cell Oncol (Dordr).
845	30794076	Glycyrrhizic acid renders robust neuroprotection in rodent model of vascular dementia by controlling oxidative stress and curtailing cytochrome-c release. Sathyamoorthy, Y. et al. 2019. Nutr Neurosci. 1-16.
846	30805592	MiRNA-206 inhibits hepatocellular carcinoma cell proliferation and migration but promotes apoptosis by modulating cMET expression. Wang, Y. et al. 2019. Acta Biochim. Biophys. Sin. (Shanghai).
847	30806285	Mangiferin Attenuated Diethynitrosamine-Induced Hepatocellular Carcinoma in Sprague-Dawley Rats via Alteration of Oxidative Stress and Apoptotic Pathway. 2019. J. Environ. Pathol. Toxicol. Oncol. 38: 1-12.
848	30816477	Deoxypodophyllotoxin inhibits cell viability and invasion by blocking the PI3K/Akt signaling pathway in human glioblastoma cells. Wang, W. et al. 2019. Oncol. Rep.
849	30817944	Auriculasin sensitizes primary prostate cancer cells to TRAIL-mediated apoptosis through up-regulation of the DR5-dependent pathway. Cho, HD. et al. 2019. Food Chem. Toxicol.
850	30832463	Gadd45b Acts as Neuroprotective Effector in Global Ischemia-Induced Neuronal Death. Int Neurourol J. 23: S11-21.
851	30834631	Pongapin and Karanjin, furanoflavanoids of Pongamia pinnata, induce G2/M arrest and apoptosis in cervical cancer cells by differential reactive oxygen species modulation, DNA damage, and nuclear factor kappa-light-chain-enhancer of activated B cell signaling. Roy, R. et al. 2019. Phytother Res.
852	30856390	Indirubin-3'-monoxime prevents aberrant activation of GSK-3β/NF-κB and alleviates high fat-high fructose induced Aβ-aggregation, gliosis and apoptosis in mice brain. Sathiya Priya, C. et al. 2019. Int. Immunopharmacol. 70: 396-407.
853	30864677	Effects of miR-103a-3p on the autophagy and apoptosis of cardiomyocytes by regulating Atg5. Zhang, C. et al. 2019. Int. J. Mol. Med.
854	30880336	The antioxidant alpha lipoic acid attenuates intermittent hypoxia-related renal injury in a mouse model of sleep apnea. Abuyassin, B. et al. 2019. Sleep.
855	30884890	Hesperetin, a Citrus Flavonoid, Attenuates LPS-Induced Neuroinflammation, Apoptosis and Memory Impairments by Modulating TLR4/NF-κB Signaling. Muhammad, T. et al. 2019. Nutrients. 11:
856	30905858	Rapamycin-induced autophagy decreases Myf5 and MyoD proteins in C2C12 myoblast cells. Yoo, YM. et al. 2019. Toxicol In Vitro.
857	30906480	Myricetin attenuates the severity of seizures and neuroapoptosis in pentylenetetrazole kindled mice by regulating the of BDNF-TrkB signaling pathway and modulating matrix metalloproteinase-9 and GABAA. Sun, ZQ. et al. 2019. Exp Ther Med. 17: 3083-3091.
858	30909499	Growth and Proliferation of Renal Cell Carcinoma Cells Is Blocked by Low Curcumin Concentrations Combined with Visible Light Irradiation. Rutz, J. et al. 2019. Int J Mol Sci. 20:
859	30912136	Loss of MCL1 function sensitizes the MDA-MB-231 breast cancer cells to rh-TRAIL by increasing DR4 levels. De Blasio, A. et al. 2019. J. Cell. Physiol.
860	30923410	The HDAC Inhibitor, SAHA, Combined with Cisplatin Synergistically Induces Apoptosis in Alpha-fetoprotein-producing Hepatoid Adenocarcinoma Cells. Kyaw, MTH. et al. 2019. Acta Histochem Cytochem. 52: 1-8.
861	30942390	Development of finasteride polymer microspheres for systemic application in androgenic alopecia. Kim, JH. et al. 2019. Int. J. Mol. Med.
862	30946848	PPAR-γ agonist GL516 reduces oxidative stress and apoptosis occurrence in a rat astrocyte cell line. Giampietro, L. et al. 2019. Neurochem. Int. 126: 239-245.
863	30965178	Biochemical and molecular responses of cyprinids in two Mediterranean lacustrine ecosystems: Opportunities for ecological assessment and biomonitoring. Kaloyianni, M. et al. 2019. Aquat. Toxicol. 211: 105-115.
864	30974965	Scutellaria Radix Promotes Apoptosis in Non-Small Cell Lung Cancer Cells via Induction of AMPK-Dependent Autophagy. Kim, HI. et al. 2019. Am. J. Chin. Med. 47: 691-705.
865	31051448	Anticancer activity of green synthesised AgNPs from Cymbopogon citratus (LG) against lung carcinoma cell line A549. Chen, Z. et al. 2019. IET Nanobiotechnol. 13: 178-182.
866	31068208	Anti-EMT properties of CoQ0 attributed to PI3K/AKT/NFKB/MMP-9 signaling pathway through ROS-mediated apoptosis Yang, et al. 2019. Journal of Experimental & Clinical Cancer Research. 38:
867	31081049	Icariin enhances the chemosensitivity of cisplatin-resistant ovarian cancer cells by suppressing autophagy via activation of the AKT/mTOR/ATG5 pathway. Int. J. Oncol. 54: 1933-1942.
868	31082581	Fluorene-9-bisphenol exposure induces cytotoxicity in mouse oocytes and causes ovarian damage. Jia, Z. et al. 2019. Ecotoxicol. Environ. Saf. 180: 168-178.
869	31090463	The Role of Melatonin in Oxidative Stress, DNA Damage, Apoptosis and Angiogenesis in Fetal Eye under Preeclampsia and Melatonin Deficiency Stress. 2019. Curr. Eye Res. 1-13.
870	31091792	Caffeine Modulates Cadmium-Induced Oxidative Stress, Neuroinflammation, and Cognitive Impairments by Regulating Nrf-2/HO-1 In Vivo and In Vitro. Khan, A. et al. 2019. J Clin Med. 8:
871	31093684	Acylated ghrelin prevents doxorubicin-induced cardiac intrinsic cell death and fibrosis in rats by restoring IL-6/JAK2/STAT3 signaling pathway and inhibition of STAT1. 2019. Naunyn Schmiedebergs Arch. Pharmacol. 392: 1151-1168.
872	31096703	Natural Dietary Supplementation of Curcumin Protects Mice Brains against Ethanol-Induced Oxidative Stress-Mediated Neurodegeneration and Memory Impairment via Nrf2/TLR4/RAGE Signaling. Ikram, M. et al. 2019. Nutrients. 11:
873	31100273	Protective effect of morin on doxorubicin-induced hepatorenal toxicity in rats. Chem. Biol. Interact. 308: 89-100.
874	31117242	MST1 Regulates Neuronal Cell Death via JNK/Casp3 Signaling Pathway in HFD Mouse Brain and HT22 Cells. 2019. Int J Mol Sci. 20:
875	31145852	Comparative and Mechanistic Study on the Anticancer Activity of Quinacrine-Based Silver and Gold Hybrid Nanoparticles in Head and Neck Cancer. Mol. Pharm. 16: 3011-3023.
876	31159596	Rabdosia rubescens Linn: green synthesis of gold nanoparticles and their anticancer effects against human lung cancer cells A549. 2019. Artif Cells Nanomed Biotechnol. 47: 2171-2178.
877	31161672	Centaurea cyanus extracted 13-O-acetylsolstitialin A decrease Bax/Bcl-2 ratio and expression of cyclin D1/Cdk-4 to induce apoptosis and cell cycle arrest in MCF-7 and MDA-MB-231 breast cancer cell lines. Keyvanloo Shahrestanaki, M. et al. 2019. J. Cell. Biochem.
878	31165450	Protective effects of zingerone on cisplatin-induced nephrotoxicity in female rats. Environ Sci Pollut Res Int. 26: 22562-22574.
879	31210842	Active Constituent in the Ethyl Acetate Extract Fraction of Terminalia bellirica Fruit Exhibits Antioxidation, Antifibrosis, and Proapoptosis Capabilities In Vitro. 2019. Oxid Med Cell Longev. 2019: 5176090.
880	31220110	Green synthesis of silver nanoparticles via Cynara scolymus leaf extracts: The characterization, anticancer potential with photodynamic therapy in MCF7 cells. Erdogan, O. et al. 2019. PLoS ONE. 14: e0216496.
881	31237956	Neuronal Ca2+ -dependent activator protein 1 (NCDAP1) induces neuronal cell death by activating p53 pathway following traumatic brain injury. J. Neurochem. 151: 795-809.
882	31239669	Apoptotic effect of green synthesized gold nanoparticles from Curcuma wenyujin extract against human renal cell carcinoma A498 cells. 2019. Int J Nanomedicine. 14: 4091-4103.
883	31251961	Mitochondrial signaling in inflammation-induced depressive behavior in female and male rats: The role of glucocorticoid receptor. Brain Res. Bull. 150: 317-327.
884	31252023	CDK2 and Bcl-xL inhibitory mechanisms by docking simulations and anti-tumor activity from piperine enriched supercritical extract. Food Chem. Toxicol. 132: 110644.
885	31257554	Targeting ROS overgeneration by N-benzyl-2-nitro-1-imidazole-acetamide as a potential therapeutic reposition approach for cancer therapy. 2019. Invest New Drugs.
886	31278615	FOLFIRI-Mediated Toxicity in Human Aortic Smooth Muscle Cells and Possible Amelioration with Curcumin and Quercetin. Güçlü, O. et al. 2019. Cardiovasc. Toxicol.
887	31284779	Protective Effects of Trimetazidine in Retarding Selenite-Induced Lens Opacification. 2019. Curr. Eye Res. 1-12.
888	31289560	Novel imidazo[1,2-a]pyridine inhibits AKT/mTOR pathway and induces cell cycle arrest and apoptosis in melanoma and cervical cancer cells. Aliwaini, S. et al. 2019. Oncol Lett. 18: 830-837.
889	31293635	J Cancer. 3323-3332.

890	31296660	Temporal and differential regulation of KAISO-controlled transcription by phosphorylated and acetylated p53 highlights a crucial regulatory role of apoptosis. 2019. J. Biol. Chem. 294: 12957-12974.
891	31302853	Synthesis, molecular docking and evaluation of novel sulfonyl hydrazones as anticancer agents and COX-2 inhibitors. Mol. Divers.
892	31309410	Investigating the Proteomic Profile of HT-29 Colon Cancer Cells After Lactobacillus kefiri SGL 13 Exposure Using the SWATH Method. Brandi, J. et al. 2019. J. Am. Soc. Mass Spectrom.
893	31314163	Lipid peroxidation increases hydrogen peroxide permeability leading to cell death in cancer cell lines that lack mtDNA. 2019. Cancer Sci. 110: 2856-2866.
894	31318086	Ethanolic extract of leaf of Dillenia pentagyna reduces in-vitro cell migration and induces intrinsic pathway of apoptosis via downregulation of NF-κβ in human NSCLC A549 cells. De, D. et al. 2019. J. Cell. Biochem.
895	31324205	Effect of transcutaneous electrical acupoint stimulation on protecting against radiotherapy- induced ovarian damage in mice. 2019. J Ovarian Res. 12: 65.
896	31338440	Simulated microgravity induces nuclear translocation of Bax and BCL-2 in glial cultured C6 cells. 2019. Heliyon. 5: e01798.
897	31339234	Oridonin induces Mdm2-p60 to promote p53-mediated apoptosis and cell cycle arrest in neuroblastoma. 2019. Cancer Med. 8: 5313-5326.
898	31344333	The neuroprotective effects of micronized PEA (PEA-m) formulation on diabetic peripheral neuropathy in mice. 2019. FASEB J. fj201900538R.
899	31355678	Downregulation of UBE2T can enhance the radiosensitivity of osteosarcoma in vitro and in vivo. Epigenomics. 11: 1283-1305.
900	31360627	Dentin Matrix Protein 1 Compensates for Lack of Osteopontin in Regulating Odontoblastlike Cell Differentiation after Tooth Injury in Mice. J Endod.
901	31363375	Effect of miR-140-5p on the regulation of proliferation and apoptosis in NSCLC and its underlying mechanism. 2019. Exp Ther Med. 18: 1350-1356.
902	31423257	CDS-1548 induces apoptosis in HeLa cells by activating caspase 3. 2019. Oncol Lett. 18: 1881-1887.
903	31432981	Is the non-metastatic, locally advanced colon adenocarinoma a distinct biological tumor variant? A study based on pathological evaluation, immunohistochemical panel and survival. Rev Col Bras Cir. 46: e20192098.
904	31485651	Sam68 mediates high glucose-induced podocyte apoptosis through modulation of Bax/Bcl-2. 2019. Mol Med Rep. 20: 3728-3734.
905	31488109	Solid state fermentation process with Aspergillus kawachii enhances the cancer-suppressive potential of silkworm larva in hepatocellular carcinoma cells. 2019. BMC Complement Altern Med. 19: 241.
906	31504707	Arabinoxylan rice bran (MGN-3/Biobran) enhances radiotherapy in animals bearing Ehrlich ascites carcinomaθ. 2019. J. Radiat. Res.
907	31507330	The role of low density lipoprotein receptor-related protein 11 as a tumor promoter in cervical cancer. 2019. Cancer Manag Res. 11: 8081-8093.
908	31530851	Renoprotective effects of a novel cMet agonistic antibody on kidney fibrosis. 2019. Sci Rep. 9: 13495.
909	31538224	Ethanol extract of Gynura bicolor (GB) protects against UVB-induced photodamage of skin by inhibiting P53-mediated Bcl-2/BAX/Caspase-3 apoptosis pathway. 2019. Arch. Dermatol. Res.
910	31545447	Synchronous co-expression of Id-1 and nuclear NF-κB p65 promotes cervical cancer progression and malignancy, and is associated with a poor prognosis and chemosensitivity. 2019. Oncol. Rep.
911	31604113	Lupiwighteone induces caspase-dependent and -independent apoptosis on human breast cancer cells via inhibiting PI3K/Akt/mTOR pathway. 2019. Food Chem. Toxicol. 110863.
912	31614639	Allopurinol Prevents the Lipogenic Response Induced by an Acute Oral Fructose Challenge in Short-Term Fructose Fed Rats. García-Arroyo, FE. et al. Biomolecules. 9:
913	31620822	Protective effect of chrysin on cyclophosphamide-induced hepatotoxicity and nephrotoxicity via the inhibition of oxidative stress, inflammation, and apoptosis. Naunyn Schmiedebergs Arch. Pharmacol.
914	31628385	Lignan enriched fraction (LRF) of Phyllanthus amarus promotes apoptotic cell death in human cervical cancer cells in vitro. Sci Rep. 9: 14950.
915	31657930	CREB Signaling Mediates Dose-dependent Radiation Response in the Murine Hippocampus Two Years after Total Body Exposure. J. Proteome Res.
916	31669390	Using CETSA assay and a mathematical model to reveal dual Bcl-2/Mcl-1 inhibition and on-target mechanism for ABT-199 and S1. Eur J Pharm Sci. 105105.
917	31683688	Kinesin Eg5 Targeting Inhibitors as a New Strategy for Gastric Adenocarcinoma Treatment. Molecules. 24:
918	31690718	Ablation of RIP3 protects from dopaminergic neurodegeneration in experimental Parkinson's disease. Dionísio, PA. et al. Cell Death Dis. 10: 840.
919	31717470	Caffeine May Abrogate LPS-Induced Oxidative Stress and Neuroinflammation by Regulating Nrf2/TLR4 in Adult Mouse Brains. Biomolecules. 9:
920	31758295	Maackia amurensis agglutinin induces apoptosis in cultured drug resistant human non-small cell lung cancer cells. Glycoconj. J.
921	31768967	PARP inhibitor Olaparib Enhances the Apoptotic Potentiality of Curcumin by Increasing the DNA Damage in Oral Cancer Cells through Inhibition of BER Cascade. Pathol. Oncol. Res.
922	31773909	Liraglutide Lowers Body Weight Set Point in DIO Rats and its Relationship with Hypothalamic Microglia Activation. Obesity (Silver Spring).
923	31778727	Tanshinone IIA ameliorates cognitive deficits by inhibiting endoplasmic reticulum stress-induced apoptosis in APP/PS1 transgenic mice. Neurochem. Int. 104610.
924	31785241	Tlr4 participates in the responses of markers of apoptosis, inflammation, and ER stress to different acute exercise intensities in mice hearts. Life Sci. 240: 117107.
925	31786496	Investigating the protective actions of D-pinitol against arsenic-induced toxicity in PC12 cells and the underlying mechanism. Environ. Toxicol. Pharmacol. 74: 103302.
926	31791842	BRC-mediated RNAi targeting of USE1 inhibits tumor growth in vitro and in vivo. Biomaterials. 119630.
927	31848848	Di(2-ethylhexyl) phthalate inhibits glutathione regeneration and dehydrogenases of the pentose phosphate pathway on human colon carcinoma cells. Cell Stress Chaperones.
928	31853201	Oxymatrine Inhibits the Proliferation and Invasion of Breast Cancer Cells via the PI3K Pathway. Cancer Manag Res. 11: 10499-10508
929	31865501	Progesterone Protects Prefrontal Cortex in Rat Model of Permanent Bilateral Common Carotid Occlusion via Progesterone Receptors and Akt/Erk/eNOS. Cell. Mol. Neurobiol.
930	31880441	Biodegradation-Mediated Enzymatic Activity-Tunable Molybdenum Oxide Nanourchins for Tumor-Specific Cascade Catalytic Therapy. J. Am. Chem. Soc.
931	31884568	Sevoflurane Induces Learning and Memory Impairment in Young Mice Through a Reduction in Neuronal Glucose Transporter 3. Cell. Mol. Neurobiol.
932	31894259	CCR4-NOT transcription complex subunit 2 regulates TRAIL sensitivity in non-small-cell lung cancer cells via the STAT3 pathway. Int. J. Mol. Med.
933	31894331	Sirtuin 3 induces apoptosis and necroptosis by regulating mutant p53 expression in small-cell lung cancer. Oncol. Rep. 43: 591-600.
934	31905895	Octyl Gallate Induces Pancreatic Ductal Adenocarcinoma Cell Apoptosis and Suppresses Endothelial-Mesenchymal Transition-Promoted M2-Macrophages, HSP90α Secretion, and Tumor Growth. Cells. 9:
935	31922246	MicroRNA-3651 promotes colorectal cancer cell proliferation through directly repressing T-box transcription factor 1. Int. J. Mol. Med.
936	31929494	The Nephroprotective Effect of TNF Receptor-Associated Factor 6 (TRAF6) Blockade on LPS-Induced Acute Renal Injury Through the Inhibition if Inflammation and Oxidative Stress. Med. Sci. Monit. e919698.
937	31938312	Int J Clin Exp Pathol. 2053-2063.
938	31947843	Anthocyanins from Hibiscus syriacus L. Inhibit Oxidative Stress-Mediated Apoptosis by Activating the Nrf2/HO-1 Signaling Pathway. Antioxidants (Basel). 9:
939	31960439	Wnt/β-catenin-mediated heat exposure inhibits intestinal epithelial cell proliferation and stem cell expansion through endoplasmic reticulum stress. J. Cell. Physiol.
940	31963225	Oregano Feed Supplementation Affects Glycoconjugates Production in Swine Gut. Animals (Basel). 10:
941	31982469	Quercetin Reduces Ischemic Brain Injury by Preventing Ischemia-induced Decreases in the Neuronal Calcium Sensor Protein Hippocalcin. Neuroscience. 430: 47-62.
942	31989645	Mitochondria-targeted antioxidant mito-TEMPO alleviate oxidative stress induced by antimycin A in human mesenchymal stem cells. J. Cell. Physiol.
943	31991882	Overcoming Resistance to Platinum-Based Drugs in Ovarian Cancer by Salinomycin and Its Derivatives-An In Vitro Study. Molecules. 25:
944	32001620	The transcription factor REST up-regulates tyrosine hydroxylase and antiapoptotic genes and protects dopaminergic neurons against manganese toxicity. J. Biol. Chem.
945	32018227	Baicalin alleviates benign prostate hyperplasia through androgen-dependent apoptosis. Aging (Albany NY). 12: 2142-2155.
946	32020222	Inhibitory mechanism of muscone in liver cancer involves the induction of apoptosis and autophagy. Oncol. Rep.
947	32046101	Adaptation of Mitochondrial Substrate Flux in a Mouse Model of Nonalcoholic Fatty Liver Disease. Staňková, P. et al. Int J Mol Sci. 21:
948	32050753	Inhibitory Role of TRIP-Br1/XIAP in Necroptosis under Nutrient/Serum Starvation. Mol. Cells.
949	32075054	Sanggenol L Induces Apoptosis and Cell Cycle Arrest via Activation of p53 and Suppression of PI3K/Akt/mTOR Signaling in Human Prostate Cancer Cells. Nutrients. 12:
950	32084496	Sanggenol L promotes apoptotic cell death in melanoma skin cancer cells through activation of caspase cascades and apoptosis-inducing factor. Food Chem. Toxicol. 111221.
951	32102534	Apoptosis Induced by Prednisolone Occurs without Altering the Promoter Methylation of BAX and BCL-2 Genes in Acute Lymphoblastic Leukemia Cells CCRF-CEM. Asian Pac. J. Cancer Prev. 21: 523-529.
952	32113683	Ablation of glutaredoxin 1 promotes pulmonary angiogenesis and alveolar formation in hyperoxia-injured lungs by modifying HIF-1α stability and inhibiting the NF-κB pathway. Biochem. Biophys. Res. Commun.
953	32132927	The Effects of Calorie Restriction and Exercise on Age-Related Alterations in Corpus Cavernosum. Front Physiol. 11: 45.
954	32150824	EPA is More Effective than DHA to Improve Depression-Like Behavior, Glia Cell Dysfunction and Hippcampal Apoptosis Signaling in a Chronic Stress-Induced Rat Model of Depression. Int J Mol Sci. 21:
955	32150979	KCP10043F Represses the Proliferation of Human Non-Small Cell Lung Cancer Cells by Caspase-Mediated Apoptosis via STAT3 Inactivation. J Clin Med. 9:
956	32167384	Calprotectin (S100A8/S100A9)-induced cytotoxicity and apoptosis in human gastric cancer AGS cells: Alteration in expression levels of Bax, Bcl-2, and ERK2. Hum Exp Toxicol. 960327120909530.
957	32182943	Oral Administration of Alpha Linoleic Acid Rescues Aβ-Induced Glia-Mediated Neuroinflammation and Cognitive Dysfunction in C57BL/6N Mice. Cells. 9:
958	32185206	Improvement of Inflammation through Antioxidant Pathway of Gardeniae Fructus 50% EtOH Extract (GE) from Acute Reflux Esophagitis Rats. Biomed Res Int. 2020: 4826176.
959	32200504	Mechanism of inositol-requiring enzyme 1-alpha inhibition in endoplasmic reticulum stress and apoptosis in ovarian cancer cells. J Cell Commun Signal.
960	32201518	MicroRNA-19a-3p regulates cell growth through modulation of the PIK3IP1-AKT pathway in hepatocellular carcinoma. J Cancer. 11: 2476-2484.
961	32230772	Cyanidin Chloride Induces Apoptosis by Inhibiting NF-κB Signaling through Activation of Nrf2 in Colorectal Cancer Cells. Antioxidants (Basel). 9:
962	32231136	Proteome Analysis in a Mammalian Cell line Reveals that PLK2 is Involved in Avian Metapneumovirus Type C (aMPV/C)-Induced Apoptosis. Viruses. 12:
963	32244920	Hericium Erinaceus Prevents DEHP-Induced Mitochondrial Dysfunction and Apoptosis in PC12 Cells. Amara, I. et al. Int J Mol Sci. 21:
964	32255378	MBTPS2 exacerbates albuminuria in streptozotocin-induced type I diabetic nephropathy by promoting endoplasmic reticulum stress-mediated renal damage. Arch. Physiol. Biochem. 1-8.
965	32277521	Exosomes derived from RPE cells under oxidative stress mediate inflammation and apoptosis of normal RPE cells through Apaf1/caspase-9 axis. J. Cell. Biochem.
966	32278271	Chemopreventive role of arabinoxylan rice bran, MGN-3/Biobran, on liver carcinogenesis in rats. Biomed. Pharmacother. 126: 110064.
967	32293462	HSP90 inhibitor 17AAG attenuates sevoflurane-induced neurotoxicity in rats and human neuroglioma cells via induction of HSP70. J Transl Med. 18: 166.
968	32302678	Calpain mediated expansion of CD4+ cytotoxic T cells in rodent models of Parkinson's disease. Exp. Neurol. 330: 113315.
969	32338270	Lysine inhibits apoptosis in satellite cells to govern skeletal muscle growth via the JAK2-STAT3 pathway. Food Funct.
970	32345289	A novel kefir product (PFT) inhibits Ehrlich ascites carcinoma in mice via induction of apoptosis and immunomodulation. BMC Complement Med Ther. 20: 127.
971	32356241	Synthetic antiprotozoal thiazolide drug induced apoptosis in colorectal cancer cells: implications of IL-6/JAK2/STAT3 and p53/caspases-dependent signaling pathways based on molecular docking and in vitro study. Mol. Cell. Biochem.
972	32357291	Calbindin-D9k is a Novel Risk Gene for Neurodegenerative Disease. Cell. Physiol. Biochem. 54: 438-456.
973	32375410	Biomolecules.
974	32407635	Quinacrine based gold hybrid nanoparticle caused apoptosis through modulating replication fork in oral cancer stem cells. Mol. Pharm.
975	32429243	Ultramicronized Palmitoylethanolamide and Paracetamol, a New Association to Relieve Hyperalgesia and Pain in a Sciatic Nerve Injury Model in Rat. Int J Mol Sci. 21:
976	32443623	Antioxidant and Neuroprotective Effects Induced by Cannabidiol and Cannabigerol in Rat CTX-TNA2 Astrocytes and Isolated Cortexes. Int J Mol Sci. 21:
977	32459053	BRD2 induces drug resistance through activation of the RasGRP1/Ras/ERK signaling pathway in adult T-cell lymphoblastic lymphoma. Cancer Commun (Lond).
978	32460595	Recombinant Human Elafin Ameliorates Chronic Hyperoxia-Induced Lung Injury by Inhibiting Nuclear Factor-Kappa B Signaling in Neonatal Mice. J. Interferon Cytokine Res.
979	32484605	Computational and in vitro characterization of ICY-5: A potential candidate promoting mitochondrial apoptosis via the c-MET and STAT3 pathways. J. Cell. Physiol.
980	32500380	Effect of di(2-ethylhexyl) phthalate on Nrf2-regulated glutathione homeostasis in mouse kidney. Cell Stress Chaperones.
981	32504794	The small molecule P7C3-A20 exerts neuroprotective effects in a hypoxic-ischemic encephalopathy model via activation of PI3K/AKT/GSK3β signaling. Neuroscience.
982	32509140	tBHQ Induces a Hormetic Response That Protects L6 Myoblasts against the Toxic Effect of Palmitate. Posadas-Rodríguez, P. et al. 2020. Oxid Med Cell Longev. 2020: 3123268.
983	32566008	Propofol induces ROS-mediated intrinsic apoptosis and migration in triple-negative breast cancer cells. Oncol Lett. 810-816.
984	32569592	Cyclosporine a induces testicular injury via mitochondrial apoptotic pathway by regulation of mir-34a and sirt-1 in male rats: The rescue effect of curcumin. Chem. Biol. Interact. 109180.
985	32578930	Cabazitaxel exhibits more favorable molecular changes compared to other taxanes in androgen-independent prostate cancer cells. J. Biochem. Mol. Toxicol. e22542.
986	32622253	Targeted co-delivery of curcumin and doxorubicin by citric acid functionalized Poly (ε-caprolactone) based micelle in MDA-MB-231 cell. Colloids Surf B Biointerfaces. 111225.
987	32623575	Gamma linolenic acid suppresses hypoxia-induced proliferation and invasion of non-small cell lung cancer cells by inhibition of HIF1α. Genes Genomics.
988	32627020	Tubeimoside-1 induces apoptosis in human glioma U251 cells by suppressing PI3K/Akt-mediated signaling pathways. Mol Med Rep.
989	32635491	Protective Effects of SPA0355, a Thiourea Analogue, Against Lipopolysaccharide-Induced Acute Kidney Injury in Mice. Antioxidants (Basel).
990	32638843	Changrui enema inhibits inflammation-induced angiogenesis in acute radiation proctitis by regulating NF-κB and VEGF. Acta Cir Bras. e202000502.
991	32647007	Cathepsin A contributes to left ventricular remodeling by degrading extracellular superoxide dismutase in mice. J. Biol. Chem.
992	32663669	Bioorg. Chem. 104063.
993	32682001	MNDA controls the expression of MCL-1 and BCL-2 in chronic lymphocytic leukemia cells. Exp. Hematol.
994	32703236	Yin Yang 1 is critical for mid-hindbrain neuroepithelium development and involved in cerebellar agenesis. Mol Brain. 104.
995	32706154	Circular RNA hsa_circRNA_102209 promotes the growth and metastasis of colorectal cancer through miR-761-mediated Ras and Rab interactor 1 signaling. Cancer Med.
996	32729104	Role of ABT888, a Novel Poly(ADP-Ribose) Polymerase (PARP) Inhibitor in Countering Autophagy and Apoptotic Processes Associated to Spinal Cord Injury. Casili, G. et al. 2020. Molecular neurobiology. 57: 4394-4407.
997	32733640	PI3K/Akt and ERK1/2 Signalling Are Involved in Quercetin-Mediated Neuroprotection against Copper-Induced Injury. Oxid Med Cell Longev. 9834742.
998	32740515	Anti-TNF-α Therapy Exerts Intestinal Anti-inflammatory and Anti-apoptotic Effects After Massive Bowel Resection in a Rat. Sukhotnik, I. et al. 2021. J Pediatr Gastroenterol Nutr. 72: 49-55.
999	32774810	Iran J Basic Med Sci. 886-893.
1000	32788939	Oncol Lett. 52.
1001	32790676	PLoS ONE. e0236879.
1002	32791955	Hypertens Pregnancy. 1-15.
1003	32800223	Ecotoxicol. Environ. Saf. 110940.
1004	32814167	The PI3Kα inhibitor DFX24 suppresses tumor growth and metastasis in non-small cell lung cancer via ERK inhibition and EPHB6 reactivation. Fan, YH. et al. 2020. Pharmacol Res. 160: 105147.
1005	32830792	Med. Sci. Monit. e924922.
1006	32850324	Front Oncol. 1141.
1007	32850788	Front Cell Dev Biol. 622.
1008	32851512	Genes Genomics.
1009	32872275	Nutrients.
1010	32885602	J. Cell. Mol. Med.
1011	32922229	Dose Response. 1559325820951332.
1012	32929329	PARK2 promotes mitochondrial pathway of apoptosis and antimicrotubule drugs chemosensitivity via degradation of phospho-BCL-2. Chen, H. et al. 2020. Theranostics. 10: 9984-10000.
1013	32938995	Sci Rep. 15193.
1014	32945468	Int. J. Oncol.
1015	32950951	Phytomedicine. 153319.
1016	32961113	Arch. Biochem. Biophys. 108591.
1017	32963743	The effect of low dose amphetamine in rotenone-induced toxicity in a mice model of Parkinson's disease. Abdel-Salam, OME. et al. 2020. Iran J Basic Med Sci. 23: 1207-1217.
1018	32991881	Life Sci. 118494.
1019	32994687	5272-5286.
1020	32998768	34.
1021	33011221	Life Sci. 118518.
1022	33016225	Bioengineered. 1058-1070.
1023	33019979	Biochem Biophys Res Commun.
1024	33036367	Molecules.
1025	33039525	Neuroscience.
1026	33059700	J Neuroinflammation. 303.
1027	33062640	Front Mol Biosci. 216.
1028	33093476	Cell Death Dis. 900.
1029	33099657	Cell Biol Toxicol.
1030	33111211	Mol Cell Biochem.
1031	33116119	Oncogenesis. 97.
1032	33117773	Front Bioeng Biotechnol. 908.
1033	33127495	Food Chem Toxicol. 111828.
1034	33143356	Int J Mol Sci.
1035	33179090	Mol Med Rep.
1036	33204684	Biomed Res Int. 1928410.
1037	33209128	Exp Ther Med. 284.
1038	33219891	Cell biology and toxicology.
1039	33220236	Exp Eye Res. 108360.
1040	33237503	Genes Genomics.
1041	33260329	Nutrients.
1042	33268463	Diabetes.
1043	33281967	Oncol Lett. 56.
1044	33288747	Cell Death Dis. 1040.
1045	33294128	4-tert-Octylphenol Exposure Disrupts Brain Development and Subsequent Motor, Cognition, Social, and Behavioral Functions. Tran, DN. et al. 2020. Oxid Med Cell Longev. 2020: 8875604.
1046	33299329	Onco Targets Ther. 12457-12469.
1047	33300068	Mol Med Rep.
1048	33301842	Food Chem Toxicol. 111918.
1049	33320730	The different doses of radiation therapy-induced damage to the ovarian environment in rats. Onder, GO. et al. 2021. Int J Radiat Biol. 97: 367-375.
1050	33325270	Hum Exp Toxicol. 960327120979014.
1051	33338554	Food Chem Toxicol. 111924.
1052	33355139	Sci Adv.
1053	33359245	Life Sci. 118939.
1054	33375627	Cancers (Basel).
1055	33383360	Evaluating the in vitro therapeutic effects of human amniotic mesenchymal stromal cells on MiaPaca2 pancreatic cancer cells using 2D and 3D cell culture model. Rahmani, Z. et al. 2021. Tissue Cell. 68: 101479.
1056	33388284	Chlorpromazine, an antipsychotic agent, induces G2/M phase arrest and apoptosis via regulation of the PI3K/AKT/mTOR-mediated autophagy pathways in human oral cancer. Jhou, AJ. et al. 2021. Biochem Pharmacol. 184: 114403.
1057	33419140	Cells.
1058	33425768	Front Oncol. 613679.
1059	33510839	D-Limonene Alleviates Acute Kidney Injury Following Gentamicin Administration in Rats: Role of NF-κB Pathway, Mitochondrial Apoptosis, Oxidative Stress, and PCNA. Babaeenezhad, E. et al. 2021. Oxid Med Cell Longev. 2021: 6670007.
1060	33539914	LC3B/p62-mediated mitophagy protects A549 cells from resveratrol-induced apoptosis. Zheng, J. et al. 2021. Life Sci. 271: 119139.
1061	33559503	Renoprotective effects of prazosin on ischemia-reperfusion injury in rats. Rahimi, MM. et al. 2021. Hum Exp Toxicol. 960327121993224.
1062	33562817	Neurotoxic Effect of Flavonol Myricetin in the Presence of Excess Copper. Sadžak, A. et al. 2021. Molecules. 26:
1063	33566162	Cell death induction (extrinsic versus intrinsic apoptotic pathway) by intestinal ischemia-reperfusion injury in rats is time-depended. Ben-Shahar, Y. et al. 2021. Pediatr Surg Int. 37: 369-376.
1064	33621077	Troxerutin Improves Dextran Sulfate Sodium-Induced Ulcerative Colitis in Mice. Wang, X. et al. 2021. J Agric Food Chem. 69: 2729-2744.
1065	33628290	Satureja khuzistanica Jamzad essential oil prevents doxorubicin-induced apoptosis via extrinsic and intrinsic mitochondrial pathways. Seyedan, AA. et al. 2020. Res Pharm Sci. 15: 481-490.
1066	33651486	Fish Oil Diet during Pre-mating, Gestation, and Lactation in Adult Offspring Rats on Cancer Cachexia Prevention. de Oliveira, SCP. et al. 2021. Mol Nutr Food Res. e2000863.
1067	33668893	Synthesis, Cytotoxicity and Anti-Proliferative Activity Against AGS Cells of New 3(2H)-Pyridazinone Derivatives Endowed with a Piperazinyl Linker. Alagöz, MA. et al. 2021. Pharmaceuticals (Basel). 14:
1068	33716741	Fisetin Rescues the Mice Brains Against D-Galactose-Induced Oxidative Stress, Neuroinflammation and Memory Impairment. Ahmad, S. et al. 2021. Front Pharmacol. 12: 612078.
1069	33718370	Combined Administration of Metformin and Atorvastatin Attenuates Diabetic Cardiomyopathy by Inhibiting Inflammation, Apoptosis, and Oxidative Stress in Type 2 Diabetic Mice. Jia, W. et al. 2021. Front Cell Dev Biol. 9: 634900.
1070	33721789	Characterization of rat liver bud-derived cells. Silva, DRS. et al. 2021. Tissue Cell. 71: 101510.
1071	33738673	The molecular mechanism of a novel derivative of BTO-956 induced apoptosis in human myelomonocytic lymphoma cells. Li, YL. et al. 2021. Apoptosis. 26: 219-231.
1072	33741083	TBK1 inhibitor exerts anti-proliferative effect on glioblastoma multiforme cells. Scuderi, S.A. et al. 2021. Oncology research.
1073	33760202	SIRT6-specific inhibitor OSS-128167 exacerbates diabetic cardiomyopathy by aggravating inflammation and oxidative stress. Huang, Y. et al. 2021. Mol Med Rep. 23:
1074	33761993	The combination of G-CSF and AMD3100 mobilizes bone marrow-derived stem cells to protect against cisplatin-induced acute kidney injury in mice. Chen, Z. et al. 2021. Stem Cell Res Ther. 12: 209.
1075	33774859	SS-31 protect retinal pigment epithelial cells from H2 O2 -induced cell injury by reducing apoptosis. Bai, J. et al. 2021. Clin Exp Pharmacol Physiol.
1076	33785814	The effectiveness of Rutin for prevention of surgical induced endometriosis development in a rat model. Talebi, H. et al. 2021. Sci Rep. 11: 7180.
1077	33786275	Protective effect of pregabalin on the brain tissue of diabetic rats. Demir, CF. et al. 2021. Diabetol Int. 12: 207-216.
1078	33801889	The Mechanisms of the Anti-Inflammatory and Anti-Apoptotic Effects of Omega-3 Polyunsaturated Fatty Acids during Methotrexate-Induced Intestinal Damage in Cell Line and in a Rat Model. Koppelmann, T. et al. 2021. Nutrients. 13:
1079	33807213	High ROS Production by Celecoxib and Enhanced Sensitivity for Death Ligand-Induced Apoptosis in Cutaneous SCC Cell Lines. Zhu, J. et al. 2021. Int J Mol Sci. 22:
1080	33850525	Effects of Shenfu Qiangxin Drink on H2O2-induced oxidative stress, inflammation and apoptosis in neonatal rat cardiomyocytes and possible underlying mechanisms. Zhang, S. et al. 2021. Exp Ther Med. 21: 553.
1081	33859778	SM22α Loss Contributes to Apoptosis of Vascular Smooth Muscle Cells via Macrophage-Derived circRasGEF1B. Lv, P. et al. 2021. Oxid Med Cell Longev. 2021: 5564884.
1082	33859925	MicroRNA-520a Suppresses Pathogenesis and Progression of Non-Small-Cell Lung Cancer through Targeting the RRM2/Wnt Axis. Xie, Y. et al. 2021. Anal Cell Pathol (Amst). 2021: 9652420.
1083	33868469	Siomycin A induces reactive oxygen species-mediated cytotoxicity in ovarian cancer cells. Shao, X. et al. 2021. Oncol Lett. 21: 431.
1084	33877580	Mst1/2-ALK promotes NLRP3 inflammasome activation and cell apoptosis during Listeria monocytogenes infection. Gao, A. et al. 2021. J Microbiol. 59: 681-692.
1085	33895481	Knockdown of circ-UQCRC2 ameliorated lipopolysaccharide-induced injury in MRC-5 cells by the miR-326/PDCD4/NF-κB pathway. Zhou, G. et al. 2021. Int Immunopharmacol. 97: 107633.
1086	33915380	Apigenin-loaded galactose tailored PLGA nanoparticles: A possible strategy for liver targeting to treat hepatocellular carcinoma. Ganguly, S. et al. 2021. Colloids Surf B Biointerfaces. 204: 111778.
1087	33920212	Nicotinamide Ameliorates Amyloid Beta-Induced Oxidative Stress-Mediated Neuroinflammation and Neurodegeneration in Adult Mouse Brain. Rehman, IU. et al. 2021. Biomedicines. 9:
1088	33924360	The Methyl Ester of 2-Cyano-3,12-Dioxooleana-1,9-Dien-28-Oic Acid Reduces Endometrial Lesions Development by Modulating the NFkB and Nrf2 Pathways. Siracusa, R. et al. 2021. International journal of molecular sciences. 22:
1089	33925007	Mitochondrial Transplantation Modulates Inflammation and Apoptosis, Alleviating Tendinopathy Both In Vivo and In Vitro. Lee, JM. et al. 2021. Antioxidants (Basel). 10:
1090	33929897	Deficiency of ataxia-telangiectasia mutated kinase modulates functional and biochemical parameters of the heart in response to Western-type diet. Wingard, MC. et al. 2021. Am J Physiol Heart Circ Physiol. 320: H2324-H2338.
1091	33940179	Exosomes as a potential messenger unit during heterochronic parabiosis for amelioration of Huntington's disease. Lee, M. et al. 2021. Neurobiol Dis. 105374.
1092	33946346	Oxyresveratrol Ameliorates Dextran Sulfate Sodium-Induced Colitis in Rats by Suppressing Inflammation. Yeom, J. et al. 2021. Molecules. 26:
1093	33953856	Comparative study of gavage and intraperitoneal administration of gamma-oryzanol in alleviation/attenuation in a rat animal model of renal ischemia/reperfusion-induced injury. Bagheri, Y. et al. 2021. Iran J Basic Med Sci. 24: 175-183.
1094	33962019	Melatonin prevents blood-retinal barrier breakdown and mitochondrial dysfunction in high glucose and hypoxia-induced in vitro diabetic macular edema model. Doğanlar, ZB. et al. 2021. Toxicol In Vitro. 75: 105191.
1095	33968717	miRNA-193a-3p Regulates the AKT2 Pathway to Inhibit the Growth and Promote the Apoptosis of Glioma Cells by Targeting ALKBH5. Cui, Y. et al. 2021. Front Oncol. 11: 600451.
1096	33974882	Effects of trans-resveratrol on type 1 diabetes-induced up-regulation of apoptosis and mitogen-activated protein kinase signaling in retinal pigment epithelium of Dark Agouti rats. Al-Hussaini, H. et al. 2021. Eur J Pharmacol. 904: 174167.
1097	33986643	Unbiased Label-Free Quantitative Proteomics of Cells Expressing Amyotrophic Lateral Sclerosis (ALS) Mutations in CCNF Reveals Activation of the Apoptosis Pathway: A Workflow to Screen Pathogenic Gene Mutations. Cheng, F. et al. 2021. Front Mol Neurosci. 14: 627740.
1098	33994365	Biological Effect of Silver-modified Nanostructured Titanium Dioxide in Cancer. Lagopati, N. et al. Cancer Genomics Proteomics. 18: 425-439.
1099	34025784	Arsenic trioxide-induced cell apoptosis and cell cycle arrest are potentiated by 1,25-dihydroxyvitamin D3 in human leukemia K562 cells. Zhang, YL. et al. 2021. Oncol Lett. 22: 509.
1100	34033197	lncRNA PVT1 promotes cetuximab resistance of head and neck squamous cell carcinoma cells by inhibiting miR-124-3p. Yang, S. et al. 2021. Head Neck. 43: 2712-2723.
1101	34036388	Guan Xin Dan Shen formulation protects db/db mice against diabetic cardiomyopathy via activation of Nrf2 signaling. Zhang, B. et al. 2021. Mol Med Rep. 24:
1102	34036504	Protective role of yeast beta-glucan on lead acetate-induced hepatic and reproductive toxicity in rats. Tatli Seven, P. et al. 2021. Environ Sci Pollut Res Int.
1103	34038756	Arsenic induces autophagy-dependent apoptosis via Akt inactivation and AMPK activation signaling pathways leading to neuronal cell death. Fu, SC. et al. 2021. Neurotoxicology. 85: 133-144.
1104	34064310	Hidrox® and Endometriosis: Biochemical Evaluation of Oxidative Stress and Pain. Cordaro, M. et al. 2021. Antioxidants (Basel, Switzerland). 10:
1105	34064854	Autophagy and Mitophagy Promotion in a Rat Model of Endometriosis. Siracusa, R. et al. 2021. International journal of molecular sciences. 22:
1106	34068585	Downregulation of ATM and BRCA1 Predicts Poor Outcome in Head and Neck Cancer: Implications for ATM-Targeted Therapy. Wang, YC. et al. 2021. J Pers Med. 11:
1107	34068924	Hidrox® Counteracts Cyclophosphamide-Induced Male Infertility through NRF2 Pathways in a Mouse Model. Fusco, R. et al. 2021. Antioxidants (Basel, Switzerland). 10:
1108	34083581	The curcumin analog (PAC) suppressed cell survival and induced apoptosis and autophagy in oral cancer cells. Semlali, A. et al. 2021. Sci Rep. 11: 11701.
1109	34094602	The Role of Dectin-1-Mediated M1 Macrophage Polarization in Cerebral Ischemia-Reperfusion Injury. Zongyun, C. et al. 2021. Emerg Med Int. 2021: 6697271.
1110	34097239	Oral Administration of Silibinin Ameliorates Cognitive Deficits of Parkinson's Disease Mouse Model by Restoring Mitochondrial Disorders in Hippocampus. Liu, X. et al. 2021. Neurochem Res. 46: 2317-2332.
1111	34103635	PB01 suppresses radio-resistance by regulating ATR signaling in human non-small-cell lung cancer cells. Kim, TW. et al. 2021. Sci Rep. 11: 12093.
1112	34119916	Combination of Gemcitabine and Thymosin alpha 1 exhibit a better anti-tumor effect on nasal natural killer/T-cell lymphoma. Chen, M. et al. 2021. Int Immunopharmacol. 98: 107829.
1113	34121978	MicroRNA-30c-2-3p regulates ER stress and induces apoptosis in ovarian cancer cells underlying ER stress. Rezghi Barez, S. et al. 2021. EXCLI J. 20: 922-934.

1114	34128234	Evaluating the effect of secretome of human amniotic mesenchymal stromal cells on apoptosis induction and epithelial-mesenchymal transition inhibition in LNCaP prostate cancer cells based on 2D and 3D cell culture models. Safari, F. et al. 2021. Cell Biochem Funct. 39: 813-820.
1115	34131514	The combination of lactoferrin and linolenic acid inhibits colorectal tumor growth through activating AMPK/JNK-related apoptosis pathway. Yao, Q. et al. 2021. PeerJ. 9: e11072.
1116	34146926	Effects of tetrahydrocannabinols on human oral cancer cell proliferation, apoptosis, autophagy, oxidative stress, and DNA damage. Semlali, A. et al. 2021. Arch Oral Biol. 129: 105200.
1117	34158560	Anethole induces anti-oral cancer activity by triggering apoptosis, autophagy and oxidative stress and by modulation of multiple signaling pathways. Contant, C. et al. 2021. Sci Rep. 11: 13087.
1118	34199098	Western Diet Decreases the Liver Mitochondrial Oxidative Flux of Succinate: Insight from a Murine NAFLD Model. Staňková, P. et al. 2021. Int J Mol Sci. 22:
1119	34199629	Hericium erinaceus and Coriolus versicolor Modulate Molecular and Biochemical Changes after Traumatic Brain Injury. D'Amico, R. et al. 2021. Antioxidants (Basel, Switzerland). 10:
1120	34202665	Palmitoylethanolamide/Baicalein Regulates the Androgen Receptor Signaling and NF-κB/Nrf2 Pathways in Benign Prostatic Hyperplasia. D'Amico, R. et al. 2021. Antioxidants (Basel, Switzerland). 10:
1121	34206129	Regulation of Inflammatory and Proliferative Pathways by Fotemustine and Dexamethasone in Endometriosis. Genovese, T. et al. 2021. International journal of molecular sciences. 22:
1122	34207896	Effects of Dietary Polyphenols from Olive Mill Waste Waters on Inflammatory and Apoptotic Effectors in Rabbit Ovary. Maranesi, M. et al. 2021. Animals (Basel). 11:
1123	34216418	L-Carnosine Protects Against Deoxynivalenol-induced Oxidative Stress in Intestinal Stem Cells by Regulating the Keap1/Nrf2 Signaling Pathway. Zhou, JY. et al. 2021. Mol Nutr Food Res. e2100406.
1124	34217710	Dual effects of atorvastatin on angiogenesis pathways in the differentiation of mesenchymal stem cells. Khalighfard, S. et al. 2021. Eur J Pharmacol. 174281.
1125	34245104	The inhibition of mammalian target of rapamycin (mTOR) in improving inflammatory response after traumatic brain injury. Campolo, M. et al. 2021. Journal of cellular and molecular medicine. 25: 7855-7866.
1126	34251942	Design, synthesis, HER2 inhibition and anticancer evaluation of new substituted 1,5-dihydro-4,1-benzoxazepines. Cruz-López, O. et al. 2021. J Enzyme Inhib Med Chem. 36: 1553-1563.
1127	34269965	Protective Effects of Estrogen via Nanoparticle Delivery to Attenuate Myelin Loss and Neuronal Death after Spinal Cord Injury. Haque, A. et al. 2021. Neurochem Res.
1128	34298658	KYP-2047, an Inhibitor of Prolyl-Oligopeptidase, Reduces GlioBlastoma Proliferation through Angiogenesis and Apoptosis Modulation. Scuderi, S.A. et al. 2021. Cancers. 13:
1129	34305900	Protective Role of Kynurenine 3-Monooxygenase in Allograft Rejection and Tubular Injury in Kidney Transplantation. Lassiter, R. et al. 2021. Front Immunol. 12: 671025.
1130	34306137	Berbamine Inhibits Cell Proliferation and Migration and Induces Cell Death of Lung Cancer Cells via Regulating c-Maf, PI3K/Akt, and MDM2-P53 Pathways. Liu, L. et al. 2021. Evid Based Complement Alternat Med. 2021: 5517143.
1131	34313039	Melatonin mitigates the adverse effect of hypoxia during myocardial differentiation in mouse embryonic stem cells. Lee, JH. et al. 2021. J Vet Sci. 22: e54.
1132	34346000	LncRNA XIST knockdown alleviates LPS-induced acute lung injury by inactivation of XIST/miR-132-3p/MAPK14 pathway : XIST promotes ALI via miR-132-3p/MAPK14 axis. Li, C. et al. 2021. Mol Cell Biochem.
1133	34355433	Trilobolide-6-O-isobutyrate suppresses hepatocellular carcinoma tumorigenesis through inhibition of IL-6/STAT3 signaling pathway. Zhou, XQ. et al. 2021. Phytother Res.
1134	34356835	Poly (ADP-Ribose) Polymerase Inhibitor, ABT888, Improved Cisplatin Effect in Human Oral Cell Carcinoma. Paterniti, I. et al. 2021. Biomedicines. 9:
1135	34360708	Atrazine Inhalation Causes Neuroinflammation, Apoptosis and Accelerating Brain Aging. Genovese, T. et al. 2021. International journal of molecular sciences. 22:
1136	34366426	Comparison of the Effects of Intraperitoneal Injection with Carbon Tetrachloride on Acute Liver Toxicity in Male and Female Kunming Mice. Yang, H. et al. 2021. Med Sci Monit. 27: e931427.
1137	34373755	Discovery of Small Molecule Bak Activator for Lung Cancer Therapy. Park, D. et al. 2021. Theranostics. 11: 8500-8516.
1138	34382727	miR-221 confers lapatinib resistance by negatively regulating p27kip1 in HER2-positive breast cancer. Huynh, TK. et al. 2021. Cancer Sci. 112: 4234-4245.
1139	34384848	The toxicity of Aspidosperma subincanum to MCF7 cells is related to modulation of oxidative status and proinflammatory pathways. Santos, AF. et al. 2021. J Ethnopharmacol. 114512.
1140	34400143	Mangiferin mitigates di-(2-ethylhexyl) phthalate-induced testicular injury in rats by modulating oxidative stress-mediated signals, inflammatory cascades, apoptotic pathways, and steroidogenesis. Awny, MM. et al. 2021. Arch Biochem Biophys. 108982.
1141	34417154	The in-vivo assessment of Turkish propolis and its nano form on testicular damage induced by cisplatin. Tatli Seven, P. et al. 2021. J Integr Med.
1142	34434268	Inhibition of autophagy promotes human RSV NS1-induced inflammation and apoptosis in vitro. Han, B. et al. 2021. Exp Ther Med. 22: 1054.
1143	34434495	The protective role of prolyl oligopeptidase (POP) inhibition in acute lung injury induced by intestinal ischemia-reperfusion. Casili, G. et al. 2021. Oncotarget. 12: 1663-1676.
1144	34439112	Genetic Suppressor Element 1 (GSE1) Promotes the Oncogenic and Recurrent Phenotypes of Castration-Resistant Prostate Cancer by Targeting Tumor-Associated Calcium Signal Transducer 2 (TACSTD2). Bamodu, OA. et al. 2021. Cancers (Basel). 13:
1145	34440193	Nanonutraceuticals: Anti-Cancer Activity and Improved Safety of Chemotherapy by Costunolide and Its Nanoformulation against Colon and Breast Cancer. El-Far, AH. et al. 2021. Biomedicines. 9:
1146	34440569	Isolation, Characterization and Neuroprotective Activity of Folecitin: An In Vivo Study. Farooq, U. et al. 2021. Life (Basel). 11:
1147	34441662	Effects of a New Combination of Natural Extracts on Glaucoma-Related Retinal Degeneration. Molinari, C. et al. 2021. Foods (Basel, Switzerland). 10:
1148	34443427	Enhancement of Anticancer Potential of Pterostilbene Derivative by Chalcone Hybridization. Tang, KW. et al. 2021. Molecules. 26:
1149	34452517	The Microvillar and Solitary Chemosensory Cells as the Novel Targets of Infection of SARS-CoV-2 in Syrian Golden Hamsters. Seo, JS. et al. 2021. Viruses. 13:
1150	34463003	EIF4A3-mediated hsa_circ_0088088 promotes the carcinogenesis of breast cancer by sponging miR-135-5p. Liu, Q. et al. 2021. J Biochem Mol Toxicol. e22909.
1151	34471052	Apoptosis Inducing Activity of Rhinacanthin-C in Doxorubicin-Resistant Breast Cancer MCF-7 Cells. Chaisit, S. et al. 2021. Biol Pharm Bull. 44: 1239-1246.
1152	34476501	Atrial natriuretic peptide protects vertebral endplate chondrocytes against H2O2-induced apoptosis and oxidative stress through activation of the Nrf2/HO-1 signaling pathway. He, F. et al. 2021. Mol Med Rep. 24:
1153	34490481	Phillygenin inhibits the inflammation and apoptosis of pulmonary epithelial cells by activating PPARγ signaling via downregulation of MMP8. Lin, Y. et al. 2021. Mol Med Rep. 24:
1154	34490824	Thymoquinone and Costunolide Induce Apoptosis of Both Proliferative and Doxorubicin-Induced-Senescent Colon and Breast Cancer Cells. El-Far, AH. et al. Integr Cancer Ther. 20: 15347354211035450.
1155	34502488	Deciphering the Potential Neuroprotective Effects of Luteolin against Aβ1-42-Induced Alzheimer's Disease. Ahmad, S. et al. 2021. Int J Mol Sci. 22:
1156	34532880	New chromanone derivatives containing thiazoles: Synthesis and antitumor activity evaluation on A549 lung cancer cell line. Yurttaş, L. et al. 2021. Drug Dev Res.
1157	34536204	Selenomethionine Alleviates Intestinal Ischemia-Reperfusion Injury in Mice Through the Bax-Caspase Pathway. Liu, Z. et al. 2021. Biol Trace Elem Res.
1158	34538671	Dihydrotanshinone I inhibits the growth of hepatoma cells by direct inhibition of Src. Jiang, XL. et al. 2021. Phytomedicine. 153705.
1159	34552038	miR-21 regulates ischemic neuronal injury via the p53/Bcl-2/Bax signaling pathway. Yan, H. et al. 2021. Aging (Albany NY). 13: 22242-22255.
1160	34555716	Chronic developmental exposure to low-dose ([C8mim][PF6]) induces neurotoxicity and behavioural abnormalities in rats. Su, X. et al. 2021. Ecotoxicol Environ Saf. 225: 112806.
1161	34570918	Resolvin D1 prevents cadmium chloride-induced memory loss and hippocampal damage in rats by activation/upregulation of PTEN-induced suppression of PI3K/Akt/mTOR signaling pathway. Shati, AA. et al. 2022. Clin Exp Pharmacol Physiol. 49: 275-290.
1162	34571925	Alpha-Linolenic Acid Impedes Cadmium-Induced Oxidative Stress, Neuroinflammation, and Neurodegeneration in Mouse Brain. Alam, SI. et al. 2021. Cells. 10:
1163	34573124	Fisetin Protects HaCaT Human Keratinocytes from Fine Particulate Matter (PM2.5)-Induced Oxidative Stress and Apoptosis by Inhibiting the Endoplasmic Reticulum Stress Response. Molagoda, IMN. et al. 2021. Antioxidants (Basel). 10:
1164	34588186	Imeglimin ameliorates β-cell apoptosis by modulating the endoplasmic reticulum homeostasis pathway. Li, J. et al. 2021. Diabetes.
1165	34632965	Schisandrin B Attenuates Colitis-Associated Colorectal Cancer through SIRT1 Linked SMURF2 Signaling. Pu, Z. et al. 2021. Am J Chin Med. 1-17.
1166	34656609	The potential neuroprotective effect of diosmin in rotenone-induced model of Parkinson's disease in rats. Habib, CN. et al. 2021. Eur J Pharmacol. 174573.
1167	34664776	Circ-SNAP47 (hsa_circ_0016760) and miR-625-5p are regulators of WEE1 in regulation of chemoresistance, growth and invasion of DDP-tolerant NSCLC cells via ceRNA pathway. Zou, C. et al. 2021. Environ Toxicol.
1168	34666484	Ganoderterpene A, a New Triterpenoid from Ganoderma lucidum, Attenuates LPS-Induced Inflammation and Apoptosis via Suppressing MAPK and TLR-4/NF-κB Pathways in BV-2 Cells. Kou, RW. et al. 2021. J Agric Food Chem.
1169	34677776	Stress-induced miRNAs isolated from wheat have a unique therapeutic potential in ultraviolet-stressed human keratinocyte cells. Arda, H. et al. 2022. Environ Sci Pollut Res Int. 29: 17977-17996.
1170	34678260	Involvement of mitogen-activated protein kinase (MAPK)-activated protein kinase 2 (MK2) in endothelial dysfunction associated with pulmonary hypertension. Shafiq, M. et al. 2021. Life Sci. 120075.
1171	34679649	Crucial Role of Reactive Oxygen Species (ROS) for the Proapoptotic Effects of Indirubin Derivatives in Cutaneous SCC Cells. Zhu, J. et al. 2021. Antioxidants (Basel). 10:
1172	34680471	Inhibition of Prolyl Oligopeptidase Prevents Consequences of Reperfusion following Intestinal Ischemia. Filippone, A. et al. 2021. Biomedicines. 9:
1173	34681768	NLRP3 Inflammasome Inhibitor BAY-117082 Reduces Oral Squamous Cell Carcinoma Progression. Scuderi, S.A. et al. 2021. International journal of molecular sciences. 22:
1174	34685699	Cycloastragenol, a Triterpenoid Saponin, Regulates Oxidative Stress, Neurotrophic Dysfunctions, Neuroinflammation and Apoptotic Cell Death in Neurodegenerative Conditions. Ikram, M. et al. 2021. Cells. 10:
1175	34699320	E2F transcription factor 1 is involved in the phenotypic modulation of esophageal squamous cell carcinoma cells via microRNA-375. Li, P. et al. 2021. Bioengineered.
1176	34712707	1-MNA Ameliorates High Fat Diet-Induced Heart Injury by Upregulating Nrf2 Expression and Inhibiting NF-κB in vivo and in vitro. Song, Z. et al. 2021. Front Cardiovasc Med. 8: 721814.
1177	34713437	Calycosin ameliorates atherosclerosis by enhancing autophagy via regulating the interaction between KLF2 and MLKL in apoE-/- mice. Ma, C. et al. 2021. Br J Pharmacol.
1178	34718338	MicroRNA-106a regulates autophagy-related cell death and EMT by targeting TP53INP1 in lung cancer with bone metastasis. Han, L. et al. 2021. Cell Death Dis. 12: 1037.
1179	34729850	Gigantol inhibits proliferation and enhanced oxidative stress-mediated apoptosis through modulating of Wnt/β-catenin signaling pathway in HeLa cells. Kang, H. et al. 2021. J Biochem Mol Toxicol. e22944.
1180	34734014	The expression of heat shock protein A12B (HSPA12B) in non-Hodgkin's lymphomas. Huang, Y. et al. 2021. Ann Transl Med. 9: 1462.
1181	34768769	Human Islet Amyloid Polypeptide Overexpression in INS-1E Cells Influences Amylin Oligomerization under ER Stress and Oxidative Stress. Yoo, YM. et al. 2021. Int J Mol Sci. 22:
1182	34770968	Effect of Resveratrol Treatment on Human Pancreatic Cancer Cells through Alterations of Bcl-2 Family Members. Ratajczak, K. et al. 2021. Molecules. 26:
1183	34773171	Contribution of endoplasmic reticulum stress, MAPK and PI3K/Akt pathways to the apoptotic death induced by a penicillin derivative in melanoma cells. Bellizzi, Y. et al. 2022. Apoptosis. 27: 34-48.
1184	34775889	Exosomes derived from microRNA-129-5p-modified tumor cells selectively enhanced suppressive effect in malignant behaviors of homologous colon cancer cells. Ji, Y. et al. 2021. Bioengineered.
1185	34796352	Enhanced supply of methionine regulates protein synthesis in bovine mammary epithelial cells under hyperthermia condition. Zhou, J. et al. 2021. J Anim Sci Technol. 63: 1126-1141.
1186	34859534	Ginsenoside-Rg1 attenuates sepsis-induced cardiac dysfunction by modulating mitochondrial damage via the P2X7 receptor-mediated Akt/GSK-3β signaling pathway. Liu, Z. et al. 2021. J Biochem Mol Toxicol. e22885.
1187	34884933	Prenatal Exposure to Triclocarban Impairs ESR1 Signaling and Disrupts Epigenetic Status in Sex-Specific Ways as Well as Dysregulates the Expression of Neurogenesis- and Neurotransmitter-Related Genes in the Postnatal Mouse Brain. Wnuk, A. et al. 2021. Int J Mol Sci. 22:
1188	34890588	Protective role of irbesartan against cyclophosphamide-induced testicular damage in rats via up-regulating PPAR-γ signaling and ameliorating NF-κB/NLRP3/IL-18 inflammatory axis. Abu-Risha, SE. et al. 2021. Life Sci. 120218.
1189	34917264	Beneficial effect of KYP-2047, a propyl-oligopeptidase inhibitor, on oral squamous cell carcinoma. Scuderi, SA. et al. 2021. Oncotarget. 12: 2459-2473.
1190	34932399	Delta-tocotrienol enhances the anti-tumor effects of interferon alpha through reactive oxygen species and Erk/MAPK signaling pathways in hepatocellular carcinoma cells. Lucci, A. et al. 2022. Can J Physiol Pharmacol. 100: 453-463.
1191	34935054	Dichloromethane fractions of Calystegiasoldanella induce Sphase arrest and apoptosis in HT29 human colorectal cancer cells. Kim, IH. et al. 2022. Mol Med Rep. 25:
1192	34943034	Powdered Green Tea (Matcha) Attenuates the Cognitive Dysfunction via the Regulation of Systemic Inflammation in Chronic PM2.5-Exposed BALB/c Mice. Kim, JM. et al. 2021. Antioxidants (Basel). 10:
1193	34943060	l-Arginine Alleviates LPS-Induced Oxidative Stress and Apoptosis via Activating SIRT1-AKT-Nrf2 and SIRT1-FOXO3a Signaling Pathways in C2C12 Myotube Cells. Zhao, Y. et al. 2021. Antioxidants (Basel). 10:
1194	34983556	Conjugation of VEGFR1/R2-targeting peptide with gold nanoparticles to enhance antiangiogenic and antitumoral activity. Zanjanchi, P. et al. 2022. J Nanobiotechnology. 20: 7.
1195	34983563	Circular RNA ciRs-126 promotes hypoxia/reoxygenation cardiac injury possibly through miR-21. Tan, C. et al. 2022. Thromb J. 20: 2.
1196	34987583	HIF-1α Is Associated with Resistance to Hypoxia-Induced Apoptosis in Ameloblastoma. Valladares, KJP. et al. 2021. Int J Dent. 2021: 3060375.
1197	34992682	Aberrant GIMAP2 expression affects oral squamous cell carcinoma progression by promoting cell cycle and inhibiting apoptosis. Komatsu, M. et al. 2022. Oncol Lett. 23: 49.
1198	34998873	Preparation and characterization of 2-deacetyl-3-O-sulfo-heparosan and its antitumor effects via the fibroblast growth factor receptor pathway. Zha, Z. et al. 2022. Int J Biol Macromol. 201: 47-58.
1199	35008928	Protective Effect of Gamma Aminobutyric Acid against Aggravation of Renal Injury Caused by High Salt Intake in Cisplatin-Induced Nephrotoxicity. Lee, H. et al. 2022. Int J Mol Sci. 23:
1200	35012534	Monocarboxylate transporter functions and neuroprotective effects of valproic acid in experimental models of amyotrophic lateral sclerosis. Gyawali, A. et al. 2022. J Biomed Sci. 29: 2.
1201	35013118	Neurokinin-1 receptor promotes non-small cell lung cancer progression through transactivation of EGFR. Zhang, XW. et al. 2022. Cell Death Dis. 13: 41.
1202	35051602	Paeoniflorigenone regulates apoptosis, autophagy, and necroptosis to induce anti-cancer bioactivities in human head and neck squamous cell carcinomas. Park, KR. et al. 2022. J Ethnopharmacol. 288: 115000.
1203	35053421	Rhenium Perrhenate (188ReO4) Induced Apoptosis and Reduced Cancerous Phenotype in Liver Cancer Cells. Asadian, S. et al. 2022. Cells. 11:
1204	35059399	Isothiocyanate From Moringa oleifera Seeds Inhibits the Growth and Migration of Renal Cancer Cells by Regulating the PTP1B-dependent Src/Ras/Raf/ERK Signaling Pathway. Xie, J. et al. 2021. Front Cell Dev Biol. 9: 790618.
1205	35060363	Histone deacetylase HDAC2 regulates microRNA-125a expression in neuroblastoma. Liu, D. et al. 2022. Brain Behav. e2401.
1206	35065059	Naringenin affords protection against lipopolysaccharide/D-galactosamine-induced acute liver failure: Role of autophagy. Ahmedy, OA. et al. 2022. Arch Biochem Biophys. 109121.
1207	35083997	Tilapia skin peptides restore cyclophosphamide-induced premature ovarian failure via inhibiting oxidative stress and apoptosis in mice. Zhao, YT. et al. 2022. Food Funct. 13: 1668-1679.
1208	35086056	Podocyte protection by Angptl3 knockout via inhibiting ROS/GRP78 pathway in LPS-induced acute kidney injury. Ma, Y. et al. 2022. Int Immunopharmacol. 105: 108549.
1209	35096564	MED10 Drives the Oncogenicity and Refractory Phenotype of Bladder Urothelial Carcinoma Through the Upregulation of hsa-miR-590. Wu, CC. et al. 2021. Front Oncol. 11: 744937.
1210	35103292	Epigenetic therapy with chidamide alone or combined with 5-azacitidine exerts antitumour effects on acute myeloid leukaemia cells in vitro. Li, Z. et al. 2022. Oncol Rep. 47:
1211	35112410	The nature compound dehydrocrenatidine exerts potent antihepatocellular carcinoma by destroying mitochondrial complexes in vitro and in vivo. Hou, ZL. et al. 2022. Phytother Res. 36: 1353-1371.
1212	35126604	The Potential Hepatoprotective Effect of Paeoniae Radix Alba in Thioacetamide-Induced Acute Liver Injury in Rats. Shin, MR. et al. 2022. Evid Based Complement Alternat Med. 2022: 7904845.
1213	35126806	Empagliflozin Enhances Autophagy, Mitochondrial Biogenesis, and Antioxidant Defense and Ameliorates Renal Ischemia/Reperfusion in Nondiabetic Rats. Ala, M. et al. 2022. Oxid Med Cell Longev. 2022: 1197061.
1214	35134642	Synthetic methodology-enabled discovery of a tunable indole template for COX-1 inhibition and anti-cancer activity. Guerra Faura, G. et al. 2022. Bioorg Med Chem. 57: 116633.
1215	35140838	BMS-470539 Attenuates Oxidative Stress and Neuronal Apoptosis via MC1R/cAMP/PKA/Nurr1 Signaling Pathway in a Neonatal Hypoxic-Ischemic Rat Model. Yu, S. et al. 2022. Oxid Med Cell Longev. 2022: 4054938.
1216	35159285	Identification of Small Molecules Inhibiting Cardiomyocyte Necrosis and Apoptosis by Autophagy Induction and Metabolism Reprogramming. Liu, D. et al. 2022. Cells. 11:
1217	35166983	Neuregulin-1 regulates the conversion of M1/M2 microglia phenotype via ErbB4-dependent inhibition of the NF-κB pathway. Ma, Y. et al. 2022. Mol Biol Rep.
1218	35189318	Effect of Early Exercise on Inflammatory Parameters and Apoptosis in CA1 Area of the Hippocampus Following Cerebral Ischemia-reperfusion in Rats. Dehqanizadeh, B. et al. 2022. Brain Res Bull.
1219	35192847	The new mitochondrial uncoupler BAM15 induces ROS production for treatment of acute myeloid leukemia. Xing Gao, Z. et al. 2022. Biochem Pharmacol. 114948.
1220	35194432	Anticancer Activity of Delphinium semibarbatum Alkaloid Fractions against LNCaP, and DU 145 Human Prostate Cancer Cells through the Intrinsic Apoptotic Pathway. Lotfaliani, M. et al. 2021. Iran J Pharm Res. 20: 107-116.
1221	35202708	Carbon tetrachloride induced mitochondrial division, respiratory chain damage, abnormal intracellular [H+] and apoptosis are due to the activation of 5-HT degradation system in hepatocytes. Zhang, Y. et al. 2022. Toxicol Appl Pharmacol. 439: 115929.
1222	35204143	LDHB Deficiency Promotes Mitochondrial Dysfunction Mediated Oxidative Stress and Neurodegeneration in Adult Mouse Brain. Park, JS. et al. 2022. Antioxidants (Basel). 11:
1223	35207590	The p53-Driven Anticancer Effect of Ribes fasciculatum Extract on AGS Gastric Cancer Cells. Kim, MJ. et al. 2022. Life (Basel). 12:
1224	35216504	Premarin Reduces Neurodegeneration and Promotes Improvement of Function in an Animal Model of Spinal Cord Injury. Haque, A. et al. 2022. Int J Mol Sci. 23:
1225	35216510	The Effects of a Meldonium Pre-Treatment on the Course of the LPS-Induced Sepsis in Rats. Đurašević, S. et al. 2022. Int J Mol Sci. 23:
1226	35217377	Triterpenoids and meroterpenoids from the edible Ganoderma resinaceum and their potential anti-inflammatory, antioxidant and anti-apoptosis activities. Kou, RW. et al. 2022. Bioorg Chem. 121: 105689.
1227	35225400	Effects of bone marrow-derived mesenchymal stem cells on doxorubicin-induced liver injury in rats. Celik Samanci, T. et al. 2022. J Biochem Mol Toxicol. 36: e22985.
1228	35227836	Human umbilical cord mesenchymal stem cell-derived extracellular vesicles loaded with miR-223 ameliorate myocardial infarction through P53/S100A9 axis. Yang, M. et al. 2022. Genomics. 110319.
1229	35255604	EW-7197 Attenuates the Progression of Diabetic Nephropathy in db/db Mice through Suppression of Fibrogenesis and Inflammation. Ha, KB. et al. 2022. Endocrinol Metab (Seoul). 37: 96-111.
1230	35256749	Naturally occurring deamidated triosephosphate isomerase is a promising target for cell-selective therapy in cancer. Enríquez-Flores, S. et al. 2022. Sci Rep. 12: 4028.
1231	35260073	ROR2 increases the chemoresistance of melanoma by regulating p53 and Bcl2-family proteins via ERK hyperactivation. Castro, MV. et al. 2022. Cell Mol Biol Lett. 27: 23.
1232	35263537	Sodium Selenite Modulates IDO1/Kynurenine, TLR4, NF-κB and Bcl2/Bax Pathway and Mitigates Acetic Acid-Induced Colitis in Rat. Ala, M. et al. 2022. Cell Physiol Biochem. 56: 24-35.
1233	35265366	Maslinic Acid Protects against Streptozotocin-Induced Diabetic Retinopathy by Activating Nrf2 and Suppressing NF-κB. Alsabaani, NA. et al. 2022. J Ophthalmol. 2022: 3044202.
1234	35266424	Exogenous glutathione protects against gentamicin-induced acute kidney injury by inhibiting NF-κB pathway, oxidative stress, and apoptosis and regulating PCNA. Babaeenezhad, E. et al. 2022. Drug Chem Toxicol. 1-10.
1235	35267023	Site-Specific Integration of TRAIL in iPSC-Derived Mesenchymal Stem Cells for Targeted Cancer Therapy. Wang, Z. et al. 2022. Stem Cells Transl Med.
1236	35274824	Chloroxylon swietenia (Roxb.) DC induces cell death and apoptosis by down-regulating the NF-κB pathway in MCF-7 breast cancer cells: In vitro and in vivo investigations. Kamble, SS. et al. 2022. Cancer Rep (Hoboken). e1600.
1237	35286011	MicroRNA-455-3p functions as a tumor suppressor by targeting HDAC2 to regulate cell cycle in hepatocellular carcinoma. Ma, F. et al. 2022. Environ Toxicol. 37: 1675-1685.
1238	35299600	Thymax, a gross thymic extract, exerts cell cycle arrest and apoptosis in Ehrlich ascites carcinoma in vivo. Badr El-Din, NK. et al. 2022. Heliyon. 8: e09047.
1239	35302204	Reduced placental size and increased apoptosis are associated with prenatal nicotine exposure in rats. Alzu'bi, A. et al. 2022. Eur Rev Med Pharmacol Sci. 26: 1586-1593.
1240	35332611	Baicalein inhibits cell proliferation and enhances apoptosis in human A549 cells and benzo(a)pyrene-induced pulmonary carcinogenesis in mice. Chandrashekar, N. et al. 2022. J Biochem Mol Toxicol. e23053.
1241	35334874	Yellow Chaste Weed and Its Components, Apigenin and Galangin, Affect Proliferation and Oxidative Stress in Blue Light-Irradiated HaCaT Cells. Park, JY. et al. 2022. Nutrients. 14:
1242	35336740	Leelamine Modulates STAT5 Pathway Causing Both Autophagy and Apoptosis in Chronic Myelogenous Leukemia Cells. Jung, YY. et al. 2022. Biology (Basel). 11:
1243	35340880	Gastrodin attenuates lipopolysaccharide-induced inflammation and oxidative stress, and promotes the osteogenic differentiation of human periodontal ligament stem cells through enhancing sirtuin3 expression. Feng, Q. et al. 2022. Exp Ther Med. 23: 296.
1244	35358852	Redox proteome analysis of auranofin exposed ovarian cancer cells (A2780). Chiappetta, G. et al. 2022. Redox Biol. 52: 102294.
1245	35373507	Chemotherapy impairs skeletal muscle mitochondrial homeostasis in early breast cancer patients. Mallard, J. et al. 2022. J Cachexia Sarcopenia Muscle.
1246	35381197	Filamentous GLS1 promotes ROS-induced apoptosis upon glutamine deprivation via insufficient asparagine synthesis. Jiang, B. et al. 2022. Mol Cell. 82: 1821-1835.e6.
1247	35392232	Glycerophosphoinositol Promotes Apoptosis of Chronic Lymphocytic Leukemia Cells by Enhancing Bax Expression and Activation. Boncompagni, G. et al. 2022. Front Oncol. 12: 835290.
1248	35406088	Consumption of Cashew (Anacardium occidentale L.) Nuts Counteracts Oxidative Stress and Tissue Inflammation in Mild Hyperhomocysteinemia in Rats. D'Amico, R. et al. 2022. Nutrients. 14:
1249	35419170	Cryptotanshinone Protects against PCOS-Induced Damage of Ovarian Tissue via Regulating Oxidative Stress, Mitochondrial Membrane Potential, Inflammation, and Apoptosis via Regulating Ferroptosis. Liu, H. et al. 2022. Oxid Med Cell Longev. 2022: 8011850.
1250	35427636	Puerarin mitigates oxidative injuries, opening of mitochondrial permeability transition pores and pathological damage associated with liver and kidney in Xanthium strumarium-intoxicated rats. Keskin Alkaç, Z. et al. 2022. Toxicon. 213: 13-22.
1251	35444186	Restoration of ER proteostasis attenuates remote apoptotic cell death after spinal cord injury by reducing autophagosome overload. Bisicchia, E. et al. 2022. Cell Death Dis. 13: 381.
1252	35452510	Female Mice are More Resistant to the Mixed-Field (67% Neutron + 33% Gamma) Radiation-Induced Injury in Bone Marrow and Small Intestine than Male Mice due to Sustained Increases in G-CSF and the Bcl-2/Bax Ratio and Lower miR-34a and MAPK Activation. Kiang, JG. et al. 2022. Radiat Res. 198: 120-133.
1253	35456980	Role of Bevacizumab on Vascular Endothelial Growth Factor in Apolipoprotein E Deficient Mice after Traumatic Brain Injury. Genovese, T. et al. 2022. International journal of molecular sciences. 23:
1254	35487267	Targeted delivery of doxorubicin by Thermo/pH-responsive magnetic nanoparticles in a rat model of breast cancer. Pourradi, NMA. et al. 2022. Toxicol Appl Pharmacol. 116036.
1255	35496295	Farrerol Alleviates Myocardial Ischemia/Reperfusion Injury by Targeting Macrophages and NLRP3. Zhou, L. et al. 2022. Front Pharmacol. 13: 879232.
1256	35500641	Potential cardioprotective effect of octreotide via NOXs mitigation, mitochondrial biogenesis and MAPK/Erk1/2/STAT3/NF-kβ pathway attenuation in isoproterenol-induced myocardial infarction in rats. Khalifa, AA. et al. 2022. Eur J Pharmacol. 925: 174978.
1257	35500680	Necroptosis modulation by cisplatin and sunitinib in hepatocellular carcinoma cell line. El-Demiry, SM. et al. 2022. Life Sci. 301: 120594.
1258	35509367	Antitumor Activities of Green Tea by Up-regulation of miR-181a Expression in LNCaP Cells Using 3D Cell Culture Model. Safari, F. et al. Avicenna J Med Biotechnol. 14: 89-94.
1259	35521092	Novel pH-sensitive and biodegradable micelles for the combined delivery of doxorubicin and conferone to induce apoptosis in MDA-MB-231 breast cancer cell line. Rahmani, A. et al. 2020. RSC Adv. 10: 29228-29246.
1260	35526035	SUN11602, a bFGF mimetic, modulated neuroinflammation, apoptosis and calcium-binding proteins in an in vivo model of MPTP-induced nigrostriatal degeneration. Ardizzone, A. et al. 2022. Journal of neuroinflammation. 19: 107.
1261	35527663	Knockdown of EPSTI1 alleviates lipopolysaccharide-induced inflammatory injury through regulation of NF-κB signaling in a cellular pneumonia model. Kari, A. et al. 2022. Allergol Immunopathol (Madr). 50: 106-112.
1262	35545731	Serum-derived extracellular vesicles mediate Smad4 expression through shuttling microRNA-27a in the progression of laryngeal squamous cell carcinoma. Shuang, Y. et al. 2022. Hum Cell. 35: 1084-1099.
1263	35563099	Evaluation of a Gel Containing a Propionibacterium Extract in an In Vivo Model of Wound Healing. M, C. et al. 2022. International journal of molecular sciences. 23:
1264	35566271	Nanoformulation of Polyphenol Curcumin Enhances Cisplatin-Induced Apoptosis in Drug-Resistant MDA-MB-231 Breast Cancer Cells. Karami, P. et al. 2022. Molecules. 27:
1265	35580525	SIAH1 promotes senescence and apoptosis of nucleus pulposus cells to exacerbate disc degeneration through ubiquitinating XIAP. Fang, S. et al. 2022. Tissue Cell. 76: 101820.
1266	35584581	Spiropachysine A suppresses hepatocellular carcinoma proliferation by inducing methuosis in vitro and in vivo. Fang, Y. et al. 2022. Phytomedicine. 102: 154151.
1267	35585092	Enhanced anticancer potency with reduced nephrotoxicity of newly synthesized platin-based complexes compared with cisplatin. Salehi, R. et al. 2022. Sci Rep. 12: 8316.
1268	35598479	Coagulansin-A improves spatial memory in 5xFAD Tg mice by targeting Nrf-2/NF-κB and Bcl-2 pathway. Shal, B. et al. 2022. Int Immunopharmacol. 109: 108860.
1269	35611760	Protective effects of melatonin on female rat ovary treated with nonylphenol. Goktepe, O. et al. 2022. Biotech Histochem. 1-7.
1270	35620406	Citrus unshiu Peel Attenuates Dextran Sulfate Sodium-Induced Ulcerative Colitis in Mice due to Modulation of the PI3K/Akt Signaling Pathway and MAPK and NF-κB. Lee, SH. et al. 2022. Evid Based Complement Alternat Med. 2022: 4041402.
1271	35631686	Anti-Cancerous Potential of Polysaccharides Derived from Wheat Cell Culture. Murtazina, A. et al. 2022. Pharmaceutics. 14:
1272	35639049	Gestational cholestasis induced intrauterine growth restriction through triggering IRE1α-mediated apoptosis of placental trophoblast cells. Gao, XX. et al. 2022. FASEB J. 36: e22388.
1273	35641486	Hepatocyte Bcl-3 protects from death-receptor mediated apoptosis and subsequent acute liver failure. Gehrke, N. et al. 2022. Cell Death Dis. 13: 510.
1274	35658216	Pharmacological mechanism of xanthoangelol underlying Nrf-2/TRPV1 and anti-apoptotic pathway against scopolamine-induced amnesia in mice. Syed, Z. et al. 2022. Biomed Pharmacother. 150: 113073.
1275	35663445	Induction of apoptotic cell death in human bladder cancer cells by ethanol extract of Zanthoxylum schinifolium leaf, through ROS-dependent inactivation of the PI3K/Akt signaling pathway. Park, C. et al. 2022. Nutr Res Pract. 16: 330-343.
1276	35666424	The combined restoration of miR-424-5p and miR-142-3p effectively inhibits MCF-7 breast cancer cell line via modulating apoptosis, proliferation, colony formation, cell cycle and autophagy. Dastmalchi, N. et al. 2022. Mol Biol Rep. 49: 8325-8335.
1277	35677372	Sugiol Suppresses the Proliferation of Human U87 Glioma Cells via Induction of Apoptosis and Cell Cycle Arrest. Alharthy, SA. et al. 2022. Evid Based Complement Alternat Med. 2022: 7658899.
1278	35684123	Oleuropein Counteracts Both the Proliferation and Migration of Intra- and Extragonadal Seminoma Cells. Bossio, S. et al. 2022. Nutrients. 14:
1279	35698771	Impact of imatinib administration on the mouse ovarian follicle count and levels of intra-ovarian proteins related to follicular quality. Kim, SJ. et al. 2022. Clin Exp Reprod Med. 49: 93-100.
1280	35714705	miR-16-5p regulates aerobic glycolysis and tumorigenesis of NSCLC cells via LDH-A/lactate/NF-κB signaling. Arora, S. et al. 2022. Life Sci. 304: 120722.
1281	35722462	Crocus sativus L. Tepal Extract Induces Apoptosis in Human U87 Glioblastoma Cells. Baba, SA. et al. 2022. Biomed Res Int. 2022: 4740246.
1282	35738618	Protective Effects of Gamma-mangostin on Hydrogen Peroxideinduced Cytotoxicity in Human Retinal Pigment Epithelial Cells. Hu, PS. et al. In Vivo. 36: 1676-1683.
1283	35740470	Discovering the Effects of Fisetin on NF-κB/NLRP-3/NRF-2 Molecular Pathways in a Mouse Model of Vascular Dementia Induced by Repeated Bilateral Carotid Occlusion. Cordaro, M. et al. 2022. Biomedicines. 10:
1284	35751681	Synergistic antitumor effect of a penicillin derivative combined with thapsigargin in melanoma cells. Bellizzi, Y. et al. 2022. J Cancer Res Clin Oncol. 148: 3361-3373.
1285	35753508	Selenium nanoparticles impart robust neuroprotection against deltamethrin-induced neurotoxicity in male rats by reversing behavioral alterations, oxidative damage, apoptosis, and neuronal loss. Khalil, HMA. et al. 2022. Neurotoxicology.
1286	35756079	CREB Ameliorates Osteoarthritis Progression Through Regulating Chondrocytes Autophagy via the miR-373/METTL3/TFEB Axis. Zhang, H. et al. 2021. Front Cell Dev Biol. 9: 778941.
1287	35766160	Olmesartan medoxomil self-microemulsifying drug delivery system reverses apoptosis and improves cell adhesion in trinitrobenzene sulfonic acid-induced colitis in rats. Murad, H. et al. 2022. Drug Deliv. 29: 2017-2028.
1288	35773941	Bisphenol A Enhances Apoptosis, Fibrosis, and Biochemical Fluctuations in the Liver of Adult Male Rats with Possible Regression after Recovery. Shehata Amin, MA. et al. 2022. Anat Rec (Hoboken).
1289	35784758	Chinese Medicine, Succinum, Ameliorates Cognitive Impairment of Carotid Artery Ligation Rats and Inhibits Apoptosis of HT22 Hippocampal Cells via Regulation of the GSK3β/β-Catenin Pathway. Wei, C. et al. 2022. Front Pharmacol. 13: 867477.
1290	35785730	GLP-1 mediates the neuroprotective action of crocin against cigarette smoking-induced cognitive disorders via suppressing HMGB1-RAGE/TLR4-NF-κB pathway. Mohammed El Tabaa, M. et al. 2022. Int Immunopharmacol. 110: 108995.
1291	35788494	Keratinocyte-derived S100A9 modulates neutrophil infiltration and affects psoriasis-like skin and joint disease. Mellor, L.F. et al. 2022. Annals of the rheumatic diseases. 81: 1400-8.
1292	35805980	Proscillaridin A Sensitizes Human Colon Cancer Cells to TRAIL-Induced Cell Death. Semba, M. et al. 2022. Int J Mol Sci. 23:
1293	35807356	Protective Effects of Jujubosides on 6-OHDA-Induced Neurotoxicity in SH-SY5Y and SK-N-SH Cells. Chen, CH. et al. 2022. Molecules. 27:
1294	35809662	TRAIL & EGFR affibody dual-display on a protein nanoparticle synergistically suppresses tumor growth. Jun, H. et al. 2022. J Control Release.
1295	35810384	Sildenafil improves radiation-induced oral mucositis by attenuating oxidative stress, NF-κB, ERK and JNK signalling pathways. Ala, M. et al. 2022. J Cell Mol Med.
1296	35853537	miR-495-3p regulates sphingolipid metabolic reprogramming to induce Sphk1/ceramide mediated mitophagy and apoptosis in NSCLC. Arora, S. et al. 2022. Free Radic Biol Med.
1297	35855832	Cirsilineol Inhibits the Proliferation of Human Prostate Cancer Cells by Inducing Reactive Oxygen Species (ROS)-Mediated Apoptosis. Wahab, S. et al. 2022. Evid Based Complement Alternat Med. 2022: 7975664.
1298	35856441	Administration of 4-hexylresorcinol increases p53-mediated transcriptional activity in oral cancer cells with the p53 mutation. Kang, YJ. et al. 2022. Oncol Rep. 48:
1299	35860469	p53: A Regulator of Ferroptosis Induced by Galectin-1 Derived Peptide 3 in MH7A Cells. Hu, J. et al. 2022. Front Genet. 13: 920273.
1300	35863589	PRMT1 suppresses doxorubicin-induced cardiotoxicity by inhibiting endoplasmic reticulum stress. Kim, SW. et al. 2022. Cell Signal. 110412.
1301	35864382	Polymerogenic neuroserpin causes mitochondrial alterations and activates NFκB but not the UPR in a neuronal model of neurodegeneration FENIB. D'Acunto, E. et al. 2022. Cell Mol Life Sci. 79: 437.
1302	35866843	Eplerenone reduces renal ischaemia/reperfusion injury by modulating Klotho, NF-κB and SIRT1/SIRT3/PGC-1α signalling pathways. Barati, A. et al. 2022. J Pharm Pharmacol.
1303	35868484	Dimethyl itaconate reprograms neurotoxic to neuroprotective primary astrocytes through the regulation of NLRP3 inflammasome and Nrf-2/HO-1 pathways. Khadem, MD. et al. 2022. Mol Cell Neurosci. 103758.
1304	35870982	Cytotoxic and apoptotic potential of gemini-chrysophanol nanoparticles against human colorectal cancer HCT-116 cell lines. Naqishbandi, AM. et al. 2022. BMC Pharmacol Toxicol. 23: 56.
1305	35871161	SALIS transcriptionally represses IGFBP3/Caspase-7-mediated apoptosis by associating with STAT5A to promote hepatocellular carcinoma. Liu, X. et al. 2022. Cell Death Dis. 13: 642.
1306	35872860	ZSTK474 Sensitizes Glioblastoma to Temozolomide by Blocking Homologous Recombination Repair. Jiao, W. et al. 2022. Biomed Res Int. 2022: 8568528.
1307	35874630	Long Noncoding XLOC_006390 Regulates the Proliferation and Metastasis of Human Colorectal Cancer via miR-296/ONECUT2 Axis. Ma, T. et al. 2022. J Oncol. 2022: 4897201.
1308	35877732	Porphyra tenera Protects against PM2.5-Induced Cognitive Dysfunction with the Regulation of Gut Function. Park, SK. et al. 2022. Mar Drugs. 20:
1309	35878499	Di-isononyl phthalate induces apoptosis and autophagy of mouse ovarian granulosa cells via oxidative stress. Chen, J. et al. 2022. Ecotoxicol Environ Saf. 242: 113898.
1310	35892233	Pre-Ischemic Oxytocin Treatment Alleviated Neuronal Injury via Suppressing NF-κB, MMP-9, and Apoptosis Regulator Proteins in A Mice Model of Stroke. Momenabadi, S. et al. 2022. Cell J. 24: 337-345.
1311	35896004	Increased cytochrome C threonine 50 phosphorylation in aging heart as a novel defensive signaling against hypoxia/reoxygenation induced apoptosis. Li, F. et al. 2022. Aging (Albany NY). 14: 5699-5709.
1312	35917200	Mitochondria-targeting nano therapy altering IDH2-mediated EZH2/EZH1 interaction as precise epigenetic regulation in glioblastoma. Kaundal, B. et al. 2022. Biomater Sci.
1313	35949608	Role of phloretin as a sensitizer to TRAIL-induced apoptosis in colon cancer. Kim, JL. et al. 2022. Oncol Lett. 24: 321.
1314	35955110	Hymenolepis diminuta Infection Affects Apoptosis in the Small and Large Intestine. Kapczuk, P. et al. 2022. Int J Environ Res Public Health. 19:
1315	35955813	Dimethyl Fumarate Induces Apoptosis via Inhibition of NF-κB and Enhances the Effect of Paclitaxel and Adriamycin in Human TNBC Cells. Tsurushima, K. et al. 2022. Int J Mol Sci. 23:
1316	35963563	A novel BMP2 secretagogue ameliorates glucocorticoid induced oxidative stress in osteoblasts by activating NRF2 dependent survival while promoting Wnt/β-catenin mediated osteogenesis. Rai, D. et al. 2022. Free Radic Biol Med.
1317	35982137	Novel mechanisms underlying inhibition of inflammation-induced angiogenesis by dexamethasone and gentamicin via PI3K/AKT/NF-κB/VEGF pathways in acute radiation proctitis. Li, Y. et al. 2022. Sci Rep. 12: 14116.
1318	35987413	Photoprotective activities of Lignosus rhinocerus in UV-irradiated human keratinocytes. Lim, HS. et al. 2022. J Ethnopharmacol. 115621.
1319	35993632	In vitro mitochondrial apoptosis of melanoma cells via immature Poncirus trifoliata fruit extract. Kim, SY. et al. 2022. Eur Rev Med Pharmacol Sci. 26: 5380-5392.
1320	36010941	Targeting MDM4 as a Novel Therapeutic Approach in Prostate Cancer Independent of p53 Status. Mejía-Hernández, JO. et al. 2022. Cancers (Basel). 14:
1321	36014555	Walnut Prevents Cognitive Impairment by Regulating the Synaptic and Mitochondrial Dysfunction via JNK Signaling and Apoptosis Pathway in High-Fat Diet-Induced C57BL/6 Mice. Moon, JH. et al. 2022. Molecules. 27:
1322	36030026	Pyrroloquinoline quinone (PQQ) improves pulmonary hypertension by regulating mitochondrial and metabolic functions. Shafiq, M. et al. 2022. Pulm Pharmacol Ther. 102156.
1323	36039041	Korean Red Pine (Pinus densiflora) Bark Extract Attenuates Aβ-Induced Cognitive Impairment by Regulating Cholinergic Dysfunction and Neuroinflammation. Go, MJ. et al. 2022. J Microbiol Biotechnol. 32: 1154-1167.
1324	36043787	Plakoglobin and High-Mobility Group Box 1 Mediate Intestinal Epithelial Cell Apoptosis Induced by Clostridioides difficile TcdB. Li, Y. et al. 2022. mBio. e0184922.
1325	36046646	Glycolytic Metabolic Remodeling by the Truncate of Glioma-Associated Oncogene Homolog 1 in Triple-Negative Breast Cancer Cells. Lee, SH. et al. 2022. J Cancer. 13: 3031-3043.
1326	36063341	Lupeol and Paclitaxel cooperate in hindering hypoxia induced vasculogenic mimicry via suppression of HIF-1α-EphA2-Laminin-5γ2 network in human oral cancer. Saha, D. et al. 2022. J Cell Commun Signal.
1327	36077161	Cross-Linked Hyaluronate and Corticosteroid Combination Ameliorate the Rat Experimental Tendinopathy through Anti-Senescent and -Apoptotic Effects. Ko, PY. et al. 2022. Int J Mol Sci. 23:
1328	36085167	A RUNX-targeted gene switch-off approach modulates the BIRC5/PIF1-p21 pathway and reduces glioblastoma growth in mice. Hattori, EY. et al. 2022. Commun Biol. 5: 939.
1329	36086879	Protective effect of salidroside on streptozotocin-induced diabetic nephropathy by inhibiting oxidative stress and inflammation in rats via the Akt/GSK-3β signalling pathway. Pei, D. et al. 2022. Pharm Biol. 60: 1732-1738.
1330	36089173	Coenzyme Q10 ameliorates aging-induced memory deficits via modulation of apoptosis, oxidative stress, and mitophagy in aged rats. Hosseini, L. et al. 2022. Exp Gerontol. 111950.
1331	36104956	iTRAQ-based proteomic analysis of 17β-Estradiol-induced anti-proliferation and apoptosis in mouse thymic epithelial cells by disturbing ribosomal biogenesis. Liu, Y. et al. 2022. IUBMB Life.
1332	36117462	Deficiency of ataxia-telangiectasia mutated kinase attenuates Western-type diet-induced cardiac dysfunction in female mice. Wingard, MC. et al. 2022. Physiol Rep. 10: e15434.
1333	36121569	Trehalose-Carnosine Prevents the Effects of Spinal Cord Injury Through Regulating Acute Inflammation and Zinc(II) Ion Homeostasis. Paterniti, I. et al. 2023. Cellular and molecular neurobiology. 43: 1637-1659.
1334	36139913	Eugenol Attenuates Transmissible Gastroenteritis Virus-Induced Oxidative Stress and Apoptosis Via ROS-NRF2-ARE Signaling. Wang, K. et al. 2022. Antioxidants (Basel). 11:
1335	36139915	Carob (Ceratonia siliqua) as Functional Feed Is Beneficial in Yellow Mealworm (Tenebrio molitor) Rearing: Evidence from Growth, Antioxidant Status and Cellular Responses. Antonopoulou, E. et al. 2022. Antioxidants (Basel). 11:
1336	36154440	Investigation of the Gastroprotective Effect of Betaine-Homocysteine Homeostasis on Oxidative Stress, Inflammation and Apoptosis in Ethanol-Induced Ulcer Model. Çakır Gündoğdu, A. et al. 2022. J Invest Surg. 35: 1806-1817.
1337	36170028	Novel TRKB agonists activate TRKB and downstream ERK and AKT signaling to protect Aβ-GFP SH-SY5Y cells against Aβ toxicity. Chiu, YJ. et al. 2022. Aging (Albany NY). 14: 7568-7586.

1338	36170928	Fucoidan, as an immunostimulator promotes M1 macrophage differentiation and enhances the chemotherapeutic sensitivity of capecitabine in colon cancer. Deng, Z. et al. 2022. Int J Biol Macromol. 222: 562-572.
1339	36172283	Identification of a new member of Mortaparib class of inhibitors that target mortalin and PARP1. Meidinna, HN. et al. 2022. Front Cell Dev Biol. 10: 918970.
1340	36186900	Snake venom induces an autophagic cell death via activation of the JNK pathway in colorectal cancer cells. Yu, JE. et al. 2022. J Cancer. 13: 3333-3341.
1341	36193329	ZOMEC via the p-Akt/Nrf2 Pathway Restored PTZ-Induced Oxidative Stress-Mediated Memory Dysfunction in Mouse Model. Jahan, R. et al. 2022. Biomed Res Int. 2022: 8902262.
1342	36229845	A novel cell-free intrathecal approach with PRP for the treatment of spinal cord multiple sclerosis in cats. Farid, MF. et al. 2022. Inflamm Regen. 42: 45.
1343	36232623	Chronic Exposure to Endocrine Disruptor Vinclozolin Leads to Lung Damage via Nrf2-Nf-kb Pathway Alterations. D'Amico, R. et al. 2022. International journal of molecular sciences. 23:
1344	36232707	Hepatoprotective Effects of a Natural Flavanol 3,3′-Diindolylmethane against CCl4-Induced Chronic Liver Injury in Mice and TGFβ1-Induced EMT in Mouse Hepatocytes via Activation of Nrf2 Cascade. Munakarmi, S. et al. 2022. Int J Mol Sci. 23:
1345	36248995	PCDH20 inhibits esophageal squamous cell carcinoma proliferation and migration by suppression of the mitogen-activated protein kinase 9/AKT/β-catenin pathway. Ning, Y. et al. 2022. Front Oncol. 12: 937716.
1346	36253375	Brain milieu induces early microglial maturation through the BAX-Notch axis. Zhao, F. et al. 2022. Nat Commun. 13: 6117.
1347	36271092	Single cell transcriptomic profiling of a neuron-astrocyte assembloid tauopathy model. Rickner, HD. et al. 2022. Nat Commun. 13: 6275.
1348	36283175	Novel synthesis naringenin-benzyl piperazine derivatives prevent glioblastoma invasion by inhibiting the hypoxia-induced IL6/JAK2/STAT3 axis and activating caspase-dependent apoptosis. Zaim, Ö. et al. 2022. Bioorg Chem. 129: 106209.
1349	36321740	Neuroprotective effects of a new triterpenoid from edible mushroom on oxidative stress and apoptosis through the BDNF/TrkB/ERK/CREB and Nrf2 signaling pathway in vitro and in vivo. Kou, RW. et al. 2022. Food Funct.
1350	36375514	Anti-proliferating and apoptosis-inducing activity of chemical compound FTI-6D in association with p53 in human cancer cell lines. Inoue, T. et al. 2022. Chem Biol Interact. 110257.
1351	36414027	Adverse effects of 2-Methoxyestradiol on mouse oocytes during reproductive aging. Jiang, X. et al. 2022. Chem Biol Interact. 369: 110277.
1352	36423625	The Possible Protective Effects of Ondansetron and Tropisetron on Optic Nerve Crush Injury in Rats. Shayan, M. et al. 2022. Drug Res (Stuttg).
1353	36430394	GSK343, an Inhibitor of Enhancer of Zeste Homolog 2, Reduces Glioblastoma Progression through Inflammatory Process Modulation: Focus on Canonical and Non-Canonical NF-κB/IκBα Pathways. Scuderi, S.A. et al. 2022. International journal of molecular sciences. 23:
1354	36446389	JAK2/STAT3 pathway mediates neuroprotective and pro-angiogenic treatment effects of adult human neural stem cells in middle cerebral artery occlusion stroke animal models. Ha, GH. et al. 2022. Aging (Albany NY). 14: 8944-8969.
1355	36466073	KYA1797K, a Novel Small Molecule Destabilizing β-Catenin, Is Superior to ICG-001 in Protecting against Kidney Aging. Zhu, M. et al. 2022. Kidney Dis (Basel). 8: 408-423.
1356	36496166	Novel therapeutic mechanism of action of metformin and its nanoformulation in Alzheimer's disease and role of AKT/ERK/GSK pathway. Kumar, H. et al. 2022. Eur J Pharm Sci. 106348.
1357	36496849	Inhibition of GSK3β Promotes Proliferation and Suppresses Apoptosis of Porcine Muscle Satellite Cells. Park, J. et al. 2022. Animals (Basel). 12:
1358	36498769	Protective Effects of Orexin A in a Murine Model of Cisplatin-Induced Acute Kidney Injury. Jo, J. et al. 2022. J Clin Med. 11:
1359	36499465	4-O-Methylascochlorin-Mediated BNIP-3 Expression Controls the Balance of Apoptosis and Autophagy in Cervical Carcinoma Cells. Cho, Y. et al. 2022. Int J Mol Sci. 23:
1360	36499536	Antitumor Effect of Cycloastragenol in Colon Cancer Cells via p53 Activation. Park, D. et al. 2022. Int J Mol Sci. 23:
1361	36499679	Regulation of Apoptosis and Oxidative Stress by Oral Boswellia Serrata Gum Resin Extract in a Rat Model of Endometriosis. D'Amico, R. et al. 2022. International journal of molecular sciences. 23:
1362	36512849	Knockdown of PKMYT1 is associated with autophagy inhibition and apoptosis induction and suppresses tumor progression in hepatocellular carcinoma. Wu, F. et al. 2022. Biochem Biophys Res Commun. 640: 173-182.
1363	36547896	Purification and Identification of Peptides from Oyster (Crassostrea hongkongensis) Protein Enzymatic Hydrolysates and Their Anti-Skin Photoaging Effects on UVB-Irradiated HaCaT Cells. Peng, Z. et al. 2022. Mar Drugs. 20:
1364	36552561	Phloroglucinol Attenuates DNA Damage and Apoptosis Induced by Oxidative Stress in Human Retinal Pigment Epithelium ARPE-19 Cells by Blocking the Production of Mitochondrial ROS. Park, C. et al. 2022. Antioxidants (Basel). 11:
1365	36552563	Açai Berry Attenuates Cyclophosphamide-Induced Damage in Genitourinary Axis-Modulating Nrf-2/HO-1 Pathways. Siracusa, R. et al. 2022. Antioxidants (Basel, Switzerland). 11:
1366	36573636	MiR-196a-5p facilitates progression of estrogen-dependent endometrial cancer by regulating FOXO1. Zhu, Y. et al. 2022. Histol Histopathol. 18572.
1367	36590307	Bee Venom Triggers Autophagy-Induced Apoptosis in Human Lung Cancer Cells via the mTOR Signaling Pathway. Yu, JE. et al. 2022. J Oncol. 2022: 8916464.
1368	36591304	Detrimental effects of simulated microgravity on mast cell homeostasis and function. Kim, M. et al. 2022. Front Immunol. 13: 1055531.
1369	36612214	Sodium Propionate Contributes to Tumor Cell Growth Inhibition through PPAR-γ Signaling. Filippone, A. et al. 2022. Cancers. 15:
1370	36612260	Novel Specific Pyruvate Kinase M2 Inhibitor, Compound 3h, Induces Apoptosis and Autophagy through Suppressing Akt/mTOR Signaling Pathway in LNCaP Cells. Jiang, C. et al. 2022. Cancers (Basel). 15:
1371	36613533	Anti-Amnesic Effect of Synbiotic Supplementation Containing Corni fructus and Limosilactobacillus reuteri in DSS-Induced Colitis Mice. Lee, HL. et al. 2022. Int J Mol Sci. 24:
1372	36615735	Depletion of Zinc Causes Osteoblast Apoptosis with Elevation of Leptin Secretion and Phosphorylation of JAK2/STAT3. Lee, JK. et al. 2022. Nutrients. 15:
1373	36624865	Antiacne and Anti-Inflammatory Effects of Phenolic Compounds from Quercus acutissima Carruth. Leaves. Kim, EB. et al. 2022. Evid Based Complement Alternat Med. 2022: 9078475.
1374	36627662	Laser-activated autologous adipose tissue-derived stromal vascular fraction restores spinal cord architecture and function in multiple sclerosis cat model. Farid, MF. et al. 2023. Stem Cell Res Ther. 14: 6.
1375	36640709	Protective effect of Eprosartan against ischemic acute renal injury: Acting on NF-κB, caspase 3, and Sirtuin 1. Lotfi, B. et al. 2023. Int Immunopharmacol. 115: 109690.
1376	36641751	Targeting KRAS-mutant stomach/colorectal tumors by disrupting the ERK2-p53 complex. Wang, X. et al. 2023. Cell Rep. 42: 111972.
1377	36645100	Exploring the cardioprotective effects of canagliflozin against cisplatin-induced cardiotoxicity: Role of iNOS/NF-κB, Nrf2, and Bax/cytochrome C/Bcl-2 signals. Ali, FEM. et al. 2023. J Biochem Mol Toxicol. e23309.
1378	36658493	A novel Mcl-1 inhibitor synergizes with venetoclax to induce apoptosis in cancer cells. Zhao, T. et al. 2023. Mol Med. 29: 10.
1379	36699324	Natural history of Echinococcus granulosus microcyst development in long term in vitro culture and molecular and morphological changes induced by insulin and BMP-4. Derakhshani, A. et al. 2022. Front Vet Sci. 9: 1068602.
1380	36726586	Neuroregeneration of injured peripheral nerve by fraction B of catfish epidermal secretions through the reversal of the apoptotic pathway and DNA damage. Al-Arbeed, TA. et al. 2023. Front Pharmacol. 14: 1085314.
1381	36731722	Ormeloxifene, a selective estrogen receptor modulator, protects against pulmonary hypertension. Abdulkareem, AO. et al. 2023. Eur J Pharmacol. 943: 175558.
1382	36736415	Cannabidiol alters mitochondrial bioenergetics via VDAC1 and triggers cell death in hormone-refractory prostate cancer. Mahmoud, AM. et al. 2023. Pharmacol Res. 189: 106683.
1383	36738519	Gemini curcumin inhibits 4T1 cancer cell proliferation and modulates the expression of apoptotic and metastatic genes in Balb/c mice model. Kandjani, BZ. et al. 2023. Pathol Res Pract. 243: 154344.
1384	36782269	Hepatocyte growth factor-modified hair follicle stem cells ameliorate cerebral ischemia/reperfusion injury in rats. Tang, H. et al. 2023. Stem Cell Res Ther. 14: 25.
1385	36789832	Suppression of MAPK/NF-kB and activation of Nrf2 signaling by Ajugarin-I in EAE model of multiple sclerosis. Khan, A. et al. 2023. Phytother Res.
1386	36819840	Effect of high-intensity interval training and coenzyme Q10 supplementation on cardiac apoptosis in obese male rats. Astani, K. et al. 2022. ARYA Atheroscler. 18: 1-9.
1387	36835418	Investigation of Molecular Mechanisms Involved in Sensitivity to the Anti-Cancer Activity of Costunolide in Breast Cancer Cells. Choi, YJ. et al. 2023. Int J Mol Sci. 24:
1388	36842770	Inhibition of poly (ADP-ribose) Polymerase-1 (PARP-1) improves endothelial function in pulmonary hypertension. Shafiq, M. et al. 2023. Pulm Pharmacol Ther. 102200.
1389	36847599	KPT-330 and Y219 exert a synergistic anti-tumor effect in triple-negative breast cancer through inhibiting NF-κB signaling. Wen, T. et al. 2023. FEBS Open Bio.
1390	36925030	Resveratrol alleviates doxorubicin-induced damage in mice ovary. Herrero, Y. et al. 2023. Chem Biol Interact. 376: 110431.
1391	36964998	Inhibition of hippocampal cyclin-dependent kinase 5 activity ameliorates learning and memory dysfunction in a mouse model of bronchopulmonary dysplasia. Tao, FF. et al. 2023. CNS Neurosci Ther. 29: 2339-2354.
1392	36988254	Protective effects of cordycepin pretreatment against liver ischemia/reperfusion injury in mice. Liu, Y. et al. 2023. Immun Inflamm Dis. 11: e792.
1393	36996895	Umbelliferone Ameliorates Hepatic Steatosis and Lipid-Induced ER Stress in High-Fat Diet-Induced Obese Mice. Park, NW. et al. 2023. Yonsei Med J. 64: 243-250.
1394	37008023	Recombinant irisin prevents cell death and mineralization defects induced by random positioning machine exposure in primary cultures of human osteoblasts: A promising strategy for the osteoporosis treatment. Cariati, I. et al. 2023. Front Physiol. 14: 1107933.
1395	37009001	Comparison of the anti-diabetic and nephroprotective activities of vitamin E, metformin, and Nigella sativa oil on kidney in experimental diabetic rats. Ayaz, H. et al. 2023. Iran J Basic Med Sci. 26: 395-399.
1396	37069612	Statins enhances antitumor effect of oxaliplatin in KRAS-mutated colorectal cancer cells and inhibits oxaliplatin-induced neuropathy. Tsubaki, M. et al. 2023. Cancer Cell Int. 23: 73.
1397	37088118	Polyphyllin D-loaded solid lipid nanoparticles for breast cancer: Synthesis, characterization, in vitro, and in vivo studies. Emami, A. et al. 2023. Int J Pharm. 639: 122976.
1398	37108231	Anti-Cancer SERCA Inhibitors Targeting Sorafenib-Resistant Human Papillary Thyroid Carcinoma. Chang, HS. et al. 2023. Int J Mol Sci. 24:
1399	37111248	Antimetastatic Activity of Apoptolidin A by Upregulation of N-Myc Downstream-Regulated Gene 1 Expression in Human Colorectal Cancer Cells. Kyaw, KZ. et al. 2023. Pharmaceuticals (Basel). 16:
1400	37112622	Tebuconazole Induces ER-Stress-Mediated Cell Death in Bovine Mammary Epithelial Cell Lines. Lee, WY. et al. 2023. Toxics. 11:
1401	37130475	Exposure to polystyrene particles causes anxiety-, depression-like behavior and abnormal social behavior in mice. Shin, HS. et al. 2023. J Hazard Mater. 454: 131465.
1402	37181451	Synthetic miR-21 decoy circularized by tRNA splicing mechanism inhibited tumorigenesis in glioblastoma in vitro and in vivo models. Bayat, H. et al. 2023. Mol Ther Nucleic Acids. 32: 432-444.
1403	37185276	Sirtuin 6 is a key contributor to gender differences in acute kidney injury. Miao, J. et al. 2023. Cell Death Discov. 9: 134.
1404	37228640	Magnetic Fields Reduce Apoptosis by Suppressing Phase Separation of Tau-441. Lin, WJ. et al. 2023. Research (Wash D C). 6: 0146.
1405	37233859	β-caryophyllene oxide induces apoptosis and inhibits proliferation of A549 lung cancer cells. Shabana, SM. et al. 2023. Med Oncol. 40: 189.
1406	37269951	The role of microglial LRRK2 kinase in manganese-induced inflammatory neurotoxicity via NLRP3 inflammasome and RAB10-mediated autophagy dysfunction. Pajarillo, E. et al. 2023. J Biol Chem. 299: 104879.
1407	37275757	Chronic stress-induced apoptosis is mitigated by young mitochondria transplantation in the prefrontal cortex of aged rats. Javani, G. et al. 2023. Iran J Basic Med Sci. 26: 725-730.
1408	37286116	Porcine cardiac blood - Salvia miltiorrhiza root alleviates cerebral ischemia reperfusion injury by inhibiting oxidative stress induced apoptosis through PI3K/AKT/Bcl-2/Bax signaling pathway. Zhou, S. et al. 2023. J Ethnopharmacol. 316: 116698.
1409	37298244	Repeated Rounds of Gonadotropin Stimulation Induce Imbalance in the Antioxidant Machinery and Activation of Pro-Survival Proteins in Mouse Oviducts. Di Nisio, V. et al. 2023. Int J Mol Sci. 24:
1410	37344027	Effects of heat stress exposure on porcine muscle satellite cells. Park, J. et al. 2023. J Therm Biol. 114: 103569.
1411	37373070	Antidiabetic and Immunoregulatory Activities of Extract of Phyllanthus emblica L. in NOD with Spontaneous and Cyclophosphamide-Accelerated Diabetic Mice. Lin, CH. et al. 2023. Int J Mol Sci. 24:
1412	37391829	Neuroprotective effects of GSK-343 in an in vivo model of MPTP-induced nigrostriatal degeneration. Mannino, D. et al. 2023. Journal of neuroinflammation. 20: 155.
1413	37511015	Synergistic Effects of Vitis vinifera L. and Centella asiatica against CCl4-Induced Liver Injury in Mice. Munakarmi, S. et al. 2023. Int J Mol Sci. 24:
1414	37511082	Protective Effect of Water-Soluble Acacetin Prodrug on APAP-Induced Acute Liver Injury Is Associated with Upregulation of PPARγ and Alleviation of ER Stress. Miao, J. et al. 2023. Int J Mol Sci. 24:
1415	37524903	Long non-coding RNA SOX2-OT enhances cancer biological traits via sponging to tumor suppressor miR-122-3p and miR-194-5p in non-small cell lung carcinoma. Dodangeh, F. et al. 2023. Sci Rep. 13: 12371.
1416	37570345	Breeding Substrate Containing Distillation Residues of Mediterranean Medicinal Aromatic Plants Modulates the Effects of Tenebrio molitor as Fishmeal Substitute on Blood Signal Transduction and WBC Activation of Gilthead Seabream (Sparus aurata). Antonopoulou, E. et al. 2023. Animals (Basel). 13:
1417	37586618	Administration of intestinal mesenchymal stromal cells reduces colitis-associated cancer in C57BL/6J mice modulating the immune response and gut dysbiosis. Hidalgo-García, L. et al. 2023. Pharmacol Res. 195: 106891.
1418	37649095	Hepatocyte-specific Sox9 knockout ameliorates acute liver injury by suppressing SHP signaling and improving mitochondrial function. Qin, D. et al. 2023. Cell Biosci. 13: 159.
1419	37686282	Androgens Modulate Bcl-2 Agonist of Cell Death (BAD) Expression and Function in Breast Cancer Cells. Morelli, C. et al. 2023. Int J Mol Sci. 24:

Supplementary Information 2

HCT 116 WT

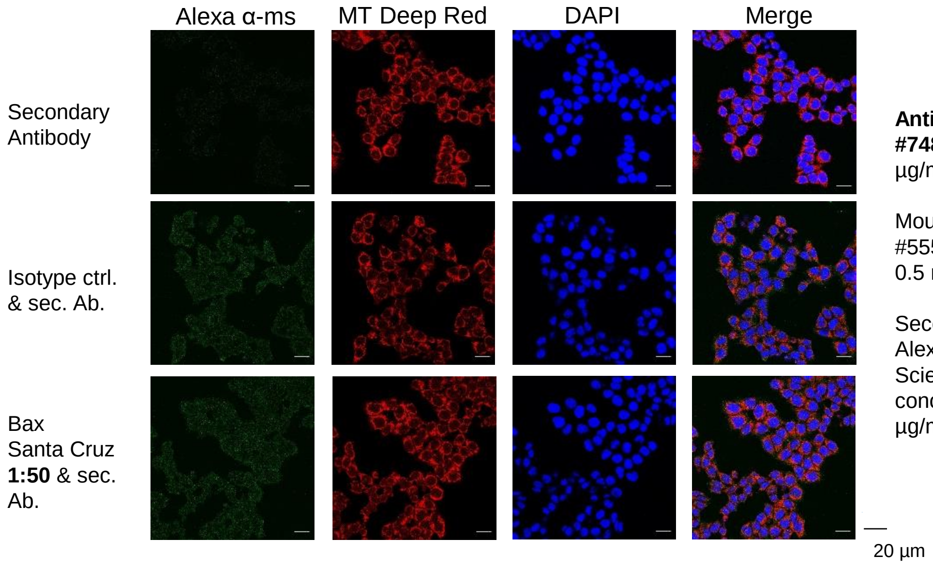

Anti-Bax (B-9) Santa Cruz #7480; α-ms; stock conc.: 200 µg/ml; Final conc.: 4 µg/ml

Mouse Isotype control (BD #555740**)** IgG2b κ: stock conc.: 0.5 mg/ml; Final conc.: 4 µg/ml

Secondary antibody: Anti-mouse Alexa Fluor 488 (Thermo Fisher Scientific, #A-11029): stock conc.: 2 mg/ml; Final conc.: 4 µg/ml

HCT 116 (Bax/Bak)$^{-/-}$

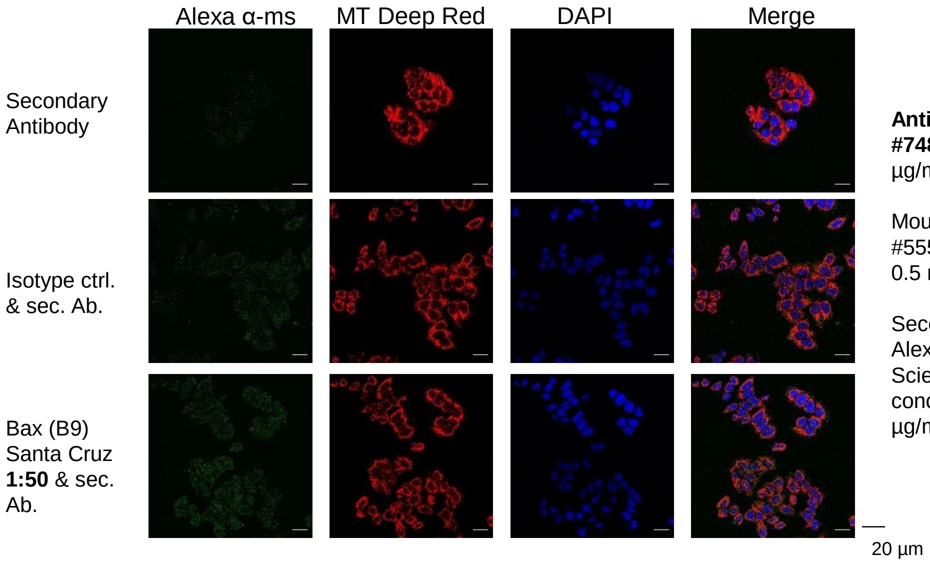

Anti-Bax (B-9) Santa Cruz #7480; α-ms; stock conc.: 200 µg/ml; Final conc.: 4 µg/ml

Mouse Isotype control (BD #555740**)** IgG2b κ: stock conc.: 0.5 mg/ml; Final conc.: 4 µg/ml

Secondary antibody: Anti-mouse Alexa Fluor 488 (Thermo Fisher Scientific, #A-11029): stock conc.: 2 mg/ml; Final conc.: 4 µg/ml

HCT 116 WT

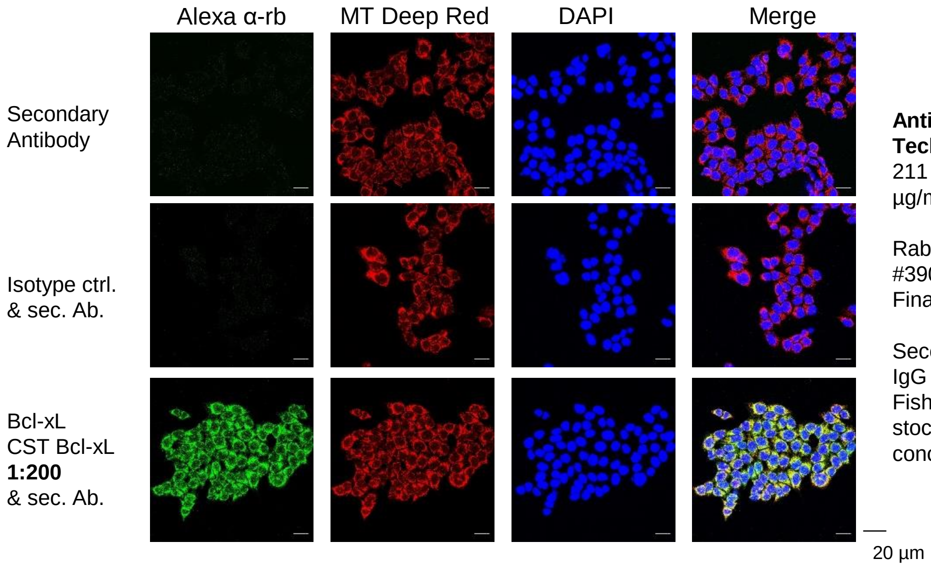

Anti-Bcl-xL Cell Signaling Technology; α-rb; stock conc.: 211 µg/ml; Finale conc.: 1.1 µg/ml

Rabbit Isotype control (CST #3900): stock conc.: 2.5 mg/ml; Final conc.: 1.1 µg/ml

Secondary antibody: Anti-rabbit IgG Alexa Fluor 488 (Thermo Fisher Scientific, #A-11008): stock conc.: 2 mg/ml; Final conc.: 4 µg/ml

Supplementary Information 3

Why Bax detection in >1,400 publications might be flawed

Running title: Reliability of Bax detection

Kristin Entrop[1], Senait Wieske[1], Markus Rehm[1, 2*]

Materials and Methods

Antibodies

The following antibodies were used for immunoblotting and immunofluorescence analysis. A mouse monoclonal Bax antibody (B-9, sc-7480, Santa Cruz Biotechnology; WB: 1:500; IF: 1:50), a rabbit polyclonal Bax antibody (#2772, Cell Signaling Technology; WB: 1:1,000), a mouse monoclonal GAPDH antibody (#97166, Cell Signaling Technology; WB: 1:2,000), and a mouse isotype control antibody (#555740, BD Pharmigen™; IF: 1:125). Secondary antibodies used include a goat polyclonal anti-rabbit antibody (#111-035-144, Dianova; WB: 1:10,000), a goat polyclonal anti-mouse antibody (115-035-146, Dianova; WB: 1:10,000), a goat polyclonal anti-mouse Alexa Fluor™ 488 antibody (#A-11029, Thermo Fisher Scientific; IF: 1:500).

Cell lines, cell culture and transfection

HCT116 were from ATCC. HCT116 (Bax/Bak)$^{-/-}$ cells were a gift from R. Youle (NIH, Bethesda, MD) (1). Cell line authenticity was verified by STR profiling. The Bax deficient HCT116 cell line from which the (Bax/Bak)-deficient cells were generated was

previously reported in (2). In there, *BAX* was disrupted by targeted deletion of Exons 2-4 via homologous recombination. MEF and MEF (Bax/Bak)$^{-/-}$ cells were a gift from S. Oakes (University of California, San Francisco, CA) and were first described in (3). (Bax/Bak)-deficient MEFs were generated by immortalizing MEFs via transfection of a plasmid containing SV40 genomic DNA and timed matings of Bax$^{+/-}$; Bak$^{+/-}$ or Bax$^{+/-}$/Bak$^{-/-}$ animals. Cells were regularly mycoplasm tested.

Cells were grown in Roswell Park Memorial Institute medium (RPMI, Life Technologies Corporation, Carlsbad, CA, USA) with 10% FCS (Fetal Calf Serum, Sigma-Aldrich Life Science, St. Louis, MO, USA). All cultures were regularly tested for mycoplasm infection. For transfection of siRNAs, Lipofectamine™ RNAiMAX Reagent (Thermo Fisher Scientific) was used according to the manufacturer's protocol. siRNAs targeting Bax expression were from Ambion, Life Technologies Ltd (Carlsbad, CA): siRNA ID # s1890 (sense GAACUGAUCAGAACCAUCAtt, antisense UGAUGGUUCUGAUCAGUUCcg), siRNA ID # s1889 (sense ACAUGUUUUCUGACGGCAAtt, antisense UUGCCGUCAGAAAACAUGUca).

Western Blotting

Cells were resuspended in ice-cold PBS before they were centrifuged at 300 *g* for five minutes at 4°C. The supernatant was aspirated, and the cells were suspended in lysis buffer, with addition of 1x c0mplete (Roche Diagnostics International AG, Rotkreuz Switzerland), and incubated for 15 min on ice. Subsequently, cells were centrifuged at 16,100 *g* for five minutes at 4°C to remove cellular debris, and protein concentrations were quantified by Bradford assay. Equal amounts of proteins were supplemented with the corresponding amounts of 5x Laemmli sample buffer (10% SDS, 312.5 mM Tris pH 6.8, 25% β-mercaptoethanol, 25% glycerin, 0.05% bromphenol blue, all chemicals were purchased from Carl Roth, Karlsruhe, Germany), and heated to 95°C for five min

with subsequent centrifugation at 16,100 *g* for one minute. Proteins were separated on 4-12% Bolt™ Bis-Tris gels (Thermo Fisher Scientific Corporation, Waltham, MA, USA) and transferred to nitrocellulose membranes (Thermo Fisher Scientific Corporation, Waltham, MA, USA) using an iBlot® 2 gel transfer device (Life Technologies Corporation, Carlsbad, CA, USA). After 1 h blocking at room temperature with blocking reagent (Roche Diagnostics, Mannheim, Germany) diluted in TBST (1%) the membranes were incubated with primary antibodies (diluted in TBST with 0.5% blocking reagent) overnight at 4°C. Following washing with TBST, membranes were incubated with the respective horseradish peroxidase (HRP)-coupled secondary antibody (diluted in TBST with 0.5% blocking reagent) for 1 h at room temperature. Following three further washing steps, proteins were detected by incubating the membrane with an HRP Dura substrate (SuperSignal™ West Dura Luminol/Enhancer/SuperSignal™ West Dura Stable Peroxide, Thermo Fisher Scientific Corporation, Waltham, MA, USA) and signals were acquired with an ECL imager (Amersham™ Imager 600, GE Healthcare Life Sciences, Chicago, IL, USA). Luminescence was detected at a depth of 12-bit in the linear detection range. For presentation, images were converted to 8 bit and contrast-adjusted with ImageJ (National Institute of Health, USA, http://rsb.info.nih.gov/ij).

Immunofluorescence staining

Cells were grown on coverslips in RPMI medium (Life Technologies Corporation, Carlsbad, CA, USA) including 10% FCS (Sigma-Aldrich Life Science, St. Louis, MO, USA). Cells were washed with PBS two times and fixed using 4% paraformaldehyde (PFA) (Santa Cruz Biotechnology, Dallas, TX, USA) in PBS for 20 min at room temperature. After washing, cells were permeabilized with 0.1% Triton X-100 (Carl Roth GmbH + Ko. KG, Karlsruhe, Germany) for a maximum of five minutes at room

temperature. Wells were washed with PBS and then blocked with 5% FCS in PBS (FCS/PBS) for 30 min at room temperature before incubation with primary antibody for 1.5 h at room temperature. Cells were washed three times with PBS and subsequently incubated with an anti-mouse Alexa Fluor 488 antibody for 45 min at room temperature. Cells were washed three times with PBS and the wells were filled with demineralized water. Coverslip specimens were mounted on microscope slides with Fluoromount-G (Southern Biotechnology Associates, Inc., Birmingham, USA) and images were acquired on a LSM T-PMT 710 fluorescence microscope (Carl Zeiss, Oberkochen, Germany). The fluorophore was excited with a 488 nm diode laser using a 525/50 nm emission filter.

Literature analysis

The list of references provided for the use of Bax antibody B-9 (Santa Cruz Biotechnology) was retrieved from https://www.scbt.com/de/p/bax-antibody-b-9#citations (last date accessed 13.05.2024) and imported into Microsoft Excel. 100 publications were randomly selected and analysed for the methodology in which the antibody was used and whether controls were included that indicated the specificity of the antibody.

References

1. Wang C, Youle RJ. Predominant requirement of Bax for apoptosis in HCT116 cells is determined by Mcl-1's inhibitory effect on Bak. Oncogene. 2012;31(26):3177-89; 10.1038/onc.2011.497.
2. Zhang L, Yu J, Park BH, Kinzler KW, Vogelstein B. Role of BAX in the apoptotic response to anticancer agents. Science. 2000;290(5493):989-92; 10.1126/science.290.5493.989.
3. Wei MC, Zong WX, Cheng EH, Lindsten T, Panoutsakopoulou V, Ross AJ, et al. Proapoptotic BAX and BAK: a requisite gateway to mitochondrial dysfunction and death. Science. 2001;292(5517):727-30.

Supplementary Information 4

No.	PMID	Title	Year	Methods used	Cell Line	Dilution	Negative Control	Notes	
480	# 27123245	piR-651 and its function in 95-D lung cancer cells	2016	WB	95-D lung cancer cells	?	No	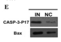 The expression of caspase-p17 and bax was upregulated and bcl-2 was downregulated in the piR-651 inhibitor. IN, piR-651 inhibitor; **NC, negative control**; FITC, fluorescein isothiocyanate; bcl-2, B-cell lymphoma-2.	2
1253	# 33219891	Squalene deters drivers of RCC disease progression beyond *VHL* status	2021	WB	Kidney Tissue Samples	1:500	No	From supplementary data (S1). 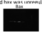	3
1305	# 32167384	Calprotectin (S100A8/S100A9)-induced cytotoxicity and apoptosis in human gastric cancer AGS cells: Alteration in expression levels of Bax, Bcl-2, and ERK2	2020	WB	S100A8/S100A9-treated AGS cells	1:200	No	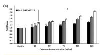	4
387	# 29669932	Nardilysin controls intestinal tumorigenesis through HDAC1/p53-dependent transcriptional regulation	2018	WB	HCT116	?	No	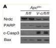	5
777	# 20511721	Hypertonicity-induced mitochondrial membrane permeability in renal medullary interstitial cells: protective role of osmolytes	2010	IF, WB	MIC cells	IF: 1:200; WB: ?	Supposedly yes (very poor Western Blot with claimed Bax siRNA. No methods section on transfection, no information on source or sequence of siRNA, no mol. weight marker.	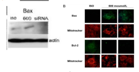	6
895	# 16374552	Cyclin D1 overexpression induces epidermal growth factor-independent resistance to apoptosis linked to BCL-2 in human A431 carcinoma	2006	SDS-PAGE	A431	?	No	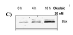	7
1093	# 32638843	Changrui enema inhibits inflammation-induced angiogenesis in acute radiation proctitis by regulating NF-κB and VEGF	2020	WB	Rectal tissues & spleen tissues	1:1000	No	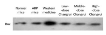	8
514	# 27710854	Albendazole as a promising molecule for tumor control	2016	WB	Ehrlich tumor cells	?	No	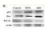	9
742	# 19892564	Tumor necrosis factor alpha induced by Trypanosoma cruzi infection mediates inflammation and cell death in the liver of infected mice	2009	WB	Hepatocytes	0.25	No	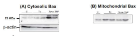	10
254	# 34128234	Evaluating the effect of secretome of human amniotic mesenchymal stromal cells on apoptosis induction and epithelial-mesenchymal transition inhibition in LNCaP	2021	WB	hAMSC & LNCaP	1:300	No	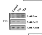	11
60	# 35083997	Tilapia skin peptides restore cyclophosphamide-induced premature ovarian failure *via* inhibiting oxidative stress and apoptosis in mice	2022	WB	ovarian (granulosa) mice cells	?	No	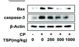	12
555	# 26360675	Impact of L-carnitine and Selenium Treatment on Testicular Apoptosis in Rats Exposed to 2.45 GHz Microwave Energy	2015	IHC	12 week old male Wistar albino rats (different treatments)	1:50	No	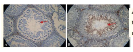 **A**: Immunolabelling relating to sham group [×40]. Normal appearance of seminiferous tubules and Leydig cells of the testes tissue after immunolabelling. **B**: Immunolabelling of Bax activity (+++) relating to 2.45 GHz electromagnetic radiation (EMR) groups [×40]. Indicator of apoptosis Bax activity increased after 2.45 GHz EMR exposure. **C**:	13
143	# 32740515	Anti-TNF-α Therapy Exerts Intestinal Anti-inflammatory and Anti-apoptotic Effects After Massive Bowel Resection in a Rat	2021	WB	Seminiferous tubules and Leydig cells from rat tissue samples	1:1000	No		14
494	# 27133421	Gamma-tocotrienol acts as a BH3 mimetic to induce apoptosis in neuroblastoma SH-SY5Y cells	2016	WB	SH-SY5Y	?	No	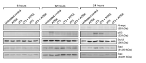	15
770	# 20663862	Overexpression of CD133 promotes drug resistance in C6 glioma cells	2010	WB	C6-GFPCD133	1:200	No	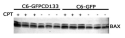	16
418	# 28123477	Dexmedetomidine alleviates cerebral ischemia-induced short-term memory impairment by inhibiting the expression of apoptosis-related molecules in the hippocampus of gerbils	2017	WB	Gerbil hippocampal tissue	1:1000	No	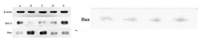	17
1018	# 12237847	TGF-beta induces cell death in the oligodendroglial cell line OLI-neu	2002	WB	OLI-neu	1:100	No	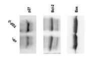	18
574	# 26668648	In vitro analysis of the role of the mitochondrial apoptosis pathway in CSBE therapy against human gastric cancer	2015	WB	SGC-7901	1:200	No	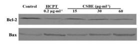	19
454	# 28260081	Minocycline attenuates sevoflurane-induced cell injury via activation of Nrf2	2017	WB	H4	1:1000	No	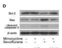	20
56	# 35227836	Human umbilical cord mesenchymal stem cell-derived extracellular vesicles loaded with miR-223 ameliorate myocardial infarction through P53/S100A9 axis	2022	IHC	myocardial tissue rat	1:200	No	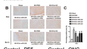	21
732	# 21223552	A human ribonuclease induces apoptosis associated with p21WAF1/CIP1 induction and JNK inactivation	2011	WB	NCI/ADR-RES	1:1000	No	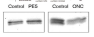	22
476	# 27012679	Honokiol inhibits sphere formation and xenograft growth of oral cancer side population cells accompanied with JAK/STAT signaling pathway suppression and apoptosis induction	2016	WB	CSCs-like SP cells from OSCC cell lines SAS and OECM-1	?	No	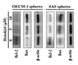	23
382	# 30103475	4-Acetyl-Antroquinonol B Suppresses SOD2-Enhanced Cancer Stem Cell-Like Phenotypes and Chemoresistance of Colorectal Cancer Cells by Inducing hsa-miR-324 re-Expression	2018	WB	CRC (DLD-1 and HCT116)	1:1000	No	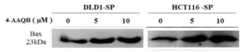	24
935	# 16018998	Low temperature protects mammalian cells from apoptosis initiated by various stimuli in vitro	2005	WB	Mouse BALB/3T3, MEF, HeLa, U-2 OS	?	No	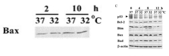	25
553	# 26300396	Fibrinogen-derived fibrinostatin inhibits tumor growth through anti-angiogenesis	2015	WB	HUVEC	?	No	Control is with PBS 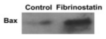	26
758	# 20164374	Hyperglycemia induces apoptosis in rat liver through the increase of hydroxyl radical: new insights into the insulin effect	2010	WB	liver tissues from Wistar rats	1:600	No	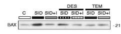	27
976	# 15547822	Expression of apoptosis-related genes after fetal tracheal occlusion in rabbits	2004	WB	whole lung homogenates; New Zealand White rabbits	?	No	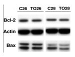	28
651	# 24195080	Phytoestrogen α-Zearalanol attenuates homocysteine-induced apoptosis in human umbilical vein endothelial cells	2013	WB, IHC	HUVECs	WB:1:200; IHC: 1:300	No	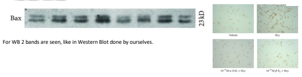 For WB 2 bands are seen, like in Western Blot done by ourselves.	29
477	# 27565756	Increased DNA double-strand break was associated with downregulation of repair and upregulation of apoptotic factors in rat hippocampus after alcohol exposure	2016	WB	hippocampal tissue of Sprague-Dawley rats	1:250	No	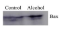	30
1273	# 32278271	Chemopreventive role of arabinoxylan rice bran, MGN-3/Biobran, on liver carcinogenesis in rats	2020	Flow cytometry	liver specimen from Wistar rats	?	No	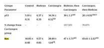	31
729	# 21900958	Essential requirement of cytochrome c release for caspase activation by procaspase-activating compound defined by cellular models	2011	WB	MEF Bax/Bak DKO & WT	?	Yes	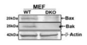	32
45	# 35785730	GLP-1 mediates the neuroprotective action of crocin against cigarette smoking-induced cognitive disorders via suppressing HMGB1-RAGE/TLR4-NF-κB pathway	2022	IHC	Hippocampal sections (male Wistar rats (8 weeks)	1:100	No	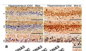	33
849	# 17885668	Bax-inhibiting peptide protects cells from polyglutamine toxicity caused by Ku70 acetylation	2007	Immunoprecipitation	HEK293T	1:100	No	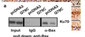	34
145	# 33920212	Nicotinamide Ameliorates Amyloid Beta-Induced Oxidative Stress-Mediated Neuroinflammation and Neurodegeneration in Adult Mouse Brain	2021	WB	mouse brain	1:1000	No	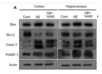	35
123	# 35955110	*Hymenolepis diminuta* Infection Affects Apoptosis in the Small and Large Intestine	2022	WB, IHC	Small (A)/ large (B) intestine Wistar rats	WB: 1:400, IHC: 1:100	No	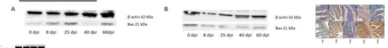	36
472	# 27499118	Inhibitory effect of metformin combined with gemcitabine on pancreatic cancer cells in vitro and in vivo	2016	WB	CFPAC-1 cells injected into nude mice	1:1000	No	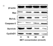	37
175	# 33566162	Cell death induction (extrinsic versus intrinsic apoptotic pathway) by intestinal ischemia-reperfusion injury in rats is time-depended	2021	WB	small intestine Sprague-Dawley male rats	1:1000	No	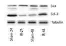	38
570	# 26475712	Mesenchymal stem cells suppress neuronal apoptosis and decrease IL-10 release via the TLR2/NFκB pathway in rats with hypoxic-ischemic brain damage	2015	WB	(SPF)-grade Wistar rats (6 weeks old)	1:500	No	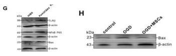	39
752	# 20537511	Eugenol restricts DMBA croton oil induced skin carcinogenesis in mice: downregulation of c-Myc and H-ras, and activation of p53 dependent apoptotic pathway	2010	WB	skin tissue from Swiss albino mice	?	No	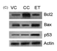	40
905	# 16572399	c-Myc-dependent etoposide-induced apoptosis involves activation of Bax and caspases, and PKCdelta signaling	2006	WB	Rat1	?	No	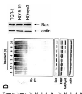	41
669	# 22912669	Withaferin A inhibits the proteasome activity in mesothelioma in vitro and in vivo	2012	WB	MPM patient derived H2373	?	No		42
977	# 14712225	The tumor suppressor p16(INK4a) gene is a regulator of apoptosis induced by ultraviolet light and cisplatin	2004	WB	U2OS	?	No	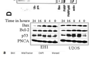	43

1192	# 23907458	Wip1 suppresses apoptotic cell death through direct dephosphorylation of BAX in response to γ-radiation	2013	IF	HeLa, LNCaP	?	No	44
1078	# 10854211	Increased expression and activation of stress-activated protein kinases/c-Jun NH(2)-terminal protein kinases in atherosclerotic lesions coincide with p53	2000	WB	aorta serum from New Zealand White rabbits	?	No	45
997	# 12531875	Defective TNF-alpha-mediated hepatocellular apoptosis and liver damage in acidic sphingomyelinase knockout mice	2003	WB	mouse hepatocytes	1:200	No	46
1164	# 15972297	Oxidative Bax dimerization promotes its translocation to mitochondria independently of apoptosis	2005	IF	U937	?	No	47
1244	# 31773909	Liraglutide Lowers Body Weight Set Point in DIO Rats and its Relationship with Hypothalamic Microglia Activation	2020	IF	brain samples/microglia from Sprague-Dawley rats	1:50	No	48
1201	# 35738618	Protective Effects of Gamma-mangostin on Hydrogen Peroxideinduced Cytotoxicity in Human Retinal Pigment Epithelial Cells	2022	WB	ARPE-19	?	No	49
781	# 18767070	Apple polyphenol phloretin potentiates the anticancer actions of paclitaxel through induction of apoptosis in human hep G2 cells	2008	WB	Hep-G2	?	No	50
97	# 35856441	Administration of 4-hexylresorcinol increases p53-mediated transcriptional activity in oral cancer cells with the p53 mutation	2022	WB; IHC	YD-15, YD-9	WB: 1:1000; Xeno	No	51
833	# 18798056	Susceptibility for NSAIDs-induced apoptosis correlates to p53 gene status in gastric cancer cells	2009	WB	AGS; MKN-28	1:100	No	52
1308	# 25828396	Activation of PPAR/ protects cardiac myocytes from oxidative stress-induced apoptosis by suppressing generation of reactive oxygen/nitrogen species and expression of matrix metalloproteinases	2015	WB	cardiac myocytes from Wistar rats	?	No	53
473	# 26898445	RO-3306 prevents postovulatory aging-mediated spontaneous exit from M-II arrest in rat eggs cultured in vitro	2016	IF	ovary & oviduct (eggs) from rats	1:500	No	54
199	# 34666484	Ganoderterpene A, a New Triterpenoid from *Ganoderma lucidum* , Attenuates LPS-Induced Inflammation and Apoptosis via Suppressing MAPK and TLR-4/NF-κB Pathways in BV-2 Cells	2021	WB	BV-2 microglia cells	?	No	55
1101	# 32703236	Yin Yang 1 is critical for mid-hindbrain neuroepithelium development and involved in cerebellar agenesis	2020	IF	MHB neuroepithelium	?	No	56
378	# 29843746	miR-519a enhances chemosensitivity and promotes autophagy in glioblastoma by targeting STAT3/Bcl2 signaling pathway	2018	WB	U87-MG/TMZ and U87-MG cells	?	No	57
395	# 29360968	Porcine parvovirus infection impairs progesterone production in luteal cells through mitogen-activated protein kinases, p53, and mitochondria-mediated apoptosis	2018	WB	primary porcine luteal cells from CL of gilts	?	No	58
286	# 30834631	Pongapin and Karanjin, furanoflavanoids of *Pongamia pinnata* , induce G2/M arrest and apoptosis in cervical cancer cells by differential reactive oxygen species modulation, DNA damage, and nuclear factor kappa-light-chain-enhancer of activated B cell signaling	2019	WB	HeLa	?	No	59
572	# 26021820	ermato-protective properties of ergothioneine through induction of Nrf2/ARE-mediated antioxidant genes in UVA-irradiated Human keratinocytes	2015	WB	HaCaT	?	No	60
395	# 29360968	Porcine parvovirus infection impairs progesterone production in luteal cells through mitogen-activated protein kinases, p53, and mitochondria-mediated apoptosis	2018	WB	primary porcine luteal cells from the CL of gilts	1:1000	No	61
1304	# 15869997	Influence of p53 and bcl-2 on chemosensitivity in benign and malignant prostatic cell lines	2005	WB	1532T, 1535T, 1542T, 1542N, BPH-1 and LNCaP prostate cells; bladder carcinoma cell line RT4 as a positive control	1:3000	No	62
940	# 15941375	Progesterone differentially regulates pro- and anti-apoptotic gene expression in cerebral cortex following traumatic brain injury in rats	2005	WB	cerebral cortex tissue from Sprague-Dawley rats	?	No	63
590	# 24289330	FoxO3a modulation and promotion of apoptosis by interferon-α2b in rat preneoplastic liver	2013	WB	liver samples from Wistar rats	?	No	64
881	# 16242231	Effect of subchronic exposure to acrylamide induced on the expression of bcl-2, bax and caspase-3 in the rat nervous system	2006	WB	cerebral cortex of Wistar rats	1:1000	No	65
702	# 22750966	Extracellular release of annexin II from pancreatic cancer cells and resistance to anticancer drug-induced apoptosis by supplementation of recombinant annexin II	2012	WB	MiaPaCa-2, HeLa	1:1000	No	66
441	# 28762326	Long Noncoding RNA Urothelial Carcinoma-Associated 1 Promotes the Proliferation and Metastasis of Human Lung Tumor Cells by Regulating MicroRNA-144	2018	WB	A549	?	No	67
1299	# 16888648	Altered gene expression in cells from patients with lysosomal storage disorders suggests impairment of the ubiquitin pathway	2006	WB	skin fibroblasts from patients affected with LSD	?	No	68
735	# 21104071	Inorganic phosphate induces mammalian growth plate chondrocyte apoptosis in a mitochondrial pathway involving nitric oxide and JNK MAP kinase	2011	WB	chondrocytes from Sprague-Dawley rats	?	No	69
702	# 22750966	Extracellular Release of Annexin II From Pancreatic Cancer Cells and Resistance to Anticancer Drug–Induced Apoptosis by Supplementation of Recombinant Annexin II	2012	WB	MiaPaCa-2; HeLa	1:1000	No	70
622	# 24427275	Gambogenic acid kills lung cancer cells through aberrant autophagy	2014	WB	A549 with siRNA against Beclin 1	?	No	71
923	# 15827328	Resveratrol-induced apoptotic death in human U251 glioma cells	2005	WB	U251/U87	?	No	72
1288	# 17091766	Apoptotic Markers p53, Bcl-2 and Bax in Primary Lung Cancer	2006	IHC	NSCLC, SCLC, bronchial stumps tissue samples	?	No	73
654	# 24330704	Eugenol triggers apoptosis in breast cancer cells through E2F1/survivin down-regulation	2013	WB	MDA-MB-231	?	No	74
231	# 34729850	Gigantol inhibits proliferation and enhanced oxidative stress-mediated apoptosis through modulating of Wnt/β-catenin signaling pathway in HeLa cells	2021	WB	HeLa	1:1000	No	75
1187	# 16430474	Cell cycle regulators and apoptosis-associated proteins in relation to proliferative activity and degree of apoptosis in HNPCC versus sporadic endometrial carcinoma	2006	IHC	endometrial cancer tissue blocks	1:200	No	76
382	# 30103475	4-Acetyl-Antroquinonol B Suppresses SOD2-Enhanced Cancer Stem Cell-Like Phenotypes and Chemoresistance of Colorectal Cancer Cells by Inducing hsa-miR-324 re-Expression	2018	WB	CRC	1:1000	No	77
10	# 35166983	Neuregulin-1 regulates the conversion of M1/M2 microglia phenotype via ErbB4-dependent inhibition of the NF-κB pathway	2022	WB; IF	SH-SY5Y	WB: 1:1000; IF: 1:100	No	78
1032	# 11694525	Restoration of Transforming Growth Factor-β Signaling Enhances Radiosensitivity by Altering the Bcl-2/Bax Ratio in the p53 Mutant Pancreatic Cancer Cell Line MIA PaCa-2	2002	WB	MIA PaCa-2	?	No	79
327	# 31068208	Anti-EMT properties of CoQ0 attributed to PI3K/AKT/NFKB/MMP-9 signaling pathway through ROS-mediated apoptosis	2019	WB	MDA-MB-231	?	No	80
1188	# 15172883	Minocycline inhibits apoptosis and inflammation in a rat model of ischemic renal injury	2004	WB	kidney cells from Sprague-Dawley rats	?	No	81
1191	# 31768967	PARP inhibitor Olaparib Enhances the Apoptotic Potentiality of Curcumin by Increasing the DNA Damage in Oral Cancer Cells through Inhibition of BER Cascade	2019	WB	H-357; xenograft mice tissue lysate	1:1000	No	82
1270	# 31683688	Kinesin Eg5 Targeting Inhibitors as a New Strategy for Gastric Adenocarcinoma Treatment	2019	WB	AGS	1:200	No	83
675	# 21543203	Flavokawain B inhibits growth of human squamous carcinoma cells: Involvement of apoptosis and cell cycle dysregulation in vitro and in vivo	2012	WB	KB cells	?	No	84
866	# 17123509	Skeletal unloading induces osteoblast apoptosis and targets α5β1-PI3K-Bcl-2 signaling in rat bone	2007	WB	Metaphyseal area in left femurs of Wistar rats	1:500	No	85
1128	# 34490824	Thymoquinone and Costunolide Induce Apoptosis of Both Proliferative and Doxorubicin-Induced-Senescent Colon and Breast Cancer Cells	2021	Flow cytometry	HCT116; MCF7	?	No	86
295	# 30790227	Clinical significance of metabolism-related biomarkers in non-Hodgkin lymphoma – MCT1 as potential target in diffuse large B cell lymphoma	2019	WB	OZ; DOHH-2	1:500	No	87
945	# 15466854	Pea3 transcription factor cooperates with USF-1 in regulation of the murine bax transcription without binding to an Ets-binding site	2005	WB	TAC-2.1	1:1000	No	88
893	# 16732981	Apoptosis induced by short hairpin RNA-mediated STAT6 gene silencing in human colon cancer cells	2006	WB	HT-29	?	No	89

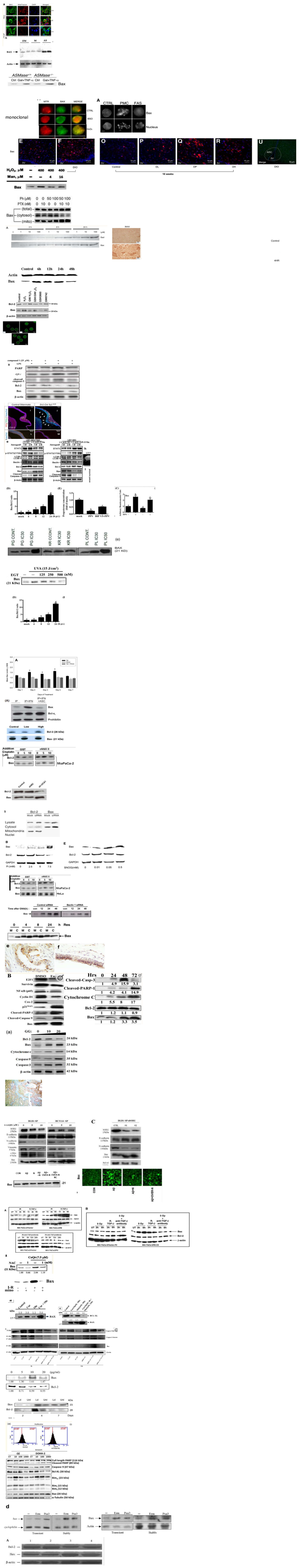

791	# 19130057	Activation of ER stress and inhibition of EGFR N-glycosylation by tunicamycin enhances susceptibility of human non-small cell lung cancer cells to erlotinib	2009	WB	H1650	?	No	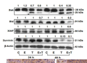	90
921	# 16136583	Expression of PCNA, p53, Bax, and Bcl-X in oral poorly differentiated and basaloid squamous cell carcinoma: relationships with prognosis	2005	IHC	basaloid & malignant cells of the PDSCC	1:100	No	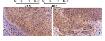	91
928	# 15831769	Modulation of apoptotic pathways in intestinal mucosa during hibernation	2005	WB	cytosolic or nuclear fractions from mucosa	?	No	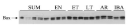	92
1065	# 11753684	Functional p53 is required for triptolide-induced apoptosis and AP-1 and nuclear factor-kappaB activation in gastric cancer cells	2001	WB	AGS	?	No	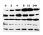	93
534	# 26085572	Bee venom suppresses testosterone-induced benign prostatic hyperplasia by regulating the inflammatory response and apoptosis	2015	WB	prostate tissue from Sprague-Dawley rats	1:1000	No	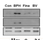	94
654	# 24330704	Eugenol triggers apoptosis in breast cancer cells through E2F1/survivin down-regulation	2013	WB	`MDA-MB-23, breast cancer xenografts	?	No	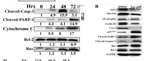	95
1082	# 10206987	Endostatin induces endothelial cell apoptosis	1999	WB	C-PAE	?	No	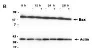	96
1156	# 32500380	Effect of di(2-ethylhexyl) phthalate on Nrf2-regulated glutathione homeostasis in mouse kidney	2020	WB	kidney tissue of mice	?	No	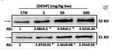	97
987	# 12810152	Biochemical studies of apoptosis induced by tamoxifen in estrogen receptor positive and negative breast cancer cell lines	2003	WB	MDA-MB-468; MCF-7	?	No	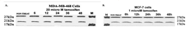	98
817	# 18027855	Fibroblast nemosis arrests growth and induces differentiation ofhuman leukemia cells	2008	WB	KG-1; THP-1; U937	?	No	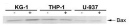	99
943	# 15728126	Farnesyltransferase inhibitor R115777 (Zarnestra, Tipifarnib) synergizes with paclitaxel to induce apoptosis and mitotic [illegible]	2005	WB	RPMI8226/S	?	No	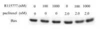	100

Supplementary Information 5: This list comprises a search for negative controls for BAX detection based on Santa Cruz antibody Bax (B-9): sc-7480 in studies listed on the current data sheet available at https://datasheets.scbt.com/sc-7480.pdf as of 18.12.2023

PMID	Title	Year	Methods used	Cell Line	Dilution	Negative Control	Notes
10206987	Endostain induces endothelial cell apoptosis.	1999	WB	C-PAE	?	No	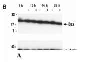
27108485	TLR2/NFkB signalling regulates endogenous IL-6 release from marrow-derived mesenchymal stromal cells to suppress the apoptosis of PC12 cells injured by oxygen and glucose deprivation.	2016	WB	MSCs from Wistar rats	1:1000	No	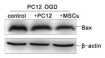
28160569	Mitotic cell death induction by targeting the mitotic spindle with Tubulin-inhibitory indole derivative molecules.	2017	WB	HeLa	1:500	No	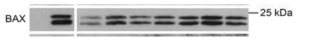
29389945	Intermittent hypoxia causes histological kidney damage and increases growth factor expression in a mouse model of obstructive sleep apnea.	2018	WB	kidney cortex from mice	1:100	No	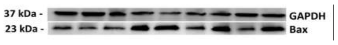
30864677	Effects of miR-103a-3p on the autophagy and apoptosis of cardiomyocytes by regulating Atg5.	2019	WB	H9c2	1:1000	No	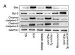
32018227	Baicalin alleviates benign prostate hyperplasia through androgen-dependent apoptosis.	2020	WB	prostatic tissues of rats	?	No	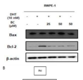
33868469	Siomycin A induces reactive oxygen species-mediated cytotoxicity in ovarian cancer cells.	2021	WB	PA1, OVCAR3	1:1000	No	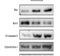
35639049	Gestational cholestasis induced intrauterine growth restriction through triggering IRE1α-mediated apoptosis of placental trophoblast cells.	2022	WB	Human/mouse placentas & trophoblast cells	?	No	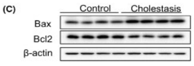
36641751	Targeting KRAS-mutant stomach/colorectal tumors by disrupting the ERK2-p53 complex.	2023	WB	GSU, AGS	?	No	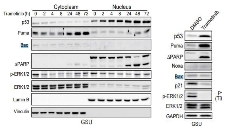
