# Supplementary material for: Why Bax detection in >1400 publications might be flawed
Source: Cell Death Dis. 2024 Dec 5;15(12):880. doi: 10.1038/s41419-024-07273-6 (PMC11621539; doi:10.1038/s41419-024-07273-6)

Fig.1A
Primary Antibodies, Loading Controls & Secondary Antibodies

- **In-house anti-Bax:** CST 2772; Lot:10; α-rb; 21 kDa; 1:1000
- **Anti-Bax**: (B-9) Santa Cruz #7480; α-ms; 21 kDa; 1:500
- **Anti-GAPDH**: CST, Art.-No.: 97166, Lot: #3, α-ms, 37 kDa, 1:2000
- **Secondary Antibodies:**

- α-rb: Jackson ImmunoResearch Laboratories Inc.; Code-No.: #111-035-144; Lot: #144841; stock concentration: 0.8 mg/mL; 1:10,000
- α-ms: Dianova; Lot: #143453; stock concentration: 0.8 mg/mL; 1:10,000

02.08.2023 **30µg protein**

Anti-Bax (B-9) sc-7480 1:500 monoclonal AA1-171 of Bax alpha mouse

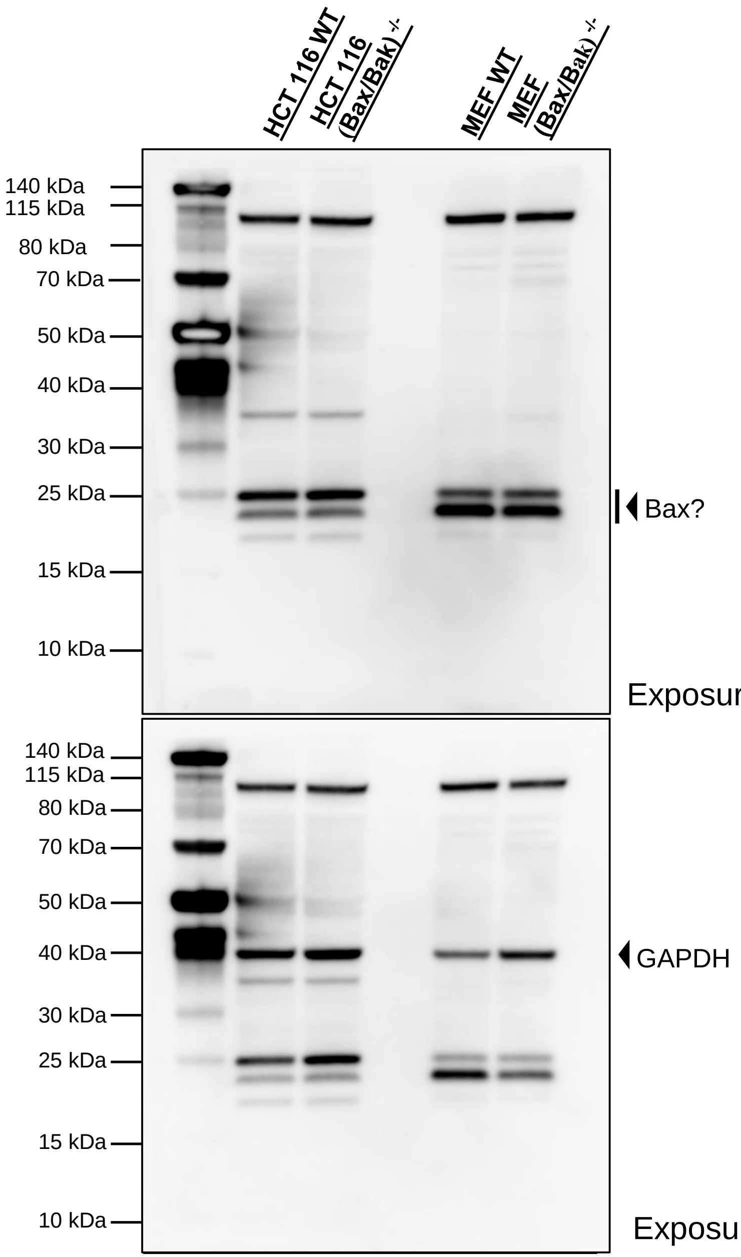

Exposure time: 5 sec

Exposure time: 30 sec

Anti-Bax CST #2772 1:1000 polyclonal against N-Terminus

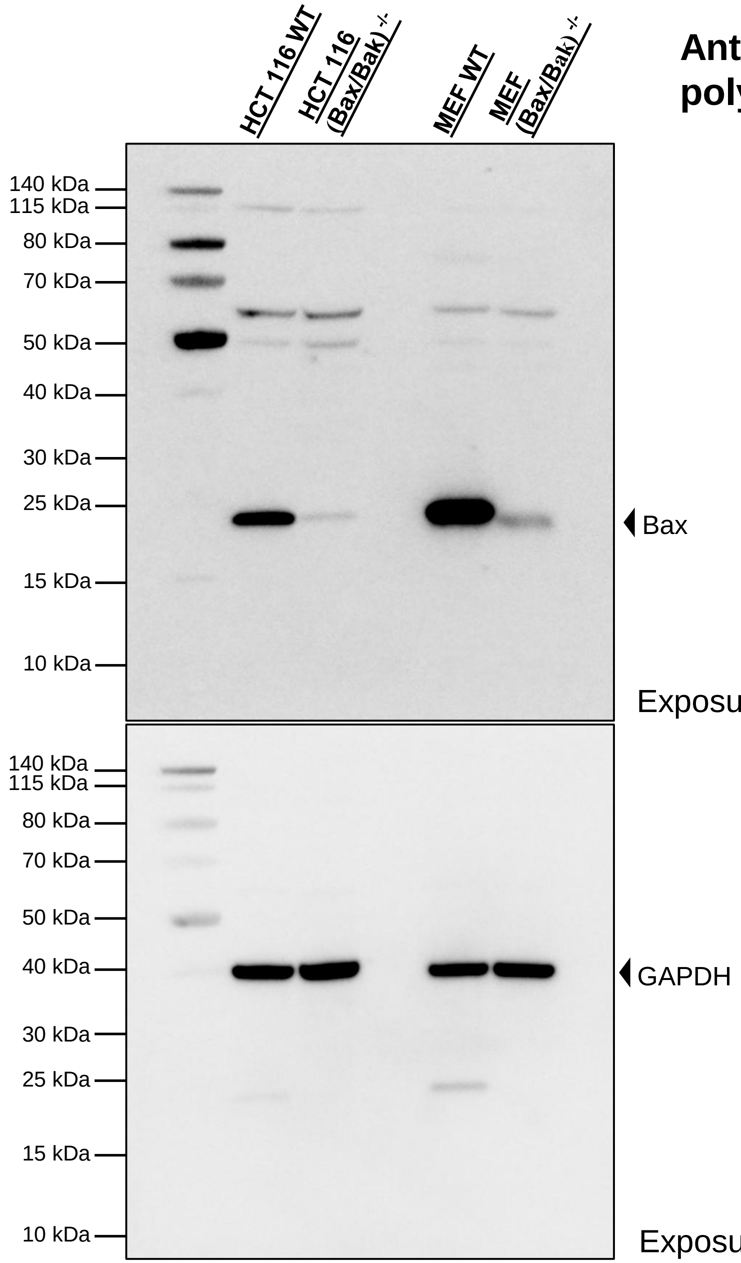

Exposure time: 10 sec

Exposure time: 30 sec

Fig.1B
Western Blot analysis of Bax expression in non-transfected control cells and Bax siRNA transfected HCT 116 WT cells

- Cell line: HCT 116 WT, 250,000 cells in one well of a 6 well plate
- siRNA: silencer select validated siRNA s1889 and s1890, Ambion, used concentration 30 pmol/well
- Negative control: NT (non-targeting) siRNA, Ambion, used concentration 30 pmol/well
- GAPDH used as negative control

07.12.2022 **n = 1** **30µg protein** aspect ratio slightly altered in Fig.1B to fit page

Anti-Bax (B-9) sc-7480
1:500
monoclonal AA1-171 of Bax alpha mouse

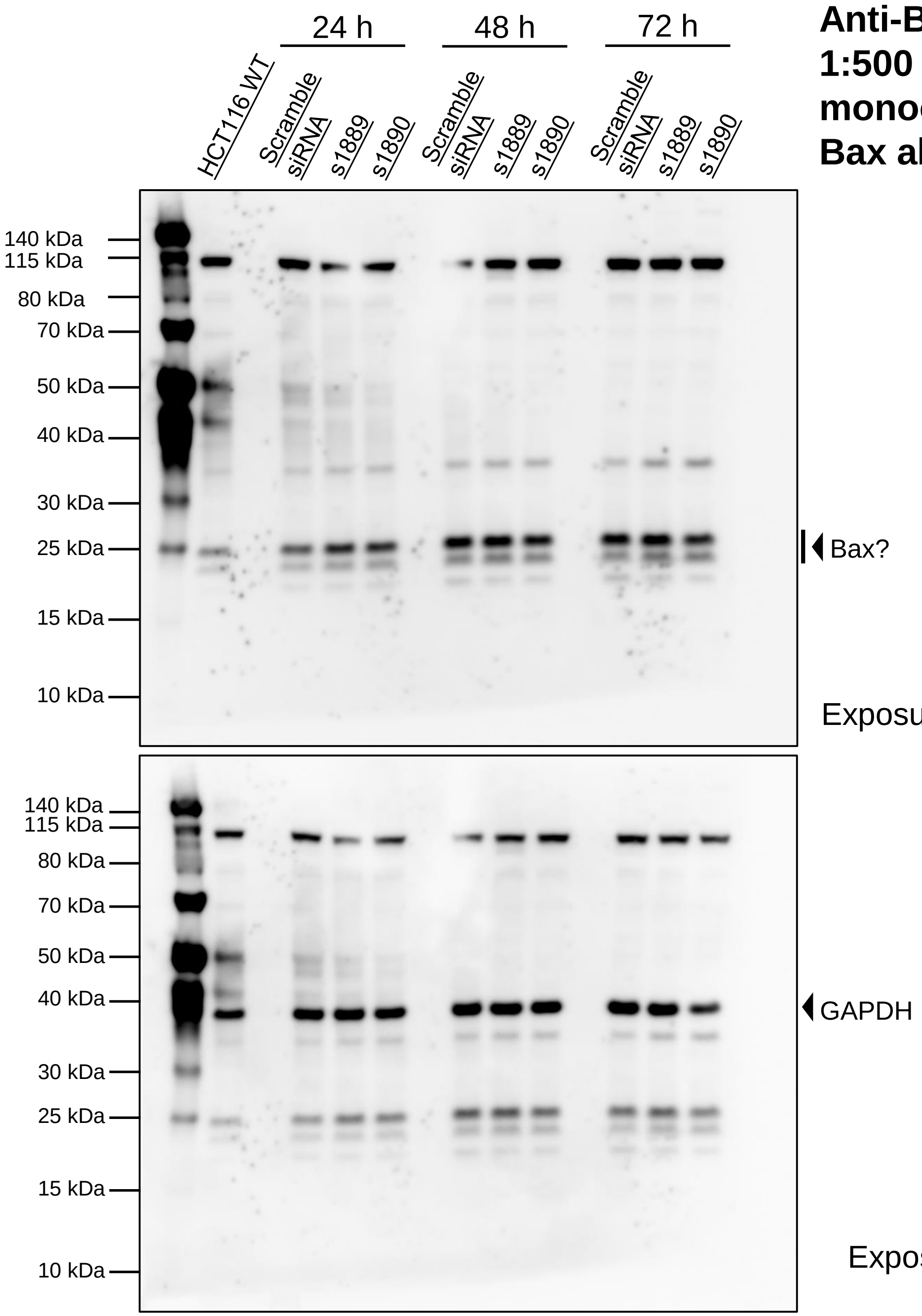

Anti-Bax CST #2772
1:1000
polyclonal against N-Terminus

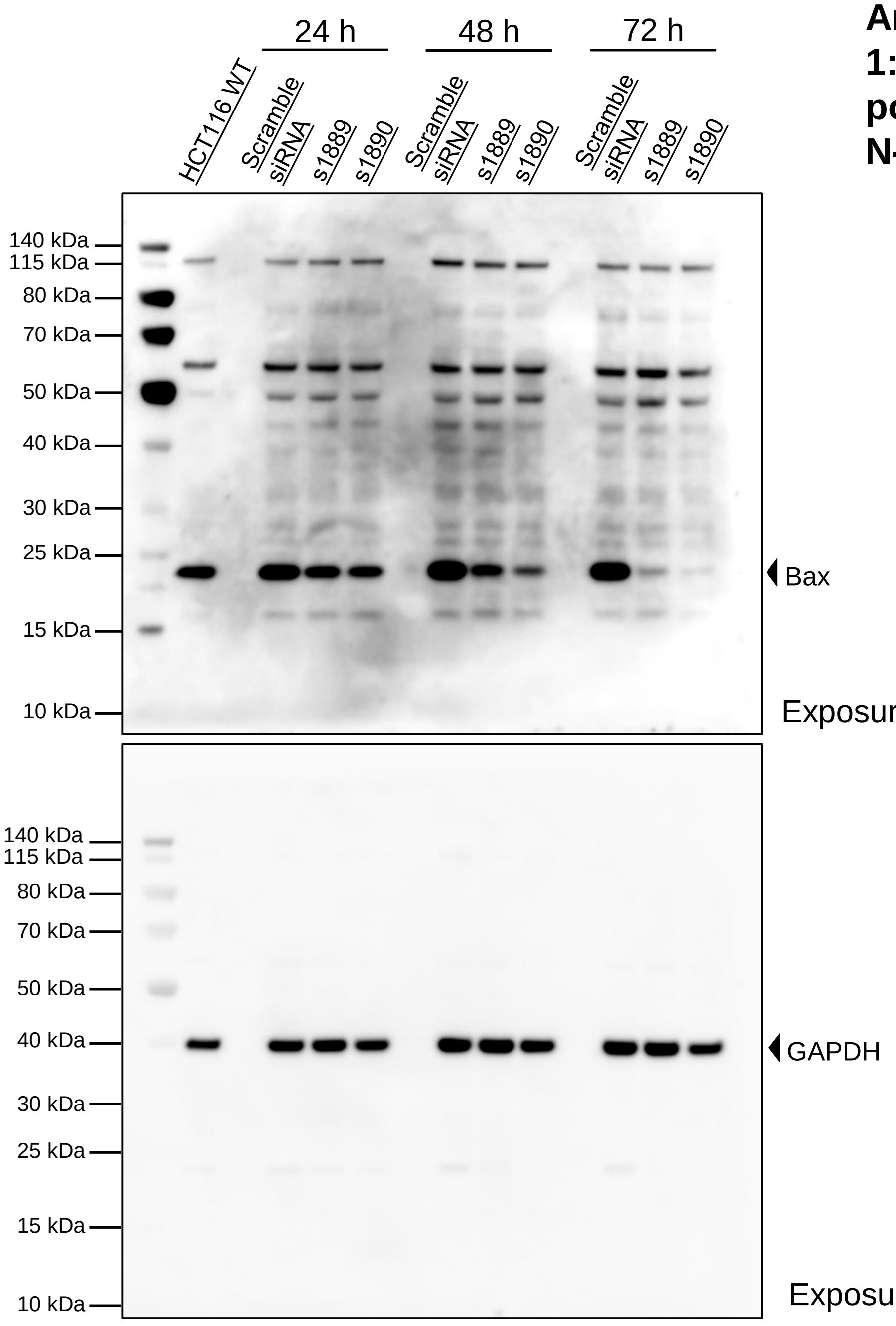

Supplement: Supplementary file 2 — Original Data [file 41419_2024_7273_MOESM2_ESM.pdf]
